# Estimating the size of “anti-vax” and vaccine hesitant populations in the US, UK, and Canada: comparative latent class modeling of vaccine attitudes

**DOI:** 10.1080/21645515.2021.2008214

**Published:** 2022-03-29

**Authors:** Timothy B. Gravelle, Joseph B. Phillips, Jason Reifler, Thomas J. Scotto

**Affiliations:** a Momentive, Aurora, Canada; b Laurier Institute for the Study of Public Opinion and Policy, Wilfrid Laurier University, Waterloo, Canada; c School of Psychology, University of Kent, canterbury, UK; d Department of Politics, University of Exeter, Exeter, UK; e School of Political and Social Sciences, University of Glasgow, Glasgow, UK

**Keywords:** vaccines, COVID, vaccine hesitancy, anti-vax, latent class modeling

## Abstract

Vaccine hesitancy is a significant impediment to global efforts to vaccinate against the SARS-CoV-2 virus at levels that generate herd immunity. In this article, we show the utility of an inductive approach – latent class analysis (LCA) – that allows us to characterize the size and nature of different vaccine attitude groups; and to compare how these groups differ across countries as well as across demographic subgroups within countries. We perform this analysis using original survey data collected in the US, UK, and Canada. We also show that these classes are strongly associated with SARS-CoV-2 vaccination intent and perceptions of the efficacy and safety of the COVID-19 vaccines, suggesting that attitudes about vaccines to fight the novel coronavirus pandemic are well explained by latent vaccine attitudes that precede the pandemic. More specifically, we find four substantive classes of vaccine attitudes: strong supporters, supporters with concerns, vaccine hesitant, and “anti-vax” as well as a fifth measurement error class. The strong “anti-vax” sentiment class is small in all three countries, while the strong supporter class is the largest across all three countries. We observe different distributions of class assignments in different demographic groups – most notably education and political leaning (partisanship and ideology).

## Introduction

The COVID-19 pandemic has given rise to an urgent global effort to vaccinate against the SARSCoV-2 virus. One significant concern is the extent to which vaccine hesitancy and “anti-vax” attitudes frustrate efforts to inoculate a large enough share of the population to contain COVID-19 and to reach herd immunity. Even before the pandemic, the World Health Organization (WHO) identified vaccine hesitancy as one of the top threats to public health.^1^ In this context, we use latent class modeling of online survey data to estimate the size and type of vaccine attitude clusters in the United States, United Kingdom, and Canada at a key point in time – shortly after the first COVID vaccines were approved for use and were being made available in these countries. Our approach helps address the recent observation that it is “vital to have serial, cross-sectional surveys that can identify issues within communities, countries, and regions”.^2^

The analyses presented here build on previous work in three key ways. First, we use an inductive data analysis approach, specifically latent-class modeling, to estimate the nature and size of different vaccine attitude groups in three different countries. While some previous work examining vaccine hesitancy has used this technique, we believe that it can be used more widely. Second, using identical questions across three different countries, our paper is among the first to use latent class analysis on vaccine attitudes cross-nationally. Finally, we are also able to make more explicit comparisons of vaccine attitudes across demographic subgroups within each country.

Although a sizable portion of people display some reservations about vaccines, only a small percentage in each country are truly “anti-vax” (we also observe cross-national differences). While previous research has also found that diehard “anti-vax” populations are relatively small, analyses can rely on arbitrary or ad-hoc cutoffs on an additive scale.^3^ Some work posits theoretical typologies without supporting empirical data. We believe that latent class analysis helps to clearly differentiate these groups using an inductive data analysis technique that has the advantage of model fit criteria and statistics. Using this approach, our model identifies five latent classes, with good comparability across the three countries. We find that these general attitudes toward vaccines – as defined by the latent classes – strongly correlate with intentions to receive a coronavirus vaccine, perceptions of coronavirus vaccine safety, and other beliefs related to the pandemic. While only those in the “anti-vax” class have unambiguously negative attitudes toward coronavirus vaccines, other classes vary considerably in coronavirus vaccine perceptions and intended uptake. Even those in a “vaccine hesitant” class report moderate intentions to get vaccinated. At the same time, some people who view vaccines favorably still have concerns, particularly around side effects. That there is such a large share of the public that possess a mix of both positive and negative beliefs toward vaccines suggests that elite messages – particularly from political leaders^4^ – could play a pivotal role in whether countries reach the high levels of vaccination necessary to confer the strongest public health benefits.^5^

## Prior work

Prior survey-based quantitative analysis of vaccine attitudes examines the factor structure of vaccine attitudes,^6,7,8^ establishes that vaccine attitude scales are clinically valid,^9^ investigates the attitudinal correlates of vaccine attitudes,^10^ explores potential informational causes of vaccine hesitancy,^11^ and assesses receptivity to information about vaccines.^10,12–15^ One study has even looked at “anti-vax” as a social identity.^16^ A persistent theme in the vaccine attitudes literature is that concerns about the safety and potential side effects of vaccines are a key barrier to universal uptake of vaccines,^17,18^ which comports with the findings we present below. Cross-national studies of vaccine hesitancy are still in their relative infancy,^19,20^ with at least one significant cross-national study of 67 countries,^21^ albeit with a limited question battery. (All survey batteries face trade-offs between concision and nuance). More recent work has looked at cross-national comparisons of vaccine attitudes through the lens of COVID.^22,23^

While previous research has long known that the proportion of the public that harbors extreme “anti-vax” views is small, inductive studies that estimate and establish the size of the vaccine hesitant and “anti-vax” communities through techniques such as latent class analysis are less common. When they do occur, they often have smaller samples or focus on specific populations rather than the general public as a whole. One large study of this type (n = 2,196) looked at vaccine attitudes and beliefs of pregnant women^23^ and identified three classes of vaccine attitudes, approximately one quarter of whom (23%) could be described as vaccine skeptics. Importantly, a follow-up study using the same sample found, though, even these skeptics improved their attitudes toward vaccines after a targeted information campaign.^24^ Other examples of latent class analysis of vaccine attitudes include a small sample (n = 189) study of parental attitudes toward vaccination in a Swiss canton^25^ and a larger sample (n = 431) examining parental concerns over HPV vaccination.^26^

Our approach contributes to research on vaccine attitudes in several important ways. First, our use of latent class modeling allows us to classify individuals into one of four clear classes of meaningful and distinguishable attitudes toward vaccines using a concise battery of seven questions (we also identify a fifth measurement error class). Second, we are able to validate these classes by demonstrating that they meaningfully correlate with demographic characteristics and attitudes about vaccine safety and intent. Third, we demonstrate that only a small (<10%) percentage of respondents in each country appears resolutely opposed to vaccines and vaccinations. Fourth, we show that there is a much larger “vaccine hesitant” class. Fifth, we show that the class structure is common to samples of citizens across three English-speaking nations. Sixth, we show that attitudes toward vaccines exhibit some polarization along political lines (partisanship and ideology). This last finding is particularly true for the US case, which also happens to be where we observe a larger share of the public combined in the “anti-vax” and “vaccine hesitant” classes than what we see in Canada or (especially) the United Kingdom.

## Methods

### Sample

We recruited 13,251 online respondents using Momentive’s endpage methodology. (At the time of data collection, Momentive was known as SurveyMonkey, which rebranded in June 2021) on January 5–19, 2021, comprising 4,612 from the United States, 4,089 from Canada, and 4,550 from the United Kingdom. After completing an unrelated survey on the Momentive platform, randomly selected respondents from the targeted countries (identified using their internet protocol (IP) addresses) received a survey completion web page (endpage) inviting them to then complete another survey. Samples were weighted to be demographically representative of the national adult populations in each country. Momentive’s endpage methodology was used for research on COVID-19 attitudes and behaviors previously.^26^ The participation rate, accounting for the number of survey invitations, click-throughs, and completed surveys is 3.5%. Our study was approved by the University of Exeter College of Social Sciences and International Studies Ethics Committee (IRB).

### Measures

All survey respondents received a questionnaire that included six items from the Parental Perspectives Regarding Vaccines scale^12,27^ plus one novel item – “Vaccinations are one of the most significant achievements in public health.” These items form a reliable scale (US: α = 0.87; UK: α = 0.84; Canada: α = 0.87). Questions administered after this scale assess vaccination intent, perceptions of the efficacy and safety of approved coronavirus vaccines, beliefs about COVID-19, political orientations, and demographic questions. The questionnaires appear in the online appendix materials as Appendix A.

### Statistical analysis

In contrast to most prior work, we seek to characterize attitudes toward vaccines into multiple categories rather than place the attitudes along a continuum or use arbitrary cutoff points to determine when one is vaccine hesitant. A substantial amount of work on this subject uses factor analysis, which is variable-centered and designed to assess whether different vaccine attitude items cohere into a reliable index at the sample-level. In contrast, latent class analysis is person-centered, and uses an inductive approach to uncover qualitatively different types of response patterns to two or more questions.^28^ What distinguishes anti-vax sentiment from other attitudes about vaccines may not simply be a more negative general attitude toward vaccines, but a more categorical negativity toward aspects of vaccination that vaccine hesitancy lacks. Latent class analysis is complementary to factor analysis and related techniques (e.g., IRT models), but is the more appropriate technique when trying to directly estimate the size of different underlying groups (or classes) in a population. For the specific purpose of estimating the type and size of different vaccine attitude groups, latent class analysis should be the preferred technique. We estimate our latent classes using Mplus version 8.1.^29^

As we have a three-country survey, we first ran separate LCA models for each country. As results were similar across countries, we performed multiple-group LCA^30^ with the observed response categories constrained to be equal across the American, British, and Canadian samples. These model constraints allow us to make direct comparisons across countries: since the latent classes are constrained to be statistically equivalent (and thus have the same substantive interpretation), we can compare directly their relative proportions across the three countries. The final model yielded 5 classes with a suitable ability to place respondents into classes (Entropy = 0.83). Likelihood-ratio tests indicated that extracting additional classes was not statistically justifiable. Below, we demonstrate that the model not only yields five interpretable classes, but that the analysis has both face and criterion validity in that the latent classes of vaccine attitudes have meaningful associations with the respondents’ demographic and political preferences, their January 2021 intentions to obtain the vaccine, and wider beliefs about vaccine mandates.

## Results

### Defining the latent classes

Table 1 presents the mean level of agreement with each vaccination attitude item on 5-point scales by latent class. All items are coded such that higher values represent more pro-vaccination answers. Means are generated from treating the ordinal variables from the latent class analysis as continuous for ease of presentation. Full statistical output files for the cross-national LCA as well as separate country-specific estimates appear in the online Appendix C. The first class, “Strong Support,” comprises people who strongly believe in the safety and effectiveness of vaccines. They do, however, have mild concerns about serious side effects of vaccines. The second class, “Support with Concerns,” is confident that vaccines are an important health tool. However, they are less keen to try new vaccines and are concerned about the safety and side effects of vaccines. The third class, the “Vaccine Hesitant,” tend to score at the mid-point of the scale on the importance of vaccines and following doctor recommendations. However, they are seriously concerned about side effects, support parents’ right to refuse vaccines for their children, and attach some credence to the notion that vaccines cause autism. The fourth and final substantive class, the “Anti-Vax,” tend to be skeptical of vaccines on all fronts and strongly support parents’ right to refuse vaccinations for their children. The final class is a “Measurement Error” class where respondents chose “strongly agree” for all items. This leads to high recorded support for vaccines, except on the reversed items.

**Table 1.** Vaccine attitudes by highest-probability assignment latent class. Top panel reports mean scores from a five-point Likert agree-disagree scale for each of the latent classes with each country weighted equally. Higher values reflect more pro-vaccine attitudes with reverse coded items indicated with an asterisk. Bottom panel reports the percentage in each class for each country (percentages should be read horizontally)

	Strongly support	Support w/concerns	Vaccine hesitant	Anti-vax	Measurement error
Getting vaccines is a good way to protect children from disease.	4.93	4.23	3.30	1.98	4.80
Generally I do what my doctor recommends about vaccines.	4.70	3.90	3.09	1.88	4.46
New vaccines are recommended only if they are safe.	4.57	3.85	3.14	2.11	4.53
I am concerned about serious side effects of vaccines.*	3.88	2.73	1.84	1.57	1.85
Some vaccines cause autism in healthy children.*	4.58	3.46	2.70	2.10	2.74
Parents should have the right to refuse vaccines required for schools for any reason.*	4.24	3.14	2.04	1.53	2.18
Vaccinations are one of the most significant achievements in improving public health.	4.88	4.08	3.14	1.76	4.76
*Class Membership by Country*					
USA (n = 4612)	34%	26%	23%	7%	10%
UK (n = 4550)	45%	31%	11%	3%	10%
Canada (n = 4089)	41%	28%	15%	7%	9%

For each country, the modal class comprises those who “strongly support” vaccines. Importantly, despite concerns about vaccines being common, a majority are in one of the two pro-vaccine classes (UK: 76%, Canada: 69%, and US 60%). In all three countries, the percentage classified as ardently “anti-vax” is very small. Only 7% of American and Canadian respondents and 3% of British respondents can be identified as anti-vax.

### Covariates

Table 2 shows the percentage assigned to the five classes for different demographic groups in each country. We focus on four demographic factors where there are notable differences in vaccine support across all three countries: education, age, and political ideology (or party). In the US, we also observe important differences by race.

**Table 2.** Substantive latent class membership by demographics and political orientation

		Strong support	Support w/concerns	Hesitant	Anti-vax
**USA**					
*Education*	No degree	26.2%	27.0%	28.3%	8.0%
	University degree	50.6%	25.0%	12.8%	4.0%
*Age*	18–24	38.0%	21.4%	20.5%	8.8%
	25–49	27.1%	24.1%	30.3%	8.4%
	50–64	32.2%	30.2%	22.3%	5.3%
	65 and over	47.1%	28.8%	13.1%	4.0%
*Ideology*	Very conservative	15.8%	20.6%	28.9%	17.9%
	Conservative	20.9%	34.1%	29.2%	7.5%
	Moderate	32.1%	27.6%	24.4%	5.2%
	Liberal	55.9%	21.5%	13.8%	2.7%
	Very liberal	66.9%	9.0%	9.8%	7.6%
*Party*	Republican	21.8%	31.2%	28.3%	9.6%
	Independent	19.3%	27.0%	31.9%	10.3%
	Democratic	51.5%	21.8%	14.8%	2.8%
*Race/Ethnicity*	White	39.9%	26.5%	19.9%	6.4%
	Black	13.2%	24.8%	37.9%	10.0%
	Hispanic	28.5%	29.5%	24.5%	5.0%
	Asian	31.2%	22.7%	18.9%	7.3%
	Other	16.8%	22.8%	43.8%	8.5%
**UK**					
*Education*	No degree	37.0%	35.1%	12.6%	3.2%
	University degree	61.6%	22.8%	6.3%	2.9%
*Age*	18–24	48.2%	29.0%	15.3%	2.1%
	25–49	38.4%	31.4%	14.9%	3.7%
	50–64	43.8%	33.3%	9.4%	2.2%
	65 and over	56.3%	28.8%	2.2%	3.4%
*Ideology*	Right (5)	37.8%	29.3%	11.2%	4.4%
	4	50.0%	31.5%	8.1%	3.5%
	3	37.9%	35.3%	12.3%	3.1%
	2	61.6%	24.2%	6.3%	1.9%
	Left (1)	55.5%	21.8%	9.6%	2.3%
*Party*	Conservative	48.3%	31.9%	5.6%	2.1%
	Labour	51.6%	28.5%	8.7%	2.1%
	None	29.4%	35.3%	18.6%	4.8%
	Liberal Democrat	57.8%	27.8%	5.9%	1.6%
	Green	63.5%	18.0%	7.5%	4.5%
	Scottish National Party	46.3%	35.1%	9.4%	4.0%
	Brexit/UKIP	21.1%	35.5%	20.1%	4.9%
	Other party/No party	33.3%	34.0%	17.1%	4.8%
**Canada**					
*Education*	No degree	36.8%	29.5%	17.4%	7.3%
	University degree	52.1%	24.3%	10.2%	4.6%
*Age*	18–24	42.6%	29.2%	15.0%	5.2%
	25–49	34.2%	27.4%	19.8%	8.4%
	50–64	40.1%	30.4%	14.9%	6.3%
	65 and over	55.8%	25.5%	7.3%	3.8%
*Ideology*	Right (5)	20.6%	22.5%	25.8%	15.0%
	4	38.5%	26.0%	21.0%	8.2%
	3	35.9%	31.8%	16.0%	6.3%
	2	62.3%	23.3%	6.8%	2.9%
	Left (1)	61.5%	19.0%	8.7%	3.2%
*Party*	Liberal	53.8%	23.2%	8.3%	2.7%
	Conservative	34.9%	32.6%	19.4%	7.4%
	New Democratic Party	56.3%	22.7%	9.9%	3.5%
	Green	50.3%	26.7%	10.8%	6.2%
	Bloc Québécois	44.2%	31.2%	13.4%	2.9%
	Other party/No party	26.7%	30.4%	21.9%	11.0%

Percentages should be read horizontally, which indicate the percentage of each demographic group in each of the four substantive classes. Totals add to less than a 100% because the measurement error class is not presented.

Across countries, university-educated respondents are more likely to be in a supportive class than those without degrees. As for age, in the US, the youngest respondents are both the second-most likely to be in the Strong Support class and the most likely to be Anti-Vax, indicating they may be more polarized in their vaccine attitudes relative to other age groups. Hesitancy is most common among those aged 25–49 – the most likely group to be parents of young children. Of those aged 50–64, it becomes more common to broadly support vaccines, but have concerns. The oldest respondents are highly supportive of vaccines. In the UK and Canada, differences by age are less pronounced, but older respondents are consistently more likely to be in the Strong Support class. When it comes to ideology, we observe associations with class assignment. People who are on the left side of the political spectrum are more likely than those on the political right (especially the far right) to belong to the strong vaccine supporter class. (Note that question wording on ideology differs across countries. The US sample are asked, “In general, how would you describe your views on most political issues? Are you …,” answering on a scale from 1 (very liberal) to 5 (very conservative). The UK and Canada samples are asked, “In politics people sometimes talk of left and right. Where would you place yourself on the following scale?” from 1 (very left) to 5 (very right).) Those who are “very conservative” or furthest to the “right” are especially likely to be in the vaccine hesitant or anti-vax classes. Moderates are still broadly supportive of vaccines, but are more likely to display support with concerns than ideologues, left or right. These results are generally consistent with existing research in the US and Canada showing that those on the political right express less concern over COVID-19 and less willingness to follow direction from public health officials.^31,32^

Finally, in the US sample, we also observe differences in vaccination attitudes by race. A plurality of white and Asian-American respondents fall into the “strongly support” category. Hispanic respondents also are fairly likely to give broad support to vaccines, though they are somewhat more likely to combine that support with some concerns than their White or Asian-American counterparts. The plurality of Black respondents and respondents of other races fall into the “Hesitant” category. This finding broadly consistent with other work showing that Black Americans display above-average vaccine hesitancy.^33^ Since we did not measure ethnicity in the British or Canadian samples, we cannot compare attitudes cross-nationally by ethnic and racial origin subgroups.

In summary, our latent class analysis reveals four substantively clear patterns of vaccine attitudes across three countries, and with fairly predictable demographic predictors. Across countries, respondents who have higher levels of education, are over the age of 65, and are on the left wing of the political spectrum are better represented in the vaccine-supportive classes.

However, there are key differences between countries as well. The first is that fewer British respondents exhibit anti-vax sentiment or vaccine hesitancy than American and Canadian respondents. While we can only speculate on the reason for this difference, we submit that part of it is a lack of salient partisan divisions over vaccination in Britain compared to other countries. Both Conservative and Labour supporters are well-represented in the vaccine-supportive classes. This consensus seems to exist despite the fact that left-wing respondents are still disproportionately supportive of vaccines. The only partisan groups who report high levels of hesitancy are those who do not identify with any party at all and those who identify with the Brexit Party (now Reform UK) or the UK Independence Party (UKIP). In contrast, Democrats in the United States are more likely to be assigned to vaccine-supportive classes than Republicans or Independents. In Canada, partisan divisions, while not sharp, are still present. Conservative supporters tend to express more Vaccine Hesitant or Anti-Vax sentiment.

These latent classes appear to capture demographic and political divisions over vaccination that are in line with other research.^11,13,14,16,21,34^ However, it remains unclear whether these latent classes vary in predictable ways on criterion measures. Furthermore, it is unclear how much even a little vaccine hesitancy can affect vaccine uptake, particularly of the COVID-19 vaccine just as it became available for emergency use. Therefore, in the upcoming sections, we turn to examining latent class differences in a number of vaccine attitudes and behaviors.

### Support for vaccine mandates by class

In each of our surveys, we included a 7-item scale of support for mandating vaccines. Respondents were given the stem, “Do you support or oppose …” with the items “Health authorities making vaccinations mandatory to attend large public events like concerts and sporting events?” “Airlines requiring individuals to be vaccinated to travel internationally?” “Companies having the right to fire employees who refuse to get a coronavirus vaccine?” “Companies having the right to require employees to be vaccinated before they can physically return to the workplace?” “Health authorities making vaccinations mandatory to use trains and buses?” “A government requirement for individuals to be vaccinated to enter [the respondent’s country] from abroad?” “Health authorities making vaccinations mandatory for everyone who can be safely vaccinated?” Respondents answered on 4-point scales from 1 (Strongly Oppose) to 4 (Strongly Support). These items cohere into a highly reliable scale (US: α = 0.95; UK: α = 0.93; Canada: α = 0.95).

**Figure 1.**
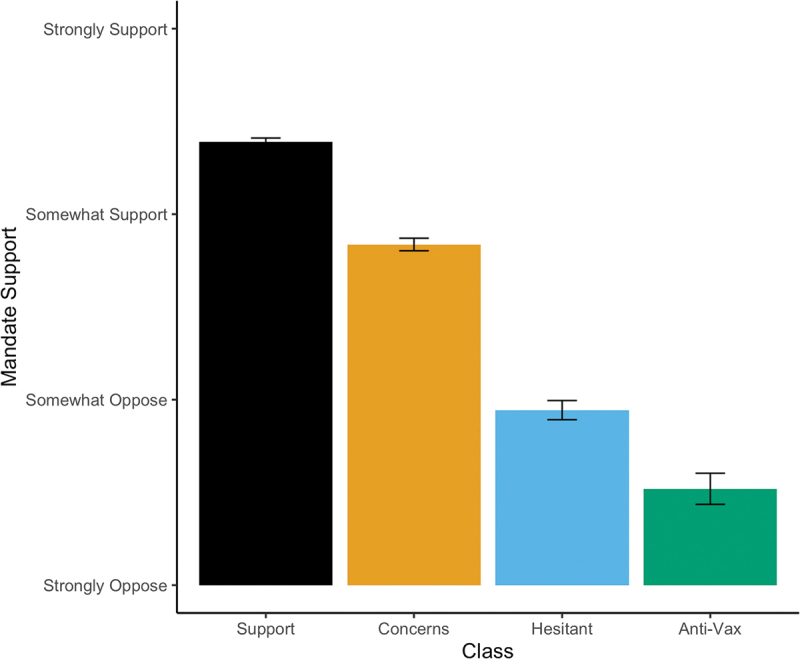
Support for vaccine mandates by substantive class (4-point Likert scale). The Strong Support and Support with Concerns classes support vaccine mandates dramatically more than the Vaccine Hesitant or Anti-Vax classes.

We depict support for vaccine mandates by substantive latent class in Figure 1, treating the 4-point mandate items as continuous and averaging them across respondents. Class membership strongly tracks with support for vaccine mandates. The “Strong Support” class is the most supportive, between “somewhat” and “strongly” supportive of most items. The “Support with Concerns” class is more tepid, only somewhat supporting the average vaccine mandate (combined measurement). There is a much more significant drop moving from “Support with Concerns” to the “Vaccine Hesitant.” This class tends to oppose most vaccination mandates but only somewhat. Predictably, the “Anti-Vax” class is the most stridently opposed to any sort of vaccine mandate.

### COVID attitudes and behaviors by class

In our surveys, we included a number of items related to the COVID-19 pandemic. First, we included a battery of COVID-19 conspiracies. Respondents answered 5-point items from 1 (Definitely not true) to 5 (definitely true) on five different coronavirus conspiracy theories. The items had the stem “Some people believe that [conspiracy theory]. Others do not believe this. What do you think? Is [theory] … ” The items were “The coronavirus was accidentally released from a laboratory in China,” (LabChn) “The coronavirus was intentionally created as a plot to reduce the world’s population,” (PlotRedu) “5G technology is causing the coronavirus to spread faster,” (Spread5G) “Coronavirus is actually a biological weapon that was released from a laboratory in China,” (BiowpChn) and “The coronavirus isn’t real, and that doctors and scientists are in on the elaborate hoax.” (CoroHoax).

**Figure 2.**
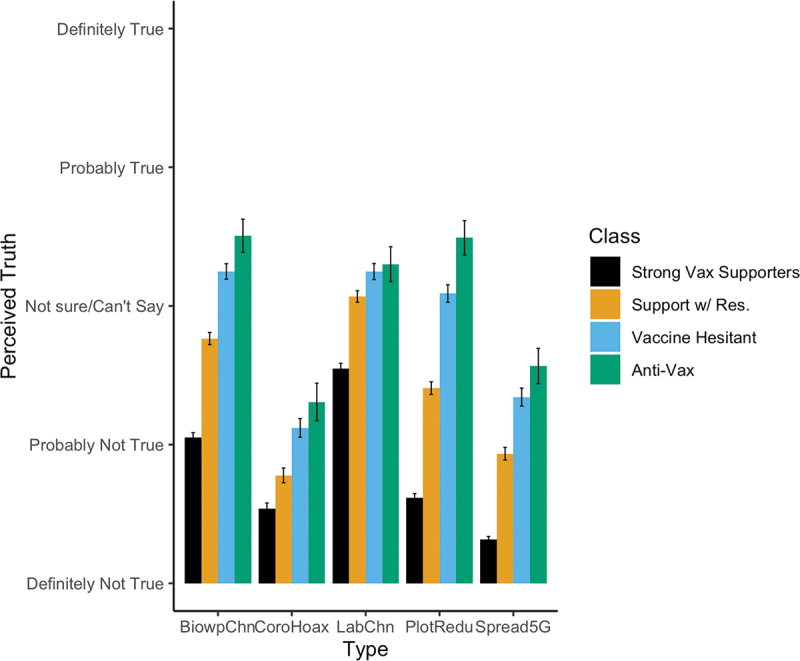
Belief in COVID-19 conspiracy theories (5-point Likert items) by substantive class. The Anti-Vax and Vaccine Hesitant subscribe to COVID-19 conspiracy theories more than other classes, especially the Strong Support class.

In Figure 2, we document belief in each of these conspiracy theories by substantive latent class. For each individual conspiracy, we see a strong correspondence between class membership and belief in COVID-19 conspiracy theories across items. The “Anti-Vax” group tends to consider them the most true, followed closely by the “Vaccine Hesitant” class. With the exception of coronavirus being a hoax, those in the “Support with Concerns” Group are significantly more likely to lend credence to coronavirus conspiracy theories than the “Strong Support” group.

We also fielded a number of items on the COVID-19 vaccine. Two were on perceived vaccine safety and effectiveness. Respondents were given the stem, “Based on what you know or have heard, do you think the approved coronavirus vaccine is …” and answered on 5-point scales from 1 (Not at all safe/effective) to 5 (Very safe/effective).

**Figure 3.**
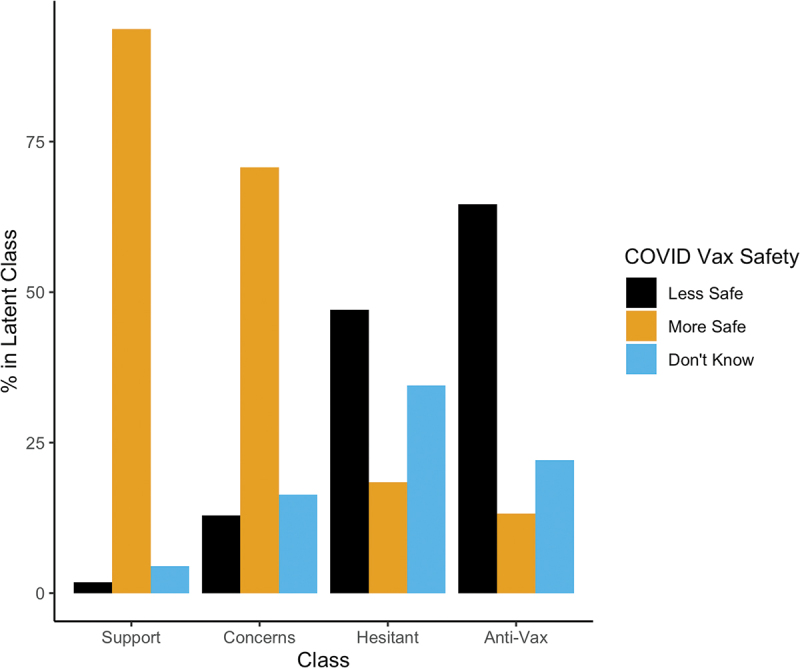
Percentage of each latent class in each COVID safety perception category. Low safety ratings include “not at all safe” or “not very safe.” High safety ratings include “somewhat safe” and “very safe.” Don’t know responses depicted separately. The Strong Support and Support with Concerns classes broadly view COVID-19 vaccines as safe, while the Anti-Vax see them as unsafe. The Vaccine Hesitant are more unsure about COVID-19 vaccine safety.

**Figure 4.**
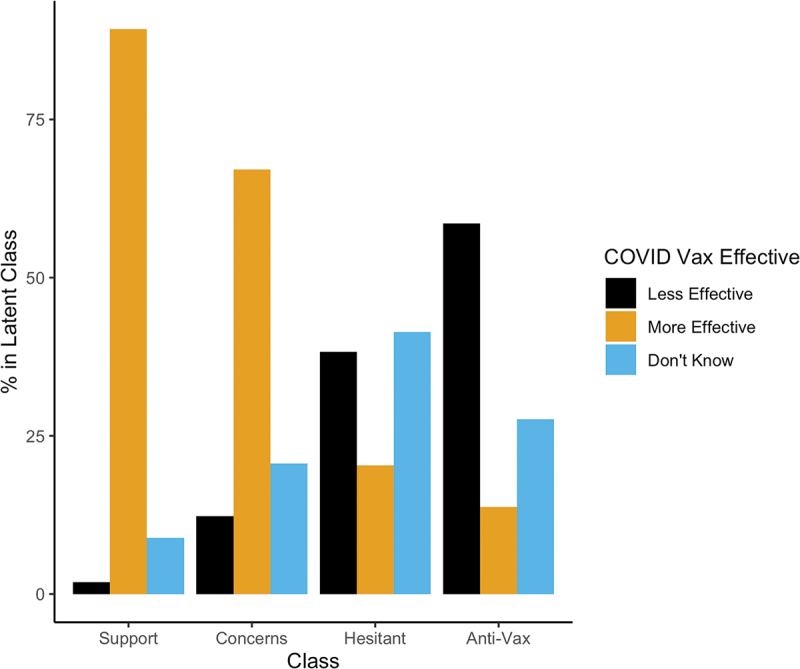
Percentage of each latent class in each COVID effectiveness perception category. Low effectiveness ratings include “not at all effective” or “not very effective.” High effectiveness ratings include “somewhat effective” and “very effective.” Don’t know responses depicted separately. The Strong Support and Support with Concerns classes broadly view COVID-19 vaccines as effective, while the Anti-Vax see them as ineffective. The Vaccine Hesitant are more unsure about COVID-19 vaccine effectiveness.

Figures 3 and 4 depict perceived vaccine safety and efficacy, respectively, by substantive class. For ease of presentation, we collapsed “not at all,” “not very,” and “slightly” safe/effective answers into a “less safe/effective” category and “somewhat” and “very” safe/effective into a “more effective” category. Perceived COVID vaccine safety and effectiveness track strongly with latent class membership. The more supportive one is of vaccines generally, the more people see the COVID vaccine as generally safe and effective. Additionally, our latent class analysis picks up qualitative differences between the “Vaccine Hesitant” class and the “Anti-vax” class. Whereas a majority of the “Anti-vax” believe the COVID-19 vaccine is unsafe, the “Vaccine Hesitant” are quite likely to report being unsure about COVID-19 vaccine safety and effectiveness.

**Figure 5.**
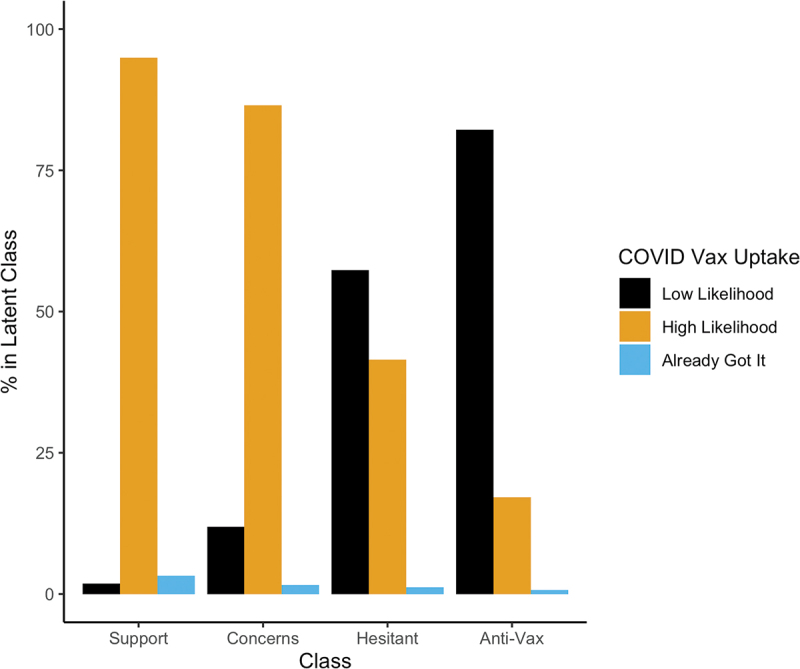
Percentage of each latent class in each COVID vaccine uptake intention category. Low likelihood ratings include “not at all likely” or “not very likely.” High likelihood ratings include “somewhat likely,” “very likely,” or “extremely likely.” The small group of respondents who already got the vaccine are in their own category. The Strong Support and Support with Concerns classes largely plan to get vaccinated, and the Anti-Vax do not. The Vaccine Hesitant are more evenly divided.

Finally, as this survey was fielded when only a small proportion of Americans, Britons and Canadians were eligible for vaccines, we had respondents answer a single 5-point item measuring likelihood of receiving the approved coronavirus vaccine. We depict these results by substantive latent class in Figure 5. For ease of presentation, we collapsed the “not at all” through “somewhat” likely categories into a “low likelihood” category and the “very” and “extremely” likely categories into a “high likelihood” category. We see a strong dividing line between the “Strong Support”/“Support with Concerns” classes and the “Vaccine Hesitancy”/“Anti-Vax” classes. The “Strong Support” class predictably has a rather high likelihood of getting the COVID-19 vaccine. However, even the “Support with Concerns” category, when it comes to the important decision of getting a COVID vaccine, is nearly as willing to take one. The “Vaccine Hesitant” class are somewhat split between those with low and high likelihoods of getting the vaccine. Predictably, the “Anti-Vax” are not particularly likely to take a vaccine. A small proportion of our respondents already received the vaccine. The likelihood of already having gotten the vaccine tracks well with latent class – the more supportive one is of vaccines, the more likely one is to have gotten the COVID vaccine already.

## Discussion

In this paper, we presented a latent class analysis of vaccine attitudes. Across three separate countries, we found that a substantial proportion of the public expresses concerns about vaccines, but support is more widespread. Between the “Support with Concerns” and “Vaccine Hesitant” classes, a large percentage of the public in each country has mixed attitudes toward vaccines. Given these conflicting attitudes, messages from key elites – not merely political elites but also leaders from the scientific community – may have outsized effects on whether countries will meet public health campaign goals for COVID-19 vaccine uptake.^35^ This finding is especially worrying in the United States – which has the largest “Vaccine Hesitant” and “Anti-Vax” segments to begin with – due to the prevalence of anti-vaccination messages coming from key political and media leaders. Worryingly, vaccines seem to be emerging as a key “culture war” issue at least in the United States, which could have devastating effects on public health over time. In the specific context of COVID, the effects of underimmunization have been severe. As of this writing, the United States continues to lag behind other advanced democracies in terms of the percentage of the population vaccinated and there is well organized resistance to vaccine mandates. Frustrated with the pace of vaccination, Democratic US President Joe Biden, reinforced by Democratic governors in many “blue” states, set mandates for employees of large firms to require vaccination. Follow-up studies should determine whether this (or perhaps lesser incentives) is what is needed to bring those in the “Hesitancy” class into the fold and whether all or some of those in the “Anti-Vax” class will sacrifice their economic livelihoods due to their beliefs about the risks associated with vaccination.

Our study has multiple strengths. The large-scale samples we use allow us to distinguish between subtypes of vaccine attitudes in a relatively fine-grained manner. The fact that our samples come from multiple countries allows us to assess how subgroup differences may or may not travel from one context to another. However, our study has some key limitations as well. First, though our samples are large and high-quality, they are non-probability samples. Like item response theory models, latent class analyses are not sample-invariant, so caution should be used when generalizing these findings to their respective populations. Second, our samples all come from Anglo-American democracies, which means we cannot generalize these findings to non-Anglo or non-western contexts. Future work should take up this mantle. Third, though we expect our latent classes to widely apply to specific vaccine attitudes other than COVID-19 vaccines, we cannot test this directly with our data. Finally, our analysis uses a particular scale measuring vaccine attitudes (PPRV), and many other such scales exist (such as the PACV and the Vaccine Hesitancy Scale).^6,9^ We strongly encourage future work to apply latent-class techniques to other scales measuring vaccine hesitancy. If the application of these data analysis techniques to the survey data are – as we suspect – effectively revealing different types of vaccine attitudes, we would expect that different scales utilized in the same time and place would produce substantially similar latent class estimates. We strongly encourage future research to examine whether this conjecture is accurate, and just how much question choice affects our conclusions about the nature of people’s attitudes about vaccines.

We believe this latent class approach contributes on a number of fronts. First, we show scales originally developed to understand vaccination attitudes of parents appear to sufficiently tap underlying vaccine attitudes that are strongly associated in predictable ways with COVID-19 attitudes. Future work testing pro-vaccination messages may wish to employ these techniques to test for heterogeneous treatment effects across these vaccine classes, especially as linear interaction terms may not always be appropriate.^33,36^ Similarly, this approach may be useful for screening respondents to micro-target persuasive communications more efficiently.

## Supplementary Material

Supplemental Material
