# Supplementary material for: Estimating the size of “anti-vax” and vaccine hesitant populations in the US, UK, and Canada: comparative latent class modeling of vaccine attitudes
Source: Hum Vaccin Immunother. 2022 Mar 29;18(1):2008214. doi: 10.1080/21645515.2021.2008214 (PMC9009899; doi:10.1080/21645515.2021.2008214)
Supplement: Supplemental Material [file KHVI_A_2008214_SM3525.docx]

**Appendix A: Questionnaire**

**Vaccine scale questions**

Do you agree or disagree with the following statements?

I am concerned about serious side effects of vaccines

Parents should have the right to refuse vaccines required for schools for any reason.

Some vaccines cause autism in healthy children

Enough children are vaccinated that even unvaccinated children are safe from disease.

Vaccinations are one of the most significant achievements in improving public health.

New vaccines are recommended only if they are safe.

Generally, I do what my doctor recommends about vaccines.

Getting vaccines is a good way to protect children from disease. S

Strongly agree

Agree

Neither agree nor disagree

Disagree

Strongly disagree

**Coronavirus vaccination intent**

A vaccine for the coronavirus has been approved by the [US: Food and Drug Administration (FDA)] [UK: Medicines and Healthcare products Regulatory Agency (MHRA)] [Canada: Health Canada]. How likely is it that you will choose to get an approved vaccine when you are eligible?

Extremely likely

Very likely

Somewhat likely

Not very likely

Not at all likely

I have already gotten a vaccine

**Coronavirus vaccine efficacy and safety perceptions**

Based on everything you know or have heard, would you say approved coronavirus vaccines are:

Very effective

Somewhat effective

Slightly effective

Not very effective

Not at all effective

Don’t know/Not sure

Based on everything you know or have heard, would you say approved coronavirus vaccines are:

Very safe

Somewhat safe

Slightly safe

Not very safe

Not at all safe

Don’t know/Not sure

**Specific vaccine preference**

Assuming different types of vaccines are approved for use by the [US: Food and Drug Administration (FDA)] [UK: Medicines and Healthcare products Regulatory Agency (MHRA)] [Canada: Health Canada], would you say you have a preference for one type of vaccine over another?

I do not prefer a specific vaccine, and will take whatever is available first.

I prefer a specific vaccine, but will take whatever is available first.

I prefer a specific vaccine, and will wait for that one to be available.

I do not plan to get vaccinated.

Don’t know/Not sure

**Coronavirus vaccination policy questions**

As coronavirus vaccines become more available, there has been discussion about requiring vaccinations for certain activities (except for those who cannot get vaccinated due to health reasons, or who refuse on religious grounds).

Do you support or oppose health authorities making vaccinations mandatory to attend large public events like concerts and sporting events?

Strongly support

Somewhat support

Somewhat oppose

Strongly oppose

Do you support or oppose airlines requiring individuals to be vaccinated to travel internationally?

Strongly support

Somewhat support

Somewhat oppose

Strongly oppose

Do you support or oppose companies having the right to fire employees who refuse to get a coronavirus vaccine?

Strongly support

Somewhat support

Somewhat oppose

Strongly oppose

Do you support or oppose companies having the right to require employees to be vaccinated before they can physically return to the workplace?

Strongly support

Somewhat support

Somewhat oppose

Strongly oppose

Do you support or oppose health authorities making vaccinations mandatory to use trains and buses?

Strongly support

Somewhat support

Somewhat oppose

Strongly oppose

Do you support or oppose a government requirement for individuals to be vaccinated to enter [US: the United States] [UK: the United Kingdom] [Canada: Canada] from abroad?

Strongly support

Somewhat support

Somewhat oppose

Strongly oppose

Do you support or oppose health authorities making vaccinations mandatory for everyone who can be safely vaccinated?

Strongly support

Somewhat support

Somewhat oppose

Strongly oppose

**Coronavirus conspiracy belief questions**

Some people believe that the coronavirus is actually a biological weapon that was released from a laboratory in China. Others do not believe this. What do you think? Is this...?

Definitely true

Probably true

Not sure/Can’t say

Probably not true

Definitely not true

Some people believe that the coronavirus was accidentally released from a laboratory in China. Others do not believe this. What do you think? Is this...?

Definitely true

Probably true

Not sure/Can’t say

Probably not true

Definitely not true

Some people believe that the coronavirus was intentionally created as a plot to reduce the world’s population. Others do not believe this. What do you think? Is this...?

Definitely true

Probably true

Not sure/Can’t say

Probably not true

Definitely not true

Some people believe that 5G technology is causing the coronavirus to spread faster. Others do not believe this. What do you think? Is this...?

Definitely true

Probably true

Not sure/Can’t say

Probably not true

Definitely not true

Some people believe that the coronavirus isn’t real, and that doctors and scientists are in on the elaborate hoax. Others do not believe this. What do you think? Is the coronavirus..?

Definitely true

Probably true

Not sure/Can’t say

Probably not true

Definitely not true

**Respondent Attributes**

[US only] In general, how would you describe your views on most political issues? Are you:

Very conservative

Conservative

Moderate

Liberal

Very liberal

[US only] In politics today, do you consider yourself a Republican, Democrat, or Independent?

Republican

Democrat

Independent

[US only; if party identification = "Independent"] As of today, do you lean more to the Republican Party or more to the Democratic Party?

Republican

Democrat

Neither

[UK only] Generally speaking, do you think of yourself as Labour, Conservative, Liberal Democrat or another party?

Conservative

Labour

Liberal Democrat

Scottish National Party (SNP)

Plaid Cymru

Brexit Party

United Kingdom Independence Party (UKIP)

Green Party

British National Party (BNP)

No - None Other (please specify)

[Canada only] In federal politics today, with which party do you most closely identify?

Bloc Québécois

Conservative Party of Canada

Green Party of Canada

Liberal Party of Canada

New Democratic Party (NDP)

None Other (please specify)

[UK and Canada only] In politics people sometimes talk of left and right. Where would you place yourself on the following scale?

1 - Left 2 3 4 5 – Right

Which gender best describes you?

Male

Female

Not listed/non-conforming

What is your age?

[US only] Are you:

White

Black

Hispanic

Asian

Other

[US only] What is the last grade of school you completed?

Did not complete high school

High school or G.E.D.

Associate’s degree

Some college

College graduate

Post-graduate degree

[UK only] What is the highest level of school you have completed or the highest degree you have received?

None

O level, GCSE or equivalent

A level or equivalent

Higher National Certificate (HNC, BTEC), Trade or equivalent

Bachelor’s degree

Postgraduate degree or above

[Canada only] What is the highest level of education you have attained?

Less than high school

High school

College certificate or diploma/CEGEP

Bachelor’s degree

Graduate degree

**Appendix B: Further Analyses**

**Table B1**: Vaccine policy answers by latent class and country (% somewhat or strongly support)

| Do you support or oppose... | Strongly Support | Support w/Concerns | Vaccine Hesitant | Anti-Vax |
| --- | --- | --- | --- | --- |
| **USA** |  |  |  |  |
| Health authorities making vaccinations mandatory to attend large public events like concerts and sporting events? | 87.2% | 64.5% | 27.9% | 12.2% |
| Airlines requiring individuals to be vaccinated to travel internationally? | 93.2% | 72.5% | 37.8% | 17.8% |
| Companies having the right to fire employees who refuse to get a coronavirus vaccine? | 62.6% | 33.2% | 17.2% | 9.0% |
| Companies having the right to require employees to be vaccinated before they can physically return to the workplace? | 87.6% | 59.3% | 27.4% | 11.9% |
| Health authorities making vaccinations mandatory to use trains and buses? | 83.7% | 61.4% | 26.9% | 13.1% |
| A government requirement for individuals to be vaccinated to enter [the respondent's country] from abroad? | 94.6% | 81.4% | 50.1% | 28.5% |
| Health authorities making vaccinations mandatory for everyone who can be safely vaccinated? | 85.3% | 57.4% | 28.1% | 11.0% |
|  |  |  |  |  |
| **UK** |  |  |  |  |
| Health authorities making vaccinations mandatory to attend large public events like concerts and sporting events? | 89.7% | 79.2% | 41.6% | 24.6% |
| Airlines requiring individuals to be vaccinated to travel internationally? | 93.9% | 89.0% | 46.7% | 27.4% |
| Companies having the right to fire employees who refuse to get a coronavirus vaccine? | 53.0% | 41.2% | 25.5% | 13.5% |
| Companies having the right to require employees to be vaccinated before they can physically return to the workplace? | 84.6% | 72.3% | 36.2% | 19.7% |
| Health authorities making vaccinations mandatory to use trains and buses? | 83.3% | 73.8% | 39.2% | 23.5% |
| A government requirement for individuals to be vaccinated to enter [the respondent's country] from abroad? | 93.3% | 87.4% | 56.4% | 39.4% |
| Health authorities making vaccinations mandatory for everyone who can be safely vaccinated? | 85.1% | 74.3% | 38.8% | 26.6% |
|  |  |  |  |  |
| **Canada** |  |  |  |  |
| Health authorities making vaccinations mandatory to attend large public events like concerts and sporting events? | 90.5% | 69.6% | 30.9% | 10.5% |
| Airlines requiring individuals to be vaccinated to travel internationally? | 94.1% | 78.1% | 40.9% | 20.6% |
| Companies having the right to fire employees who refuse to get a coronavirus vaccine? | 64.4% | 36.2% | 13.4% | 9.8% |
| Companies having the right to require employees to be vaccinated before they can physically return to the workplace? | 89.3% | 64.2% | 23.6% | 12.3% |
| Health authorities making vaccinations mandatory to use trains and buses? | 87.3% | 64.2% | 25.7% | 12.3% |
| A government requirement for individuals to be vaccinated to enter [the respondent's country] from abroad? | 95.7% | 84.3% | 50.9% | 27.2% |
| Health authorities making vaccinations mandatory for everyone who can be safely vaccinated? | 89.0% | 66.5% | 23.7% | 14.6% |

**Table B2**: COVID-19 conspiracy theories by latent class and country (% probably or definitely true)

| Some people believe that [item]. Others do not believe this. What do you think? | Strongly Support | Support w/Concerns | Vaccine Hesitant | Anti-Vax |
| --- | --- | --- | --- | --- |
| **USA** |  |  |  |  |
| The coronavirus was accidentally released from a laboratory in China | 23.3% | 35.9% | 42.2% | 46.3% |
| The coronavirus was intentionally created as a plot to reduce the world’s population | 5.8% | 19.2% | 35.8% | 56.5% |
| 5G technology is causing the coronavirus to spread faster | 1.3% | 7.3% | 9.7% | 22.5% |
| Coronavirus is actually a biological weapon that was released from a laboratory in China | 14.3% | 30.4% | 48.8% | 58.2% |
| The coronavirus isn’t real, and doctors and scientists are in on the elaborate hoax | 11.2% | 9.4% | 9.6% | 23.1% |
|  |  |  |  |  |
| **UK** |  |  |  |  |
| The coronavirus was accidentally released from a laboratory in China | 23.9% | 36.3% | 39.4% | 49.1% |
| The coronavirus was intentionally created as a plot to reduce the world’s population | 5.0% | 15.1% | 37.5% | 55.0% |
| 5G technology is causing the coronavirus to spread faster | 0.8% | 5.2% | 15.2% | 19.0% |
| Coronavirus is actually a biological weapon that was released from a laboratory in China | 10.6% | 22.7% | 35.8% | 50.1% |
| The coronavirus isn’t real, and doctors and scientists are in on the elaborate hoax | 13.1% | 12.3% | 10.3% | 21.6% |
|  |  |  |  |  |
| **Canada** |  |  |  |  |
| The coronavirus was accidentally released from a laboratory in China | 17.9% | 27.6% | 36.9% | 42.8% |
| The coronavirus was intentionally created as a plot to reduce the world’s population | 3.4% | 13.2% | 32.0% | 53.1% |
| 5G technology is causing the coronavirus to spread faster | 1.0% | 6.1% | 9.2% | 17.3% |
| Coronavirus is actually a biological weapon that was released from a laboratory in China | 8.3% | 18.7% | 38.2% | 48.4% |
| The coronavirus isn’t real, and doctors and scientists are in on the elaborate hoax | 12.9% | 15.4% | 13.2% | 21.5% |

**Table B3**: Sample characteristics, weighted and unweighted, compared to poplation parameters (United States)

|  | Weighted (%) | Unweighted (%) | Population parameter (%) |
| --- | --- | --- | --- |
| *Sex* |  |  |  |
| Male | 47.3 | 47.8 | 48.7 |
| Female | 52.7 | 52.2 | 51.3 |
|  |  |  |  |
| *Age group* |  |  |  |
| 18-24 | 11.7 | 5.9 | 12.1 |
| 25-34 | 17.1 | 10.6 | 17.8 |
| 35-44 | 16.3 | 13.6 | 16.4 |
| 45-54 | 16.8 | 19.3 | 16.4 |
| 55-65 | 17.2 | 24.4 | 16.7 |
| 65+ | 20.8 | 26.2 | 20.6 |
|  |  |  |  |
| *Education* |  |  |  |
| High school or less | 36.2 | 13.2 | 39.3 |
| Some college | 32.0 | 29.4 | 30.6 |
| Bachelors degree | 19.9 | 30.7 | 18.9 |
| Graduate degree | 11.9 | 26.7 | 11.2 |
|  |  |  |  |
| Race |  |  |  |
| Non-Hispanic White | 65.7 | 76.5 | 63.1 |
| Non-Hispanic Black | 12.2 | 8.9 | 12.0 |
| Hispanic | 14.1 | 6.0 | 16.2 |
| Other | 8.1 | 8.7 | 8.6 |
|  |  |  |  |
| *Region* |  |  |  |
| Northeast | 17.4 | 15.4 | 17.5 |
| Midwest | 21.4 | 24.9 | 20.8 |
| South | 38.0 | 37.3 | 37.9 |
| West | 23.1 | 22.4 | 23.7 |

**Table B4**: Sample characteristics, weighted and unweighted, compared to population parameters (United Kingdom)

|  | Weighted (%) | Unweighted (%) | Population parameter (%) |
| --- | --- | --- | --- |
| *Sex* |  |  |  |
| Male | 48.4 | 47.3 | 48.9 |
| Female | 51.6 | 52.7 | 51.1 |
|  |  |  |  |
| *Age group* |  |  |  |
| 18-24 | 10.6 | 6.8 | 10.7 |
| 25-34 | 17.4 | 10.8 | 17.1 |
| 35-44 | 15.9 | 16.0 | 16.0 |
| 45-54 | 16.8 | 20.3 | 17.2 |
| 55-65 | 15.5 | 24.0 | 15.5 |
| 65+ | 23.8 | 22.1 | 23.5 |
|  |  |  |  |
| *Education** |  |  |  |
| Age 18-69: None | 10.8 | 3.0 | 13.7 |
| Age 18-69: O-level/GCSE | 16.1 | 12.0 | 15.4 |
| Age 18-69: A-level | 19.3 | 12.3 | 18.6 |
| Age 18-69: HND/HNC/trade | 7.5 | 17.1 | 7.2 |
| Age 18-69: Bachelors degree | 18.8 | 23.6 | 18.1 |
| Age 18-69: Postgraduate degree | 10.6 | 19.0 | 10.1 |
| Age 70+ | 16.9 | 13.1 | 16.8 |
|  |  |  |  |
| *Region* |  |  |  |
| North East | 3.7 | 4.0 | 4.1 |
| North West | 10.6 | 10.0 | 11.2 |
| Yorkshire and The Humber | 7.9 | 7.9 | 8.3 |
| East Midlands | 7.5 | 6.8 | 7.3 |
| West Midlands | 9.1 | 7.6 | 8.7 |
| East of England | 9.3 | 10.6 | 9.2 |
| London | 13.7 | 10.7 | 12.7 |
| South East | 13.5 | 16.7 | 13.7 |
| South West | 8.9 | 10.2 | 8.5 |
| Wales | 4.9 | 5.2 | 4.9 |
| Scotland | 8.5 | 8.7 | 8.6 |
| Northern Ireland | 2.3 | 1.6 | 2.8 |

**Table B5**: Sample characteristics, weighted and unweighted, compared to population parameters (Canada)

|  | Weighted (%) | Unweighted (%) | Population parameter (%) |
| --- | --- | --- | --- |
| *Sex* |  |  |  |
| Male | 50.0 | 52.8 | 49.4 |
| Female | 50.0 | 47.2 | 50.6 |
|  |  |  |  |
| *Age group* |  |  |  |
| 18-24 | 10.9 | 6.7 | 11.0 |
| 25-34 | 17.6 | 11.7 | 17.2 |
| 35-44 | 17.5 | 15.5 | 16.6 |
| 45-54 | 16.1 | 21.0 | 15.7 |
| 55-65 | 17.6 | 23.2 | 17.3 |
| 65+ | 20.3 | 21.9 | 22.2 |
|  |  |  |  |
| *Education* |  |  |  |
| High school or less | 36.4 | 18.5 | 38.0 |
| College/CEGEP | 33.5 | 35.6 | 32.8 |
| Bachelors degree | 20.4 | 26.8 | 19.8 |
| Graduate degree | 9.7 | 19.0 | 9.3 |
|  |  |  |  |
| *Region* |  |  |  |
| Atlantic | 6.5 | 5.7 | 6.6 |
| Quebec | 23.8 | 16.6 | 22.7 |
| Ontario | 38.6 | 38.8 | 39.0 |
| Manitoba & Saskatchewan | 6.4 | 7.5 | 6.4 |
| Alberta | 11.1 | 18.1 | 11.2 |
| British Columbia | 13.7 | 13.4 | 13.9 |
|  |  |  |  |
| *Language most often spoken in the home* |  |  |  |
| English | 63.3 | 75.0 | 63.2 |
| French | 20.2 | 13.4 | 20.3 |
| Other language | 16.5 | 11.5 | 16.5 |

**Table B6**: Overall fit of the latent class model.

| Log-likelihood Value | -104621.437 |
| --- | --- |
| Akaike Information Criteria | 209546.874 |
| Sample Size Adjusted Bayesian Information Criteria | 210202.591 |
| Entropy | 0.832 |
| Vuong-Lo-Mendell-Rubin LR Test for 4 vs. 5 classes | p=0.8310 |

Notes: Vuong-Lo-Mendell Test suggests that additional classes lead to loss of statistical efficiency and parsimony. A five-class model was selected to allow exposure of the “response set bias” class, that is the group of respondents who report strongly agreeing with all questions asked on the vaccine battery regardless of direction of the question. Entropy statistics suggest that there is an 83.2% probability of a respondent being correctly classified and this exceeds the suggested cut-off of 80%.

**Appendix C: Cross-National Latent Class Analysis (United States, United Kingdom, Canada)**

Mplus VERSION 8.5

MUTHEN & MUTHEN

06/15/2021 5:31 PM

INPUT INSTRUCTIONS

Title:

Stata2Mplus conversion for C:\Users\...\

List of variables converted shown below

RespondentID :

weight :

ProtChld : Getting vaccines is a good way to protect children from disease.

1: Strongly agree

2: Agree

3: Neither agree nor disagree

4: Disagree

5: Strongly disagree

DrRecVax : Generally, I do what my doctor recommends about vaccines.

1: Strongly agree

2: Agree

3: Neither agree nor disagree

4: Disagree

5: Strongly disagree

NewVaxSf : New vaccines are recommended only if they are safe.

1: Strongly agree

2: Agree

3: Neither agree nor disagree

4: Disagree

5: Strongly disagree

UnvxChld : Enough children are vaccinated that even unvaccinated children are safe from

1: Strongly agree

2: Agree

3: Neither agree nor disagree

4: Disagree

5: Strongly disagree

CncSdEff : I am concerned about serious side effects of vaccines.

1: Strongly agree

2: Agree

3: Neither agree nor disagree

4: Disagree

5: Strongly disagree

VaxAut : Some vaccines cause autism in healthy children.

1: Strongly agree

2: Agree

3: Neither agree nor disagree

4: Disagree

5: Strongly disagree

PrntRefu : Parents should have the right to refuse vaccines required for schools for any

1: Strongly agree

2: Agree

3: Neither agree nor disagree

4: Disagree

5: Strongly disagree

VaxAchv : Vaccinations are one of the most significant achievements in improving public

1: Strongly agree

2: Agree

3: Neither agree nor disagree

4: Disagree

5: Strongly disagree

group :

id :

Data:

File is C:\...\combinedformplus.dat ;

Variable:

Names are

RespondentID weight ProtChld DrRecVax NewVaxSf UnvxChld CncSdEff VaxAut

PrntRefu VaxAchv group id;

Missing are all (-9999) ;

Usevariables are ProtChld DrRecVax NewVaxSf VaxAchv

CncSdEff VaxAut PrntRefu canada uk;

Categorical are ProtChld DrRecVax NewVaxSf VaxAchv

CncSdEff VaxAut PrntRefu;

Weight=weight;

Idvariable =id;

classes= c(5);

Missing are all (-9999) ;

Define:

! us= (group==1);

canada= (group==1);

uk=(group==2);

Analysis:

Stseed=1234;

Type = mixture ;

Starts= 250 250;

Model:

%Overall%

c on canada uk;

Output:

Tech11;

Savedata:

File is probability.csv;

Save is CPROB;

*** WARNING

Input line exceeded 90 characters. Some input may be truncated.

Stata2Mplus conversion for C:\Users\...\a

*** WARNING

Input line exceeded 90 characters. Some input may be truncated.

UnvxChld : Enough children are vaccinated that even unvaccinated children are safe from d

*** WARNING

Input line exceeded 90 characters. Some input may be truncated.

VaxAchv : Vaccinations are one of the most significant achievements in improving public h

*** WARNING

Data set contains cases with missing on all variables except

x-variables. These cases were not included in the analysis.

Number of cases with missing on all variables except x-variables: 7

4 WARNING(S) FOUND IN THE INPUT INSTRUCTIONS

Stata2Mplus conversion for C:\Users\...\

List of variables converted shown below

RespondentID :

weight :

ProtChld : Getting vaccines is a good way to protect children from disease.

1: Strongly agree

2: Agree

3: Neither agree nor disagree

4: Disagree

5: Strongly disagree

DrRecVax : Generally, I do what my doctor recommends about vaccines.

1: Strongly agree

2: Agree

3: Neither agree nor disagree

4: Disagree

5: Strongly disagree

NewVaxSf : New vaccines are recommended only if they are safe.

1: Strongly agree

2: Agree

3: Neither agree nor disagree

4: Disagree

5: Strongly disagree

UnvxChld : Enough children are vaccinated that even unvaccinated children are safe from

1: Strongly agree

2: Agree

3: Neither agree nor disagree

4: Disagree

5: Strongly disagree

CncSdEff : I am concerned about serious side effects of vaccines.

1: Strongly agree

2: Agree

3: Neither agree nor disagree

4: Disagree

5: Strongly disagree

VaxAut : Some vaccines cause autism in healthy children.

1: Strongly agree

2: Agree

3: Neither agree nor disagree

4: Disagree

5: Strongly disagree

PrntRefu : Parents should have the right to refuse vaccines required for schools for any

1: Strongly agree

2: Agree

3: Neither agree nor disagree

4: Disagree

5: Strongly disagree

VaxAchv : Vaccinations are one of the most significant achievements in improving public

1: Strongly agree

2: Agree

3: Neither agree nor disagree

4: Disagree

5: Strongly disagree

group :

id :

SUMMARY OF ANALYSIS

Number of groups 1

Number of observations 13251

Number of dependent variables 7

Number of independent variables 2

Number of continuous latent variables 0

Number of categorical latent variables 1

Observed dependent variables

Binary and ordered categorical (ordinal)

PROTCHLD DRRECVAX NEWVAXSF VAXACHV CNCSDEFF VAXAUT

PRNTREFU

Observed independent variables

CANADA UK

Categorical latent variables

C

Variables with special functions

Weight variable WEIGHT

ID variable ID

Estimator MLR

Information matrix OBSERVED

Optimization Specifications for the Quasi-Newton Algorithm for

Continuous Outcomes

Maximum number of iterations 100

Convergence criterion 0.100D-05

Optimization Specifications for the EM Algorithm

Maximum number of iterations 500

Convergence criteria

Loglikelihood change 0.100D-06

Relative loglikelihood change 0.100D-06

Derivative 0.100D-05

Optimization Specifications for the M step of the EM Algorithm for

Categorical Latent variables

Number of M step iterations 1

M step convergence criterion 0.100D-05

Basis for M step termination ITERATION

Optimization Specifications for the M step of the EM Algorithm for

Censored, Binary or Ordered Categorical (Ordinal), Unordered

Categorical (Nominal) and Count Outcomes

Number of M step iterations 1

M step convergence criterion 0.100D-05

Basis for M step termination ITERATION

Maximum value for logit thresholds 15

Minimum value for logit thresholds -15

Minimum expected cell size for chi-square 0.100D-01

Maximum number of iterations for H1 2000

Convergence criterion for H1 0.100D-03

Optimization algorithm EMA

Random Starts Specifications

Number of initial stage random starts 250

Number of final stage optimizations 250

Number of initial stage iterations 10

Initial stage convergence criterion 0.100D+01

Random starts scale 0.500D+01

Random seed for generating random starts 1234

Link LOGIT

Input data file(s)

C:\...\

Input data format FREE

SUMMARY OF DATA

Number of missing data patterns 40

Number of y missing data patterns 0

Number of u missing data patterns 40

COVARIANCE COVERAGE OF DATA

Minimum covariance coverage value 0.100

PROPORTION OF DATA PRESENT FOR U

Covariance Coverage

PROTCHLD DRRECVAX NEWVAXSF VAXACHV CNCSDEFF

________ ________ ________ ________ ________

PROTCHLD 0.996

DRRECVAX 0.993 0.996

NEWVAXSF 0.993 0.993 0.996

VAXACHV 0.994 0.994 0.994 0.997

CNCSDEFF 0.993 0.993 0.992 0.994 0.996

VAXAUT 0.992 0.991 0.991 0.993 0.991

PRNTREFU 0.994 0.994 0.993 0.995 0.994

Covariance Coverage

VAXAUT PRNTREFU

________ ________

VAXAUT 0.994

PRNTREFU 0.992 0.997

UNIVARIATE PROPORTIONS AND COUNTS FOR CATEGORICAL VARIABLES

PROTCHLD

Category 1 0.031 411.682

Category 2 0.027 358.921

Category 3 0.107 1406.912

Category 4 0.292 3857.810

Category 5 0.543 7157.949

DRRECVAX

Category 1 0.033 436.025

Category 2 0.060 797.506

Category 3 0.148 1946.535

Category 4 0.358 4718.128

Category 5 0.401 5288.385

NEWVAXSF

Category 1 0.043 565.497

Category 2 0.064 847.896

Category 3 0.150 1979.933

Category 4 0.339 4468.869

Category 5 0.404 5323.601

VAXACHV

Category 1 0.034 453.336

Category 2 0.039 513.969

Category 3 0.133 1755.159

Category 4 0.298 3936.204

Category 5 0.496 6543.630

CNCSDEFF

Category 1 0.177 2330.591

Category 2 0.247 3260.763

Category 3 0.220 2897.102

Category 4 0.214 2817.354

Category 5 0.143 1884.727

VAXAUT

Category 1 0.059 772.538

Category 2 0.087 1151.271

Category 3 0.349 4588.775

Category 4 0.167 2204.844

Category 5 0.338 4446.043

PRNTREFU

Category 1 0.153 2022.124

Category 2 0.182 2407.533

Category 3 0.183 2412.112

Category 4 0.258 3407.671

Category 5 0.224 2950.861

UNIVARIATE SAMPLE STATISTICS

UNIVARIATE HIGHER-ORDER MOMENT DESCRIPTIVE STATISTICS

Variable/ Mean/ Skewness/ Minimum/ % with Percentiles

Sample Size Variance Kurtosis Maximum Min/Max 20%/60% 40%/80% Median

CANADA 0.309 0.828 0.000 69.14% 0.000 0.000 0.000

13251.000 0.213 -1.314 1.000 30.86% 0.000 1.000

UK 0.343 0.660 0.000 65.66% 0.000 0.000 0.000

13251.000 0.225 -1.564 1.000 34.34% 0.000 1.000

RANDOM STARTS RESULTS RANKED FROM THE BEST TO THE WORST LOGLIKELIHOOD VALUES

Final stage loglikelihood values at local maxima, seeds, and initial stage start numbers:

-104621.437 95220 111

-104621.437 418410 101

-104621.437 659136 207

-104621.437 494996 91

-104621.437 389320 106

-104621.437 79474 115

-104621.437 789192 172

-104621.437 147827 139

-104621.437 505902 140

-104621.437 551588 178

-104621.437 749092 177

-104621.437 559172 204

-104621.437 30702 168

-104621.437 36496 173

-104621.437 433735 40

-104621.437 864668 19

-104621.437 98826 88

-104621.437 545354 183

-104621.437 717812 190

-104621.437 377064 29

-104621.437 285532 120

-104621.437 283916 133

-104621.437 134946 209

-104621.437 45321 176

-104621.437 134751 220

-104621.437 605662 31

-104621.437 366399 13

-104621.437 702782 89

-104621.437 155075 6

-104621.437 107886 9

-104621.437 171477 224

-104621.437 528306 23

-104621.437 199792 146

-104621.437 54821 175

-104621.437 622917 144

-104621.437 307261 160

-104621.437 867074 17

-104621.437 715244 206

-104621.437 135227 18

-104621.437 956589 229

-104621.437 722706 195

-104621.437 606496 142

-104621.437 70428 93

-104621.437 404869 118

-104621.437 138304 65

-104621.437 3016 222

-104621.437 668446 119

-104752.164 731089 245

-104857.293 50821 199

-104857.293 170491 14

Unperturbed starting value run did not converge or was rejected in the third stage.

199 perturbed starting value run(s) did not converge or were rejected in the third stage.

THE BEST LOGLIKELIHOOD VALUE HAS BEEN REPLICATED. RERUN WITH AT LEAST TWICE THE

RANDOM STARTS TO CHECK THAT THE BEST LOGLIKELIHOOD IS STILL OBTAINED AND REPLICATED.

THE MODEL ESTIMATION TERMINATED NORMALLY

MODEL FIT INFORMATION

Number of Free Parameters 152

Loglikelihood

H0 Value -104621.437

H0 Scaling Correction Factor 1.8711

for MLR

Information Criteria

Akaike (AIC) 209546.874

Bayesian (BIC) 210685.632

Sample-Size Adjusted BIC 210202.591

(n* = (n + 2) / 24)

Chi-Square Test of Model Fit for the Binary and Ordered Categorical

(Ordinal) Outcomes**

Pearson Chi-Square

Value 42047.362

Degrees of Freedom 77733

P-Value 1.0000

Likelihood Ratio Chi-Square

Value 26323.850

Degrees of Freedom 77733

P-Value 1.0000

** Of the 238180 cells in the latent class indicator table, 247

were deleted in the calculation of chi-square due to extreme values.

Chi-Square Test for MCAR under the Unrestricted Latent Class Indicator Model

Pearson Chi-Square

Value 31634.902

Degrees of Freedom 160016

P-Value 1.0000

Likelihood Ratio Chi-Square

Value 29651.726

Degrees of Freedom 160016

P-Value 1.0000

FINAL CLASS COUNTS AND PROPORTIONS FOR THE LATENT CLASSES

BASED ON THE ESTIMATED MODEL

Latent

Classes

1 3710.89378 0.28005

2 742.91391 0.05606

3 5192.57526 0.39186

4 1410.00907 0.10641

5 2194.60798 0.16562

FINAL CLASS COUNTS AND PROPORTIONS FOR THE LATENT CLASSES

BASED ON ESTIMATED POSTERIOR PROBABILITIES

Latent

Classes

1 3710.89383 0.28005

2 742.91346 0.05606

3 5192.57499 0.39186

4 1410.00903 0.10641

5 2194.60870 0.16562

FINAL CLASS COUNTS AND PROPORTIONS FOR THE LATENT CLASSES

BASED ON THEIR MOST LIKELY LATENT CLASS MEMBERSHIP

Class Counts and Proportions

Latent

Classes

1 3773 0.28470

2 718 0.05416

3 5303 0.40019

4 1271 0.09595

5 2186 0.16499

CLASSIFICATION QUALITY

Entropy 0.832

Average Latent Class Probabilities for Most Likely Latent Class Membership (Row)

by Latent Class (Column)

1 2 3 4 5

1 0.876 0.000 0.043 0.030 0.051

2 0.001 0.923 0.001 0.009 0.066

3 0.031 0.001 0.927 0.042 0.000

4 0.069 0.005 0.090 0.817 0.019

5 0.071 0.032 0.000 0.014 0.883

Classification Probabilities for the Most Likely Latent Class Membership (Column)

by Latent Class (Row)

1 2 3 4 5

1 0.891 0.000 0.044 0.023 0.042

2 0.002 0.892 0.004 0.009 0.093

3 0.031 0.000 0.946 0.022 0.000

4 0.079 0.005 0.157 0.737 0.022

5 0.087 0.022 0.000 0.011 0.880

Logits for the Classification Probabilities for the Most Likely Latent Class Membership (Column)

by Latent Class (Row)

1 2 3 4 5

1 3.062 -5.704 0.058 -0.573 0.000

2 -3.944 2.262 -3.084 -2.317 0.000

3 6.103 0.801 9.509 5.750 0.000

4 1.268 -1.582 1.954 3.498 0.000

5 -2.310 -3.709 -8.618 -4.370 0.000

MODEL RESULTS

Two-Tailed

Estimate S.E. Est./S.E. P-Value

Latent Class 1

Thresholds

PROTCHLD$1 -7.295 1.003 -7.276 0.000

PROTCHLD$2 -5.253 0.450 -11.667 0.000

PROTCHLD$3 -3.497 0.213 -16.443 0.000

PROTCHLD$4 1.016 0.112 9.107 0.000

DRRECVAX$1 -7.877 1.344 -5.861 0.000

DRRECVAX$2 -3.548 0.177 -20.011 0.000

DRRECVAX$3 -1.690 0.085 -19.954 0.000

DRRECVAX$4 2.208 0.132 16.775 0.000

NEWVAXSF$1 -5.830 0.506 -11.512 0.000

NEWVAXSF$2 -2.918 0.136 -21.426 0.000

NEWVAXSF$3 -1.229 0.086 -14.229 0.000

NEWVAXSF$4 1.803 0.090 20.136 0.000

VAXACHV$1 -7.477 0.734 -10.183 0.000

VAXACHV$2 -4.289 0.258 -16.642 0.000

VAXACHV$3 -2.344 0.139 -16.876 0.000

VAXACHV$4 1.461 0.118 12.379 0.000

CNCSDEFF$1 -2.659 0.158 -16.799 0.000

CNCSDEFF$2 -0.180 0.077 -2.325 0.020

CNCSDEFF$3 1.191 0.081 14.658 0.000

CNCSDEFF$4 4.002 0.211 18.930 0.000

VAXAUT$1 -5.854 0.650 -9.005 0.000

VAXAUT$2 -2.244 0.116 -19.422 0.000

VAXAUT$3 0.292 0.075 3.907 0.000

VAXAUT$4 1.804 0.090 20.043 0.000

PRNTREFU$1 -3.229 0.200 -16.119 0.000

PRNTREFU$2 -0.713 0.073 -9.731 0.000

PRNTREFU$3 0.306 0.074 4.116 0.000

PRNTREFU$4 2.756 0.127 21.734 0.000

Latent Class 2

Thresholds

PROTCHLD$1 -0.078 0.252 -0.311 0.756

PROTCHLD$2 0.769 0.273 2.821 0.005

PROTCHLD$3 1.993 0.297 6.716 0.000

PROTCHLD$4 2.781 0.284 9.800 0.000

DRRECVAX$1 -0.129 0.225 -0.575 0.565

DRRECVAX$2 1.041 0.223 4.674 0.000

DRRECVAX$3 2.354 0.301 7.830 0.000

DRRECVAX$4 3.117 0.340 9.158 0.000

NEWVAXSF$1 0.084 0.174 0.484 0.628

NEWVAXSF$2 0.716 0.140 5.122 0.000

NEWVAXSF$3 1.429 0.180 7.944 0.000

NEWVAXSF$4 1.674 0.193 8.659 0.000

VAXACHV$1 0.146 0.259 0.561 0.575

VAXACHV$2 1.081 0.280 3.863 0.000

VAXACHV$3 2.387 0.323 7.400 0.000

VAXACHV$4 3.812 0.427 8.924 0.000

CNCSDEFF$1 1.289 0.151 8.543 0.000

CNCSDEFF$2 1.663 0.193 8.599 0.000

CNCSDEFF$3 1.993 0.214 9.308 0.000

CNCSDEFF$4 2.523 0.258 9.786 0.000

VAXAUT$1 -0.024 0.138 -0.175 0.861

VAXAUT$2 0.577 0.125 4.616 0.000

VAXAUT$3 1.710 0.174 9.800 0.000

VAXAUT$4 2.071 0.219 9.458 0.000

PRNTREFU$1 1.403 0.145 9.675 0.000

PRNTREFU$2 1.801 0.179 10.054 0.000

PRNTREFU$3 2.079 0.214 9.736 0.000

PRNTREFU$4 2.482 0.261 9.493 0.000

Latent Class 3

Thresholds

PROTCHLD$1 -5.145 0.218 -23.603 0.000

PROTCHLD$2 -4.809 0.200 -24.093 0.000

PROTCHLD$3 -4.349 0.159 -27.270 0.000

PROTCHLD$4 -2.935 0.103 -28.563 0.000

DRRECVAX$1 -5.547 0.230 -24.137 0.000

DRRECVAX$2 -4.684 0.184 -25.434 0.000

DRRECVAX$3 -3.053 0.093 -32.904 0.000

DRRECVAX$4 -1.123 0.051 -21.921 0.000

NEWVAXSF$1 -4.788 0.216 -22.197 0.000

NEWVAXSF$2 -4.014 0.153 -26.189 0.000

NEWVAXSF$3 -2.799 0.090 -31.076 0.000

NEWVAXSF$4 -0.636 0.045 -14.144 0.000

VAXACHV$1 -5.159 0.268 -19.220 0.000

VAXACHV$2 -4.919 0.237 -20.742 0.000

VAXACHV$3 -4.131 0.161 -25.677 0.000

VAXACHV$4 -2.172 0.076 -28.722 0.000

CNCSDEFF$1 -4.078 0.208 -19.633 0.000

CNCSDEFF$2 -1.920 0.085 -22.622 0.000

CNCSDEFF$3 -0.819 0.073 -11.230 0.000

CNCSDEFF$4 0.683 0.059 11.628 0.000

VAXAUT$1 -6.096 0.471 -12.951 0.000

VAXAUT$2 -5.139 0.439 -11.712 0.000

VAXAUT$3 -1.968 0.153 -12.888 0.000

VAXAUT$4 -0.895 0.095 -9.449 0.000

PRNTREFU$1 -4.189 0.174 -24.127 0.000

PRNTREFU$2 -2.538 0.101 -25.089 0.000

PRNTREFU$3 -1.579 0.087 -18.145 0.000

PRNTREFU$4 0.028 0.056 0.493 0.622

Latent Class 4

Thresholds

PROTCHLD$1 -5.770 0.937 -6.156 0.000

PROTCHLD$2 -4.819 0.509 -9.471 0.000

PROTCHLD$3 -3.174 0.310 -10.246 0.000

PROTCHLD$4 -1.640 0.197 -8.337 0.000

DRRECVAX$1 -3.890 0.380 -10.231 0.000

DRRECVAX$2 -3.165 0.279 -11.361 0.000

DRRECVAX$3 -1.800 0.160 -11.248 0.000

DRRECVAX$4 -0.635 0.119 -5.315 0.000

NEWVAXSF$1 -4.116 0.450 -9.155 0.000

NEWVAXSF$2 -3.131 0.285 -10.978 0.000

NEWVAXSF$3 -1.782 0.167 -10.705 0.000

NEWVAXSF$4 -0.927 0.151 -6.141 0.000

VAXACHV$1 -6.467 1.236 -5.234 0.000

VAXACHV$2 -5.717 1.520 -3.761 0.000

VAXACHV$3 -2.650 0.291 -9.102 0.000

VAXACHV$4 -1.460 0.199 -7.341 0.000

CNCSDEFF$1 -0.323 0.266 -1.214 0.225

CNCSDEFF$2 0.703 0.250 2.810 0.005

CNCSDEFF$3 2.347 0.391 6.010 0.000

CNCSDEFF$4 4.115 0.506 8.129 0.000

VAXAUT$1 -1.678 0.244 -6.881 0.000

VAXAUT$2 -1.034 0.206 -5.024 0.000

VAXAUT$3 1.482 0.228 6.492 0.000

VAXAUT$4 2.234 0.229 9.737 0.000

PRNTREFU$1 -0.530 0.257 -2.067 0.039

PRNTREFU$2 0.151 0.212 0.709 0.478

PRNTREFU$3 1.330 0.266 5.003 0.000

PRNTREFU$4 2.568 0.269 9.563 0.000

Latent Class 5

Thresholds

PROTCHLD$1 -4.711 0.429 -10.992 0.000

PROTCHLD$2 -2.336 0.184 -12.676 0.000

PROTCHLD$3 0.374 0.148 2.536 0.011

PROTCHLD$4 3.798 0.383 9.928 0.000

DRRECVAX$1 -3.956 0.312 -12.682 0.000

DRRECVAX$2 -1.264 0.130 -9.694 0.000

DRRECVAX$3 0.787 0.141 5.574 0.000

DRRECVAX$4 3.375 0.253 13.363 0.000

NEWVAXSF$1 -2.981 0.259 -11.499 0.000

NEWVAXSF$2 -1.020 0.119 -8.577 0.000

NEWVAXSF$3 0.711 0.091 7.851 0.000

NEWVAXSF$4 1.846 0.124 14.847 0.000

VAXACHV$1 -4.580 0.466 -9.838 0.000

VAXACHV$2 -1.755 0.153 -11.488 0.000

VAXACHV$3 0.884 0.154 5.746 0.000

VAXACHV$4 3.897 0.333 11.690 0.000

CNCSDEFF$1 -0.483 0.133 -3.625 0.000

CNCSDEFF$2 1.227 0.104 11.750 0.000

CNCSDEFF$3 3.932 0.282 13.934 0.000

CNCSDEFF$4 6.009 0.658 9.130 0.000

VAXAUT$1 -2.496 0.220 -11.334 0.000

VAXAUT$2 -0.750 0.103 -7.291 0.000

VAXAUT$3 2.397 0.151 15.852 0.000

VAXAUT$4 3.762 0.274 13.711 0.000

PRNTREFU$1 -0.775 0.144 -5.366 0.000

PRNTREFU$2 0.666 0.103 6.474 0.000

PRNTREFU$3 2.797 0.192 14.576 0.000

PRNTREFU$4 4.629 0.386 12.000 0.000

Categorical Latent Variables

C#1 ON

CANADA 0.461 0.106 4.360 0.000

UK 0.933 0.112 8.331 0.000

C#2 ON

CANADA 0.349 0.153 2.278 0.023

UK 0.010 0.178 0.055 0.956

C#3 ON

CANADA 0.575 0.091 6.325 0.000

UK 1.070 0.102 10.505 0.000

C#4 ON

CANADA 0.359 0.148 2.425 0.015

UK 0.948 0.190 4.978 0.000

Intercepts

C#1 0.114 0.096 1.189 0.235

C#2 -1.200 0.152 -7.901 0.000

C#3 0.363 0.079 4.578 0.000

C#4 -0.829 0.126 -6.569 0.000

RESULTS IN PROBABILITY SCALE

Latent Class 1

PROTCHLD

Category 1 0.001 0.001 0.998 0.318

Category 2 0.005 0.002 2.035 0.042

Category 3 0.024 0.006 4.370 0.000

Category 4 0.705 0.020 35.803 0.000

Category 5 0.266 0.022 12.208 0.000

DRRECVAX

Category 1 0.000 0.001 0.744 0.457

Category 2 0.028 0.005 5.805 0.000

Category 3 0.128 0.009 13.492 0.000

Category 4 0.745 0.012 59.728 0.000

Category 5 0.099 0.012 8.434 0.000

NEWVAXSF

Category 1 0.003 0.001 1.980 0.048

Category 2 0.048 0.006 7.666 0.000

Category 3 0.175 0.012 14.665 0.000

Category 4 0.632 0.016 39.570 0.000

Category 5 0.141 0.011 13.005 0.000

VAXACHV

Category 1 0.001 0.000 1.363 0.173

Category 2 0.013 0.003 3.805 0.000

Category 3 0.074 0.010 7.365 0.000

Category 4 0.724 0.015 47.786 0.000

Category 5 0.188 0.018 10.441 0.000

CNCSDEFF

Category 1 0.065 0.010 6.759 0.000

Category 2 0.390 0.014 27.112 0.000

Category 3 0.312 0.012 26.127 0.000

Category 4 0.215 0.013 16.343 0.000

Category 5 0.018 0.004 4.817 0.000

VAXAUT

Category 1 0.003 0.002 1.543 0.123

Category 2 0.093 0.009 9.877 0.000

Category 3 0.477 0.015 32.813 0.000

Category 4 0.286 0.013 22.655 0.000

Category 5 0.141 0.011 12.939 0.000

PRNTREFU

Category 1 0.038 0.007 5.190 0.000

Category 2 0.291 0.013 22.144 0.000

Category 3 0.247 0.011 22.960 0.000

Category 4 0.364 0.015 24.074 0.000

Category 5 0.060 0.007 8.386 0.000

Latent Class 2

PROTCHLD

Category 1 0.480 0.063 7.635 0.000

Category 2 0.203 0.024 8.510 0.000

Category 3 0.197 0.040 4.945 0.000

Category 4 0.062 0.025 2.440 0.015

Category 5 0.058 0.016 3.742 0.000

DRRECVAX

Category 1 0.468 0.056 8.345 0.000

Category 2 0.271 0.031 8.661 0.000

Category 3 0.174 0.031 5.596 0.000

Category 4 0.044 0.017 2.537 0.011

Category 5 0.042 0.014 3.068 0.002

NEWVAXSF

Category 1 0.521 0.043 12.032 0.000

Category 2 0.151 0.029 5.163 0.000

Category 3 0.135 0.020 6.790 0.000

Category 4 0.035 0.010 3.498 0.000

Category 5 0.158 0.026 6.140 0.000

VAXACHV

Category 1 0.536 0.065 8.312 0.000

Category 2 0.210 0.027 7.780 0.000

Category 3 0.169 0.040 4.242 0.000

Category 4 0.063 0.023 2.758 0.006

Category 5 0.022 0.009 2.392 0.017

CNCSDEFF

Category 1 0.784 0.026 30.679 0.000

Category 2 0.057 0.016 3.551 0.000

Category 3 0.039 0.011 3.550 0.000

Category 4 0.046 0.011 4.218 0.000

Category 5 0.074 0.018 4.191 0.000

VAXAUT

Category 1 0.494 0.035 14.279 0.000

Category 2 0.146 0.024 6.141 0.000

Category 3 0.206 0.027 7.588 0.000

Category 4 0.041 0.012 3.526 0.000

Category 5 0.112 0.022 5.144 0.000

PRNTREFU

Category 1 0.803 0.023 34.944 0.000

Category 2 0.056 0.017 3.278 0.001

Category 3 0.031 0.008 3.754 0.000

Category 4 0.034 0.009 3.941 0.000

Category 5 0.077 0.019 4.144 0.000

Latent Class 3

PROTCHLD

Category 1 0.006 0.001 4.615 0.000

Category 2 0.002 0.001 2.264 0.024

Category 3 0.005 0.001 3.866 0.000

Category 4 0.038 0.005 8.342 0.000

Category 5 0.950 0.005 192.932 0.000

DRRECVAX

Category 1 0.004 0.001 4.368 0.000

Category 2 0.005 0.001 3.683 0.000

Category 3 0.036 0.004 10.022 0.000

Category 4 0.200 0.009 23.315 0.000

Category 5 0.755 0.009 79.529 0.000

NEWVAXSF

Category 1 0.008 0.002 4.675 0.000

Category 2 0.009 0.002 4.740 0.000

Category 3 0.040 0.004 10.042 0.000

Category 4 0.289 0.009 31.039 0.000

Category 5 0.654 0.010 64.270 0.000

VAXACHV

Category 1 0.006 0.002 3.747 0.000

Category 2 0.002 0.001 1.990 0.047

Category 3 0.009 0.002 4.757 0.000

Category 4 0.086 0.006 13.390 0.000

Category 5 0.898 0.007 129.290 0.000

CNCSDEFF

Category 1 0.017 0.003 4.897 0.000

Category 2 0.111 0.008 13.770 0.000

Category 3 0.178 0.010 18.262 0.000

Category 4 0.358 0.009 40.013 0.000

Category 5 0.336 0.013 25.641 0.000

VAXAUT

Category 1 0.002 0.001 2.129 0.033

Category 2 0.004 0.002 1.712 0.087

Category 3 0.117 0.015 7.841 0.000

Category 4 0.167 0.008 21.587 0.000

Category 5 0.710 0.020 36.396 0.000

PRNTREFU

Category 1 0.015 0.003 5.847 0.000

Category 2 0.058 0.006 9.914 0.000

Category 3 0.098 0.008 12.367 0.000

Category 4 0.336 0.009 38.585 0.000

Category 5 0.493 0.014 35.103 0.000

Latent Class 4

PROTCHLD

Category 1 0.003 0.003 1.070 0.284

Category 2 0.005 0.003 1.806 0.071

Category 3 0.032 0.011 3.011 0.003

Category 4 0.122 0.021 5.830 0.000

Category 5 0.838 0.027 31.291 0.000

DRRECVAX

Category 1 0.020 0.007 2.684 0.007

Category 2 0.020 0.008 2.724 0.006

Category 3 0.101 0.015 6.890 0.000

Category 4 0.205 0.024 8.548 0.000

Category 5 0.654 0.027 24.173 0.000

NEWVAXSF

Category 1 0.016 0.007 2.261 0.024

Category 2 0.026 0.008 3.108 0.002

Category 3 0.102 0.016 6.393 0.000

Category 4 0.139 0.028 5.061 0.000

Category 5 0.717 0.031 23.362 0.000

VAXACHV

Category 1 0.002 0.002 0.811 0.418

Category 2 0.002 0.004 0.388 0.698

Category 3 0.063 0.016 3.889 0.000

Category 4 0.122 0.022 5.531 0.000

Category 5 0.812 0.030 26.681 0.000

CNCSDEFF

Category 1 0.420 0.065 6.475 0.000

Category 2 0.249 0.025 9.803 0.000

Category 3 0.244 0.032 7.646 0.000

Category 4 0.071 0.028 2.510 0.012

Category 5 0.016 0.008 2.008 0.045

VAXAUT

Category 1 0.157 0.032 4.865 0.000

Category 2 0.105 0.016 6.753 0.000

Category 3 0.552 0.027 20.420 0.000

Category 4 0.088 0.026 3.433 0.001

Category 5 0.097 0.020 4.824 0.000

PRNTREFU

Category 1 0.370 0.060 6.190 0.000

Category 2 0.167 0.020 8.174 0.000

Category 3 0.253 0.022 11.562 0.000

Category 4 0.138 0.034 4.107 0.000

Category 5 0.071 0.018 4.010 0.000

Latent Class 5

PROTCHLD

Category 1 0.009 0.004 2.354 0.019

Category 2 0.079 0.013 6.039 0.000

Category 3 0.504 0.028 17.743 0.000

Category 4 0.386 0.036 10.788 0.000

Category 5 0.022 0.008 2.673 0.008

DRRECVAX

Category 1 0.019 0.006 3.267 0.001

Category 2 0.201 0.020 10.242 0.000

Category 3 0.467 0.021 22.009 0.000

Category 4 0.280 0.030 9.288 0.000

Category 5 0.033 0.008 4.094 0.000

NEWVAXSF

Category 1 0.048 0.012 4.053 0.000

Category 2 0.217 0.016 13.360 0.000

Category 3 0.406 0.021 19.282 0.000

Category 4 0.193 0.019 10.002 0.000

Category 5 0.136 0.015 9.314 0.000

VAXACHV

Category 1 0.010 0.005 2.170 0.030

Category 2 0.137 0.017 8.241 0.000

Category 3 0.560 0.024 22.932 0.000

Category 4 0.273 0.030 8.938 0.000

Category 5 0.020 0.007 3.061 0.002

CNCSDEFF

Category 1 0.382 0.031 12.136 0.000

Category 2 0.392 0.023 16.676 0.000

Category 3 0.208 0.018 11.709 0.000

Category 4 0.017 0.005 3.291 0.001

Category 5 0.002 0.002 1.523 0.128

VAXAUT

Category 1 0.076 0.015 4.916 0.000

Category 2 0.245 0.015 16.610 0.000

Category 3 0.596 0.022 27.232 0.000

Category 4 0.061 0.009 6.481 0.000

Category 5 0.023 0.006 3.730 0.000

PRNTREFU

Category 1 0.315 0.031 10.114 0.000

Category 2 0.345 0.019 17.887 0.000

Category 3 0.282 0.020 13.892 0.000

Category 4 0.048 0.010 4.970 0.000

Category 5 0.010 0.004 2.618 0.009

LATENT CLASS ODDS RATIO RESULTS

95% C.I.

Estimate S.E. Lower 2.5% Upper 2.5%

Latent Class 1 Compared to Latent Class 2

PROTCHLD

Category > 1 1362.374 1418.683 176.969 10488.071

Category > 2 412.473 216.356 147.538 1153.152

Category > 3 242.283 81.034 125.784 466.681

Category > 4 5.843 1.780 3.216 10.616

DRRECVAX

Category > 1 2314.905 3127.948 163.814 32712.656

Category > 2 98.468 26.662 57.918 167.410

Category > 3 57.057 17.343 31.447 103.523

Category > 4 2.484 0.915 1.207 5.112

NEWVAXSF

Category > 1 370.173 199.088 129.003 1062.210

Category > 2 37.856 7.027 26.310 54.467

Category > 3 14.259 2.631 9.931 20.473

Category > 4 0.879 0.185 0.581 1.329

VAXACHV

Category > 1 2043.279 1621.701 431.258 9680.952

Category > 2 214.962 79.419 104.202 443.455

Category > 3 113.428 38.101 58.721 219.101

Category > 4 10.501 4.786 4.298 25.655

CNCSDEFF

Category > 1 51.839 11.726 33.275 80.761

Category > 2 6.315 1.408 4.079 9.776

Category > 3 2.229 0.551 1.372 3.619

Category > 4 0.228 0.080 0.114 0.454

VAXAUT

Category > 1 340.300 222.074 94.705 1222.793

Category > 2 16.797 2.788 12.133 23.254

Category > 3 4.127 0.836 2.774 6.139

Category > 4 1.306 0.331 0.795 2.144

PRNTREFU

Category > 1 102.669 25.472 63.132 166.964

Category > 2 12.356 2.521 8.283 18.431

Category > 3 5.888 1.428 3.660 9.471

Category > 4 0.760 0.233 0.417 1.386

Latent Class 1 Compared to Latent Class 3

PROTCHLD

Category > 1 8.591 9.050 1.090 67.732

Category > 2 1.558 0.812 0.561 4.328

Category > 3 0.426 0.118 0.248 0.734

Category > 4 0.019 0.002 0.015 0.025

DRRECVAX

Category > 1 10.274 14.034 0.706 149.444

Category > 2 0.321 0.085 0.191 0.540

Category > 3 0.256 0.032 0.200 0.327

Category > 4 0.036 0.005 0.028 0.046

NEWVAXSF

Category > 1 2.835 1.595 0.941 8.538

Category > 2 0.334 0.069 0.223 0.502

Category > 3 0.208 0.025 0.164 0.264

Category > 4 0.087 0.008 0.072 0.105

VAXACHV

Category > 1 10.154 8.229 2.074 49.710

Category > 2 0.533 0.195 0.260 1.092

Category > 3 0.167 0.036 0.110 0.256

Category > 4 0.026 0.003 0.021 0.033

CNCSDEFF

Category > 1 0.242 0.062 0.147 0.399

Category > 2 0.175 0.019 0.142 0.216

Category > 3 0.134 0.013 0.111 0.162

Category > 4 0.036 0.008 0.024 0.055

VAXAUT

Category > 1 0.785 0.630 0.163 3.789

Category > 2 0.055 0.025 0.023 0.133

Category > 3 0.104 0.016 0.077 0.141

Category > 4 0.067 0.008 0.054 0.084

PRNTREFU

Category > 1 0.383 0.100 0.230 0.638

Category > 2 0.161 0.019 0.128 0.203

Category > 3 0.152 0.016 0.124 0.186

Category > 4 0.065 0.008 0.051 0.084

Latent Class 1 Compared to Latent Class 4

PROTCHLD

Category > 1 4.596 6.503 0.287 73.586

Category > 2 1.542 1.069 0.397 5.998

Category > 3 1.380 0.530 0.651 2.928

Category > 4 0.070 0.016 0.045 0.110

DRRECVAX

Category > 1 53.847 75.612 3.435 844.181

Category > 2 1.468 0.500 0.753 2.862

Category > 3 0.896 0.165 0.624 1.287

Category > 4 0.058 0.010 0.042 0.081

NEWVAXSF

Category > 1 5.552 4.037 1.335 23.092

Category > 2 0.808 0.270 0.420 1.556

Category > 3 0.575 0.113 0.391 0.845

Category > 4 0.065 0.010 0.048 0.088

VAXACHV

Category > 1 2.744 4.021 0.155 48.497

Category > 2 0.240 0.377 0.011 5.226

Category > 3 0.736 0.244 0.384 1.411

Category > 4 0.054 0.013 0.034 0.086

CNCSDEFF

Category > 1 10.340 3.078 5.769 18.533

Category > 2 2.418 0.630 1.452 4.029

Category > 3 3.175 1.248 1.470 6.861

Category > 4 1.120 0.622 0.378 3.324

VAXAUT

Category > 1 65.085 43.842 17.382 243.707

Category > 2 3.356 0.782 2.126 5.297

Category > 3 3.285 0.763 2.083 5.178

Category > 4 1.538 0.380 0.947 2.496

PRNTREFU

Category > 1 14.855 4.562 8.137 27.121

Category > 2 2.371 0.526 1.535 3.662

Category > 3 2.785 0.748 1.644 4.716

Category > 4 0.828 0.245 0.464 1.480

Latent Class 1 Compared to Latent Class 5

PROTCHLD

Category > 1 13.257 14.865 1.472 119.386

Category > 2 18.485 9.209 6.962 49.079

Category > 3 47.988 9.063 33.142 69.485

Category > 4 16.147 6.628 7.223 36.098

DRRECVAX

Category > 1 50.405 70.519 3.248 782.275

Category > 2 9.814 2.037 6.533 14.741

Category > 3 11.916 1.542 9.247 15.355

Category > 4 3.215 0.957 1.794 5.760

NEWVAXSF

Category > 1 17.268 10.025 5.534 53.877

Category > 2 6.673 1.106 4.822 9.234

Category > 3 6.960 0.720 5.684 8.524

Category > 4 1.043 0.174 0.752 1.447

VAXACHV

Category > 1 18.119 16.635 2.996 109.561

Category > 2 12.603 3.774 7.007 22.668

Category > 3 25.225 3.501 19.218 33.110

Category > 4 11.429 4.027 5.728 22.801

CNCSDEFF

Category > 1 8.814 1.610 6.161 12.609

Category > 2 4.082 0.507 3.200 5.208

Category > 3 15.500 4.477 8.799 27.303

Category > 4 7.447 5.352 1.820 30.464

VAXAUT

Category > 1 28.741 19.312 7.701 107.268

Category > 2 4.455 0.625 3.384 5.865

Category > 3 8.203 1.247 6.090 11.049

Category > 4 7.083 2.039 4.029 12.452

PRNTREFU

Category > 1 11.630 2.569 7.542 17.932

Category > 2 3.970 0.451 3.178 4.960

Category > 3 12.069 2.181 8.469 17.200

Category > 4 6.505 2.688 2.894 14.621

Latent Class 2 Compared to Latent Class 3

PROTCHLD

Category > 1 0.006 0.002 0.003 0.012

Category > 2 0.004 0.001 0.002 0.007

Category > 3 0.002 0.001 0.001 0.003

Category > 4 0.003 0.001 0.002 0.006

DRRECVAX

Category > 1 0.004 0.001 0.002 0.008

Category > 2 0.003 0.001 0.002 0.006

Category > 3 0.004 0.001 0.002 0.008

Category > 4 0.014 0.005 0.007 0.028

NEWVAXSF

Category > 1 0.008 0.002 0.004 0.013

Category > 2 0.009 0.002 0.006 0.013

Category > 3 0.015 0.003 0.010 0.022

Category > 4 0.099 0.019 0.068 0.146

VAXACHV

Category > 1 0.005 0.002 0.002 0.011

Category > 2 0.002 0.001 0.001 0.005

Category > 3 0.001 0.001 0.001 0.003

Category > 4 0.003 0.001 0.001 0.006

CNCSDEFF

Category > 1 0.005 0.001 0.003 0.008

Category > 2 0.028 0.006 0.018 0.042

Category > 3 0.060 0.014 0.038 0.095

Category > 4 0.159 0.043 0.094 0.269

VAXAUT

Category > 1 0.002 0.001 0.001 0.006

Category > 2 0.003 0.001 0.001 0.008

Category > 3 0.025 0.006 0.016 0.040

Category > 4 0.052 0.013 0.032 0.083

PRNTREFU

Category > 1 0.004 0.001 0.002 0.006

Category > 2 0.013 0.003 0.009 0.020

Category > 3 0.026 0.006 0.016 0.041

Category > 4 0.086 0.023 0.051 0.146

Latent Class 2 Compared to Latent Class 4

PROTCHLD

Category > 1 0.003 0.003 0.001 0.022

Category > 2 0.004 0.002 0.001 0.011

Category > 3 0.006 0.002 0.003 0.013

Category > 4 0.012 0.004 0.006 0.023

DRRECVAX

Category > 1 0.023 0.010 0.010 0.054

Category > 2 0.015 0.005 0.008 0.029

Category > 3 0.016 0.005 0.008 0.030

Category > 4 0.023 0.008 0.012 0.047

NEWVAXSF

Category > 1 0.015 0.007 0.006 0.038

Category > 2 0.021 0.007 0.012 0.039

Category > 3 0.040 0.010 0.025 0.065

Category > 4 0.074 0.019 0.045 0.121

VAXACHV

Category > 1 0.001 0.002 0.000 0.016

Category > 2 0.001 0.002 0.000 0.022

Category > 3 0.006 0.003 0.003 0.015

Category > 4 0.005 0.002 0.002 0.012

CNCSDEFF

Category > 1 0.199 0.062 0.109 0.366

Category > 2 0.383 0.122 0.205 0.717

Category > 3 1.425 0.639 0.592 3.429

Category > 4 4.916 2.813 1.602 15.088

VAXAUT

Category > 1 0.191 0.053 0.111 0.331

Category > 2 0.200 0.048 0.125 0.319

Category > 3 0.796 0.230 0.452 1.401

Category > 4 1.178 0.377 0.629 2.206

PRNTREFU

Category > 1 0.145 0.043 0.081 0.258

Category > 2 0.192 0.054 0.111 0.331

Category > 3 0.473 0.163 0.241 0.928

Category > 4 1.089 0.416 0.515 2.305

Latent Class 2 Compared to Latent Class 5

PROTCHLD

Category > 1 0.010 0.004 0.005 0.021

Category > 2 0.045 0.011 0.028 0.072

Category > 3 0.198 0.061 0.108 0.362

Category > 4 2.764 1.394 1.029 7.425

DRRECVAX

Category > 1 0.022 0.006 0.013 0.038

Category > 2 0.100 0.020 0.067 0.148

Category > 3 0.209 0.067 0.111 0.392

Category > 4 1.294 0.568 0.547 3.060

NEWVAXSF

Category > 1 0.047 0.010 0.031 0.071

Category > 2 0.176 0.028 0.129 0.241

Category > 3 0.488 0.097 0.330 0.721

Category > 4 1.187 0.306 0.716 1.968

VAXACHV

Category > 1 0.009 0.003 0.004 0.019

Category > 2 0.059 0.014 0.037 0.094

Category > 3 0.222 0.079 0.111 0.446

Category > 4 1.088 0.590 0.376 3.151

CNCSDEFF

Category > 1 0.170 0.040 0.108 0.269

Category > 2 0.646 0.164 0.393 1.063

Category > 3 6.955 2.583 3.359 14.403

Category > 4 32.680 22.610 8.421 126.823

VAXAUT

Category > 1 0.084 0.019 0.055 0.131

Category > 2 0.265 0.043 0.193 0.364

Category > 3 1.988 0.499 1.215 3.253

Category > 4 5.426 1.968 2.665 11.044

PRNTREFU

Category > 1 0.113 0.024 0.074 0.172

Category > 2 0.321 0.076 0.202 0.512

Category > 3 2.050 0.659 1.091 3.851

Category > 4 8.556 4.035 3.395 21.562

Latent Class 3 Compared to Latent Class 4

PROTCHLD

Category > 1 0.535 0.528 0.077 3.705

Category > 2 0.990 0.557 0.329 2.982

Category > 3 3.238 1.164 1.601 6.548

Category > 4 3.652 0.811 2.364 5.643

DRRECVAX

Category > 1 5.241 2.462 2.087 13.163

Category > 2 4.569 1.568 2.332 8.951

Category > 3 3.502 0.649 2.436 5.035

Category > 4 1.629 0.212 1.262 2.104

NEWVAXSF

Category > 1 1.958 1.028 0.700 5.481

Category > 2 2.417 0.848 1.215 4.808

Category > 3 2.763 0.555 1.864 4.095

Category > 4 0.747 0.119 0.547 1.020

VAXACHV

Category > 1 0.270 0.354 0.021 3.516

Category > 2 0.450 0.707 0.021 9.760

Category > 3 4.397 1.533 2.221 8.707

Category > 4 2.038 0.441 1.333 3.115

CNCSDEFF

Category > 1 42.709 12.167 24.435 74.647

Category > 2 13.780 3.071 8.904 21.327

Category > 3 23.710 8.381 11.859 47.404

Category > 4 30.956 15.554 11.562 82.880

VAXAUT

Category > 1 82.929 39.192 32.842 209.404

Category > 2 60.637 23.748 28.142 130.650

Category > 3 31.492 5.794 21.958 45.164

Category > 4 22.858 5.369 14.425 36.223

PRNTREFU

Category > 1 38.802 10.321 23.037 65.353

Category > 2 14.713 2.656 10.328 20.959

Category > 3 18.353 4.091 11.857 28.407

Category > 4 12.682 3.257 7.666 20.980

Latent Class 3 Compared to Latent Class 5

PROTCHLD

Category > 1 1.543 0.748 0.597 3.989

Category > 2 11.866 3.237 6.951 20.253

Category > 3 112.556 24.861 73.005 173.534

Category > 4 839.744 333.524 385.537 1829.061

DRRECVAX

Category > 1 4.906 1.928 2.271 10.600

Category > 2 30.554 6.786 19.770 47.221

Category > 3 46.552 7.537 33.894 63.938

Category > 4 89.863 23.218 54.157 149.110

NEWVAXSF

Category > 1 6.091 2.074 3.125 11.873

Category > 2 19.964 3.811 13.733 29.022

Category > 3 33.446 4.065 26.357 42.442

Category > 4 11.956 1.614 9.176 15.579

VAXACHV

Category > 1 1.784 0.969 0.615 5.175

Category > 2 23.662 6.746 13.532 41.375

Category > 3 150.632 33.454 97.470 232.789

Category > 4 432.360 146.610 222.436 840.401

CNCSDEFF

Category > 1 36.403 9.129 22.267 59.512

Category > 2 23.260 3.137 17.856 30.299

Category > 3 115.730 33.680 65.422 204.725

Category > 4 205.767 136.375 56.133 754.277

VAXAUT

Category > 1 36.621 19.304 13.032 102.906

Category > 2 80.501 36.175 33.365 194.228

Category > 3 78.649 16.650 51.938 119.097

Category > 4 105.301 30.014 60.230 184.099

PRNTREFU

Category > 1 30.377 6.926 19.430 47.492

Category > 2 24.637 3.496 18.656 32.536

Category > 3 79.544 16.234 53.319 118.668

Category > 4 99.596 38.476 46.709 212.364

Latent Class 4 Compared to Latent Class 5

PROTCHLD

Category > 1 2.884 2.988 0.379 21.966

Category > 2 11.984 6.404 4.205 34.156

Category > 3 34.766 11.551 18.127 66.675

Category > 4 229.933 88.771 107.887 490.040

DRRECVAX

Category > 1 0.936 0.466 0.353 2.485

Category > 2 6.687 2.023 3.696 12.100

Category > 3 13.293 2.736 8.880 19.899

Category > 4 55.150 14.776 32.620 93.241

NEWVAXSF

Category > 1 3.110 1.569 1.157 8.362

Category > 2 8.258 2.519 4.542 15.016

Category > 3 12.106 2.251 8.408 17.431

Category > 4 16.005 3.217 10.793 23.732

VAXACHV

Category > 1 6.602 8.756 0.491 88.844

Category > 2 52.553 79.901 2.669 1034.593

Category > 3 34.256 10.888 18.373 63.869

Category > 4 212.162 74.026 107.069 420.406

CNCSDEFF

Category > 1 0.852 0.262 0.467 1.556

Category > 2 1.688 0.469 0.979 2.909

Category > 3 4.881 2.388 1.871 12.733

Category > 4 6.647 5.554 1.292 34.189

VAXAUT

Category > 1 0.442 0.146 0.231 0.846

Category > 2 1.328 0.305 0.847 2.082

Category > 3 2.497 0.685 1.459 4.277

Category > 4 4.607 1.653 2.280 9.307

PRNTREFU

Category > 1 0.783 0.236 0.434 1.413

Category > 2 1.675 0.402 1.046 2.681

Category > 3 4.334 1.412 2.289 8.206

Category > 4 7.853 3.768 3.066 20.114

LOGISTIC REGRESSION ODDS RATIO RESULTS

95% C.I.

Estimate S.E. Lower 2.5% Upper 2.5%

Categorical Latent Variables

C#1 ON

CANADA 1.586 0.168 1.289 1.951

UK 2.543 0.285 2.042 3.168

C#2 ON

CANADA 1.417 0.217 1.050 1.913

UK 1.010 0.180 0.712 1.433

C#3 ON

CANADA 1.776 0.161 1.487 2.122

UK 2.916 0.297 2.388 3.560

C#4 ON

CANADA 1.432 0.212 1.071 1.914

UK 2.581 0.492 1.777 3.749

ALTERNATIVE PARAMETERIZATIONS FOR THE CATEGORICAL LATENT VARIABLE REGRESSION

Two-Tailed

Estimate S.E. Est./S.E. P-Value

Parameterization using Reference Class 1

C#2 ON

CANADA -0.112 0.134 -0.840 0.401

UK -0.924 0.160 -5.755 0.000

C#3 ON

CANADA 0.114 0.077 1.474 0.140

UK 0.137 0.082 1.667 0.096

C#4 ON

CANADA -0.102 0.142 -0.719 0.472

UK 0.015 0.191 0.078 0.938

C#5 ON

CANADA -0.461 0.106 -4.360 0.000

UK -0.933 0.112 -8.331 0.000

Intercepts

C#2 -1.314 0.161 -8.181 0.000

C#3 0.249 0.065 3.818 0.000

C#4 -0.943 0.140 -6.737 0.000

C#5 -0.114 0.096 -1.189 0.235

Parameterization using Reference Class 2

C#1 ON

CANADA 0.112 0.134 0.840 0.401

UK 0.924 0.160 5.755 0.000

C#3 ON

CANADA 0.226 0.129 1.744 0.081

UK 1.060 0.153 6.913 0.000

C#4 ON

CANADA 0.010 0.175 0.060 0.953

UK 0.938 0.225 4.177 0.000

C#5 ON

CANADA -0.349 0.153 -2.278 0.023

UK -0.010 0.178 -0.055 0.956

Intercepts

C#1 1.314 0.161 8.181 0.000

C#3 1.563 0.146 10.727 0.000

C#4 0.371 0.172 2.159 0.031

C#5 1.200 0.152 7.901 0.000

Parameterization using Reference Class 3

C#1 ON

CANADA -0.114 0.077 -1.474 0.140

UK -0.137 0.082 -1.667 0.096

C#2 ON

CANADA -0.226 0.129 -1.744 0.081

UK -1.060 0.153 -6.913 0.000

C#4 ON

CANADA -0.215 0.137 -1.572 0.116

UK -0.122 0.192 -0.633 0.526

C#5 ON

CANADA -0.575 0.091 -6.325 0.000

UK -1.070 0.102 -10.505 0.000

Intercepts

C#1 -0.249 0.065 -3.818 0.000

C#2 -1.563 0.146 -10.727 0.000

C#4 -1.192 0.135 -8.842 0.000

C#5 -0.363 0.079 -4.578 0.000

Parameterization using Reference Class 4

C#1 ON

CANADA 0.102 0.142 0.719 0.472

UK -0.015 0.191 -0.078 0.938

C#2 ON

CANADA -0.010 0.175 -0.060 0.953

UK -0.938 0.225 -4.177 0.000

C#3 ON

CANADA 0.215 0.137 1.572 0.116

UK 0.122 0.192 0.633 0.526

C#5 ON

CANADA -0.359 0.148 -2.425 0.015

UK -0.948 0.190 -4.978 0.000

Intercepts

C#1 0.943 0.140 6.737 0.000

C#2 -0.371 0.172 -2.159 0.031

C#3 1.192 0.135 8.842 0.000

C#5 0.829 0.126 6.569 0.000

ODDS RATIO FOR THE ALTERNATIVE PARAMETERIZATIONS FOR THE CATEGORICAL LATENT VARIABLE REGRESSION

95% C.I.

Estimate S.E. Lower 2.5% Upper 2.5%

Parameterization using Reference Class 1

C#2 ON

CANADA 0.894 0.120 0.688 1.162

UK 0.397 0.064 0.290 0.544

C#3 ON

CANADA 1.120 0.086 0.963 1.303

UK 1.147 0.094 0.976 1.347

C#4 ON

CANADA 0.903 0.128 0.684 1.192

UK 1.015 0.194 0.697 1.477

C#5 ON

CANADA 0.631 0.067 0.513 0.776

UK 0.393 0.044 0.316 0.490

Parameterization using Reference Class 2

C#1 ON

CANADA 1.119 0.150 0.861 1.454

UK 2.518 0.404 1.839 3.449

C#3 ON

CANADA 1.253 0.162 0.972 1.615

UK 2.887 0.443 2.138 3.900

C#4 ON

CANADA 1.010 0.176 0.718 1.423

UK 2.556 0.574 1.646 3.970

C#5 ON

CANADA 0.706 0.108 0.523 0.952

UK 0.990 0.177 0.698 1.405

Parameterization using Reference Class 3

C#1 ON

CANADA 0.893 0.069 0.768 1.038

UK 0.872 0.072 0.743 1.024

C#2 ON

CANADA 0.798 0.103 0.619 1.028

UK 0.346 0.053 0.256 0.468

C#4 ON

CANADA 0.806 0.111 0.616 1.055

UK 0.885 0.170 0.607 1.291

C#5 ON

CANADA 0.563 0.051 0.471 0.673

UK 0.343 0.035 0.281 0.419

Parameterization using Reference Class 4

C#1 ON

CANADA 1.107 0.157 0.839 1.462

UK 0.985 0.189 0.677 1.434

C#2 ON

CANADA 0.990 0.173 0.703 1.393

UK 0.391 0.088 0.252 0.608

C#3 ON

CANADA 1.240 0.170 0.948 1.623

UK 1.130 0.217 0.775 1.647

C#5 ON

CANADA 0.698 0.103 0.522 0.933

UK 0.387 0.074 0.267 0.563

BRANT WALD TEST FOR PROPORTIONAL ODDS

Degrees of

Chi-Square Freedom P-Value

PROTCHLD

Overall test 7.137 6 0.308

CANADA 1.713 3 0.634

UK 4.541 3 0.209

DRRECVAX

Overall test 3.624 6 0.727

CANADA 2.396 3 0.494

UK 1.261 3 0.738

NEWVAXSF

Overall test 4.338 6 0.631

CANADA 1.268 3 0.737

UK 2.365 3 0.500

VAXACHV

Overall test 3.146 6 0.790

CANADA 0.151 3 0.985

UK 2.236 3 0.525

CNCSDEFF

Overall test 3.000 6 0.809

CANADA 1.275 3 0.735

UK 1.806 3 0.614

VAXAUT

Overall test 13.750 6 0.033

CANADA 1.002 3 0.801

UK 13.202 3 0.004

PRNTREFU

Overall test 18.761 6 0.005

CANADA 0.549 3 0.908

UK 16.331 3 0.001

QUALITY OF NUMERICAL RESULTS

Condition Number for the Information Matrix 0.503E-04

(ratio of smallest to largest eigenvalue)

TECHNICAL 11 OUTPUT

Random Starts Specifications for the k-1 Class Analysis Model

Number of initial stage random starts 250

Number of final stage optimizations 250

VUONG-LO-MENDELL-RUBIN LIKELIHOOD RATIO TEST FOR 4 (H0) VERSUS 5 CLASSES

H0 Loglikelihood Value -105813.691

2 Times the Loglikelihood Difference 2384.508

Difference in the Number of Parameters 31

Mean 47122.294

Standard Deviation 46690.281

P-Value 0.8310

LO-MENDELL-RUBIN ADJUSTED LRT TEST

Value 2376.432

P-Value 0.8311

SAVEDATA INFORMATION

Save file

probability.csv

Order and format of variables

PROTCHLD F10.3

DRRECVAX F10.3

NEWVAXSF F10.3

VAXACHV F10.3

CNCSDEFF F10.3

VAXAUT F10.3

PRNTREFU F10.3

CANADA F10.3

UK F10.3

CPROB1 F10.3

CPROB2 F10.3

CPROB3 F10.3

CPROB4 F10.3

CPROB5 F10.3

C F10.3

WEIGHT F10.3

ID I6

Save file format

16F10.3 I6

Save file record length 10000

Save missing symbol *

DIAGRAM INFORMATION

Mplus diagrams are currently not available for Mixture analysis.

No diagram output was produced.

Beginning Time: 17:31:53

Ending Time: 17:41:17

Elapsed Time: 00:09:24

MUTHEN & MUTHEN

3463 Stoner Ave.

Los Angeles, CA 90066

Tel: (310) 391-9971

Fax: (310) 391-8971

Web: www.StatModel.com

Support: Support@StatModel.com

Copyright (c) 1998-2020 Muthen & Muthen

# United States

Mplus VERSION 8.5

MUTHEN & MUTHEN

06/15/2021 7:02 PM

INPUT INSTRUCTIONS

Title:

Stata2Mplus conversion for C:\Users\...\

List of variables converted shown below

RespondentID :

weight :

ProtChld : Getting vaccines is a good way to protect children from disease.

1: Strongly agree

2: Agree

3: Neither agree nor disagree

4: Disagree

5: Strongly disagree

DrRecVax : Generally, I do what my doctor recommends about vaccines.

1: Strongly agree

2: Agree

3: Neither agree nor disagree

4: Disagree

5: Strongly disagree

NewVaxSf : New vaccines are recommended only if they are safe.

1: Strongly agree

2: Agree

3: Neither agree nor disagree

4: Disagree

5: Strongly disagree

UnvxChld : Enough children are vaccinated that even unvaccinated children are safe from

1: Strongly agree

2: Agree

3: Neither agree nor disagree

4: Disagree

5: Strongly disagree

CncSdEff : I am concerned about serious side effects of vaccines.

1: Strongly agree

2: Agree

3: Neither agree nor disagree

4: Disagree

5: Strongly disagree

VaxAut : Some vaccines cause autism in healthy children.

1: Strongly agree

2: Agree

3: Neither agree nor disagree

4: Disagree

5: Strongly disagree

PrntRefu : Parents should have the right to refuse vaccines required for schools for any

1: Strongly agree

2: Agree

3: Neither agree nor disagree

4: Disagree

5: Strongly disagree

VaxAchv : Vaccinations are one of the most significant achievements in improving public

1: Strongly agree

2: Agree

3: Neither agree nor disagree

4: Disagree

5: Strongly disagree

group :

id :

Data:

File is C:\...\combinedformplus.dat ;

Variable:

Names are

RespondentID weight ProtChld DrRecVax NewVaxSf UnvxChld CncSdEff VaxAut

PrntRefu VaxAchv group id;

Missing are all (-9999) ;

Usevariables are ProtChld DrRecVax NewVaxSf VaxAchv

CncSdEff VaxAut PrntRefu ;

Categorical are ProtChld DrRecVax NewVaxSf VaxAchv

CncSdEff VaxAut PrntRefu;

Weight=weight;

Idvariable =id;

classes= c(5);

Useobservations are group==0;

Missing are all (-9999) ;

Analysis:

Stseed=1234;

Type = mixture ;

Starts= 250 250;

Model:

! %Overall%

! c on canada uk;

Output:

Tech11;

Savedata:

!File is probability.csv;

!Save is CPROB;

*** WARNING

Input line exceeded 90 characters. Some input may be truncated.

Stata2Mplus conversion for C:\Users\...\a

*** WARNING

Input line exceeded 90 characters. Some input may be truncated.

UnvxChld : Enough children are vaccinated that even unvaccinated children are safe from d

*** WARNING

Input line exceeded 90 characters. Some input may be truncated.

VaxAchv : Vaccinations are one of the most significant achievements in improving public h

*** WARNING

Data set contains cases with missing on all variables.

These cases were not included in the analysis.

Number of cases with missing on all variables: 4

4 WARNING(S) FOUND IN THE INPUT INSTRUCTIONS

Stata2Mplus conversion for C:\Users\...\

List of variables converted shown below

RespondentID :

weight :

ProtChld : Getting vaccines is a good way to protect children from disease.

1: Strongly agree

2: Agree

3: Neither agree nor disagree

4: Disagree

5: Strongly disagree

DrRecVax : Generally, I do what my doctor recommends about vaccines.

1: Strongly agree

2: Agree

3: Neither agree nor disagree

4: Disagree

5: Strongly disagree

NewVaxSf : New vaccines are recommended only if they are safe.

1: Strongly agree

2: Agree

3: Neither agree nor disagree

4: Disagree

5: Strongly disagree

UnvxChld : Enough children are vaccinated that even unvaccinated children are safe from

1: Strongly agree

2: Agree

3: Neither agree nor disagree

4: Disagree

5: Strongly disagree

CncSdEff : I am concerned about serious side effects of vaccines.

1: Strongly agree

2: Agree

3: Neither agree nor disagree

4: Disagree

5: Strongly disagree

VaxAut : Some vaccines cause autism in healthy children.

1: Strongly agree

2: Agree

3: Neither agree nor disagree

4: Disagree

5: Strongly disagree

PrntRefu : Parents should have the right to refuse vaccines required for schools for any

1: Strongly agree

2: Agree

3: Neither agree nor disagree

4: Disagree

5: Strongly disagree

VaxAchv : Vaccinations are one of the most significant achievements in improving public

1: Strongly agree

2: Agree

3: Neither agree nor disagree

4: Disagree

5: Strongly disagree

group :

id :

SUMMARY OF ANALYSIS

Number of groups 1

Number of observations 4612

Number of dependent variables 7

Number of independent variables 0

Number of continuous latent variables 0

Number of categorical latent variables 1

Observed dependent variables

Binary and ordered categorical (ordinal)

PROTCHLD DRRECVAX NEWVAXSF VAXACHV CNCSDEFF VAXAUT

PRNTREFU

Categorical latent variables

C

Variables with special functions

Weight variable WEIGHT

ID variable ID

Estimator MLR

Information matrix OBSERVED

Optimization Specifications for the Quasi-Newton Algorithm for

Continuous Outcomes

Maximum number of iterations 100

Convergence criterion 0.100D-05

Optimization Specifications for the EM Algorithm

Maximum number of iterations 500

Convergence criteria

Loglikelihood change 0.100D-06

Relative loglikelihood change 0.100D-06

Derivative 0.100D-05

Optimization Specifications for the M step of the EM Algorithm for

Categorical Latent variables

Number of M step iterations 1

M step convergence criterion 0.100D-05

Basis for M step termination ITERATION

Optimization Specifications for the M step of the EM Algorithm for

Censored, Binary or Ordered Categorical (Ordinal), Unordered

Categorical (Nominal) and Count Outcomes

Number of M step iterations 1

M step convergence criterion 0.100D-05

Basis for M step termination ITERATION

Maximum value for logit thresholds 15

Minimum value for logit thresholds -15

Minimum expected cell size for chi-square 0.100D-01

Maximum number of iterations for H1 2000

Convergence criterion for H1 0.100D-03

Optimization algorithm EMA

Random Starts Specifications

Number of initial stage random starts 250

Number of final stage optimizations 250

Number of initial stage iterations 10

Initial stage convergence criterion 0.100D+01

Random starts scale 0.500D+01

Random seed for generating random starts 1234

Link LOGIT

Input data file(s)

C:\...\

Input data format FREE

SUMMARY OF DATA

Number of missing data patterns 24

Number of y missing data patterns 0

Number of u missing data patterns 24

COVARIANCE COVERAGE OF DATA

Minimum covariance coverage value 0.100

PROPORTION OF DATA PRESENT FOR U

Covariance Coverage

PROTCHLD DRRECVAX NEWVAXSF VAXACHV CNCSDEFF

________ ________ ________ ________ ________

PROTCHLD 0.996

DRRECVAX 0.992 0.995

NEWVAXSF 0.993 0.992 0.996

VAXACHV 0.994 0.993 0.994 0.997

CNCSDEFF 0.993 0.992 0.992 0.994 0.996

VAXAUT 0.990 0.989 0.990 0.991 0.990

PRNTREFU 0.994 0.993 0.994 0.995 0.993

Covariance Coverage

VAXAUT PRNTREFU

________ ________

VAXAUT 0.993

PRNTREFU 0.991 0.997

UNIVARIATE PROPORTIONS AND COUNTS FOR CATEGORICAL VARIABLES

PROTCHLD

Category 1 0.039 178.046

Category 2 0.036 166.535

Category 3 0.142 652.957

Category 4 0.293 1347.889

Category 5 0.490 2251.319

DRRECVAX

Category 1 0.044 199.787

Category 2 0.072 332.068

Category 3 0.170 778.213

Category 4 0.367 1681.530

Category 5 0.348 1593.993

NEWVAXSF

Category 1 0.055 251.339

Category 2 0.082 378.090

Category 3 0.191 875.912

Category 4 0.345 1582.422

Category 5 0.328 1504.969

VAXACHV

Category 1 0.045 206.197

Category 2 0.051 234.051

Category 3 0.169 778.202

Category 4 0.298 1371.422

Category 5 0.436 2005.904

CNCSDEFF

Category 1 0.224 1030.203

Category 2 0.280 1287.261

Category 3 0.211 969.874

Category 4 0.175 803.301

Category 5 0.110 503.228

VAXAUT

Category 1 0.073 332.601

Category 2 0.106 483.982

Category 3 0.361 1652.866

Category 4 0.152 693.951

Category 5 0.308 1410.407

PRNTREFU

Category 1 0.185 848.446

Category 2 0.178 818.241

Category 3 0.170 781.164

Category 4 0.238 1094.144

Category 5 0.230 1056.096

RANDOM STARTS RESULTS RANKED FROM THE BEST TO THE WORST LOGLIKELIHOOD VALUES

Final stage loglikelihood values at local maxima, seeds, and initial stage start numbers:

-37066.632 54821 175

-37066.632 666361 161

-37066.632 248181 113

-37066.632 697240 164

-37066.632 622917 144

-37066.632 218792 16

-37066.632 450240 42

-37066.632 95220 111

-37066.632 147827 139

-37066.632 725324 125

-37066.632 963268 90

-37066.632 679380 92

-37066.632 757904 134

-37066.632 134946 209

-37066.632 699910 182

-37066.632 418410 101

-37066.632 723320 84

-37066.632 285532 120

-37066.632 79474 115

-37066.632 528306 23

-37066.632 773064 244

-37066.632 98826 88

-37067.790 574339 67

-37067.790 921481 159

-37173.793 286672 75

-37175.431 224532 116

-37175.431 872812 49

-37175.431 545354 183

-37183.899 668446 119

-37183.899 819868 28

Unperturbed starting value run did not converge or was rejected in the third stage.

219 perturbed starting value run(s) did not converge or were rejected in the third stage.

THE BEST LOGLIKELIHOOD VALUE HAS BEEN REPLICATED. RERUN WITH AT LEAST TWICE THE

RANDOM STARTS TO CHECK THAT THE BEST LOGLIKELIHOOD IS STILL OBTAINED AND REPLICATED.

IN THE OPTIMIZATION, ONE OR MORE LOGIT THRESHOLDS APPROACHED EXTREME VALUES

OF -15.000 AND 15.000 AND WERE FIXED TO STABILIZE MODEL ESTIMATION. THESE

VALUES IMPLY PROBABILITIES OF 0 AND 1. IN THE MODEL RESULTS SECTION, THESE

PARAMETERS HAVE 0 STANDARD ERRORS AND 999 IN THE Z-SCORE AND P-VALUE COLUMNS.

THE MODEL ESTIMATION TERMINATED NORMALLY

MODEL FIT INFORMATION

Number of Free Parameters 144

Loglikelihood

H0 Value -37066.632

H0 Scaling Correction Factor 1.9507

for MLR

Information Criteria

Akaike (AIC) 74421.263

Bayesian (BIC) 75348.107

Sample-Size Adjusted BIC 74890.530

(n* = (n + 2) / 24)

Chi-Square Test of Model Fit for the Binary and Ordered Categorical

(Ordinal) Outcomes**

Pearson Chi-Square

Value 33482.062

Degrees of Freedom 77841

P-Value 1.0000

Likelihood Ratio Chi-Square

Value 23794.839

Degrees of Freedom 77841

P-Value 1.0000

** Of the 217540 cells in the latent class indicator table, 139

were deleted in the calculation of chi-square due to extreme values.

Chi-Square Test for MCAR under the Unrestricted Latent Class Indicator Model

Pearson Chi-Square

Value 31470.483

Degrees of Freedom 139392

P-Value 1.0000

Likelihood Ratio Chi-Square

Value 30687.502

Degrees of Freedom 139392

P-Value 1.0000

FINAL CLASS COUNTS AND PROPORTIONS FOR THE LATENT CLASSES

BASED ON THE ESTIMATED MODEL

Latent

Classes

1 470.75449 0.10207

2 1293.17186 0.28039

3 1614.18920 0.35000

4 256.97440 0.05572

5 976.91005 0.21182

FINAL CLASS COUNTS AND PROPORTIONS FOR THE LATENT CLASSES

BASED ON ESTIMATED POSTERIOR PROBABILITIES

Latent

Classes

1 470.75449 0.10207

2 1293.17186 0.28039

3 1614.18920 0.35000

4 256.97440 0.05572

5 976.91005 0.21182

FINAL CLASS COUNTS AND PROPORTIONS FOR THE LATENT CLASSES

BASED ON THEIR MOST LIKELY LATENT CLASS MEMBERSHIP

Class Counts and Proportions

Latent

Classes

1 456 0.09886

2 1287 0.27899

3 1633 0.35418

4 251 0.05435

5 985 0.21362

CLASSIFICATION QUALITY

Entropy 0.859

Average Latent Class Probabilities for Most Likely Latent Class Membership (Row)

by Latent Class (Column)

1 2 3 4 5

1 0.828 0.069 0.075 0.005 0.023

2 0.026 0.893 0.032 0.000 0.049

3 0.028 0.030 0.942 0.000 0.000

4 0.001 0.000 0.002 0.954 0.043

5 0.014 0.065 0.000 0.015 0.906

Classification Probabilities for the Most Likely Latent Class Membership (Column)

by Latent Class (Row)

1 2 3 4 5

1 0.802 0.072 0.096 0.001 0.029

2 0.024 0.888 0.038 0.000 0.050

3 0.021 0.025 0.953 0.000 0.000

4 0.009 0.001 0.002 0.931 0.057

5 0.011 0.064 0.000 0.011 0.914

Logits for the Classification Probabilities for the Most Likely Latent Class Membership (Column)

by Latent Class (Row)

1 2 3 4 5

1 3.313 0.903 1.192 -3.794 0.000

2 -0.713 2.885 -0.276 -7.693 0.000

3 7.976 8.156 11.787 3.509 0.000

4 -1.875 -4.204 -3.281 2.784 0.000

5 -4.425 -2.652 -11.661 -4.415 0.000

MODEL RESULTS

Two-Tailed

Estimate S.E. Est./S.E. P-Value

Latent Class 1

Thresholds

PROTCHLD$1 -5.867 1.516 -3.870 0.000

PROTCHLD$2 -5.566 1.287 -4.324 0.000

PROTCHLD$3 -3.076 0.593 -5.188 0.000

PROTCHLD$4 -1.576 0.418 -3.773 0.000

DRRECVAX$1 -4.305 0.805 -5.350 0.000

DRRECVAX$2 -2.774 0.382 -7.253 0.000

DRRECVAX$3 -1.672 0.278 -6.008 0.000

DRRECVAX$4 -0.544 0.230 -2.360 0.018

NEWVAXSF$1 -3.368 0.580 -5.802 0.000

NEWVAXSF$2 -2.553 0.417 -6.119 0.000

NEWVAXSF$3 -1.250 0.239 -5.222 0.000

NEWVAXSF$4 -0.656 0.255 -2.578 0.010

VAXACHV$1 -15.000 0.000 999.000 999.000

VAXACHV$2 -4.925 1.161 -4.243 0.000

VAXACHV$3 -2.524 0.461 -5.470 0.000

VAXACHV$4 -1.200 0.400 -3.000 0.003

CNCSDEFF$1 0.326 0.336 0.969 0.332

CNCSDEFF$2 1.516 0.407 3.723 0.000

CNCSDEFF$3 2.736 0.490 5.578 0.000

CNCSDEFF$4 15.000 0.000 999.000 999.000

VAXAUT$1 -1.428 0.322 -4.436 0.000

VAXAUT$2 -0.796 0.286 -2.787 0.005

VAXAUT$3 1.532 0.367 4.177 0.000

VAXAUT$4 2.224 0.394 5.644 0.000

PRNTREFU$1 -0.151 0.311 -0.486 0.627

PRNTREFU$2 0.443 0.271 1.632 0.103

PRNTREFU$3 1.420 0.307 4.621 0.000

PRNTREFU$4 2.674 0.366 7.297 0.000

Latent Class 2

Thresholds

PROTCHLD$1 -8.245 3.891 -2.119 0.034

PROTCHLD$2 -4.674 0.614 -7.613 0.000

PROTCHLD$3 -2.916 0.283 -10.308 0.000

PROTCHLD$4 1.225 0.180 6.806 0.000

DRRECVAX$1 -6.246 1.042 -5.993 0.000

DRRECVAX$2 -3.338 0.280 -11.919 0.000

DRRECVAX$3 -1.548 0.142 -10.940 0.000

DRRECVAX$4 2.581 0.246 10.505 0.000

NEWVAXSF$1 -5.610 0.694 -8.080 0.000

NEWVAXSF$2 -2.439 0.162 -15.045 0.000

NEWVAXSF$3 -0.890 0.119 -7.458 0.000

NEWVAXSF$4 2.084 0.162 12.839 0.000

VAXACHV$1 -15.000 0.000 999.000 999.000

VAXACHV$2 -4.249 0.479 -8.861 0.000

VAXACHV$3 -2.031 0.213 -9.534 0.000

VAXACHV$4 1.797 0.199 9.028 0.000

CNCSDEFF$1 -2.270 0.206 -11.033 0.000

CNCSDEFF$2 0.125 0.111 1.125 0.261

CNCSDEFF$3 1.512 0.130 11.605 0.000

CNCSDEFF$4 4.314 0.431 10.002 0.000

VAXAUT$1 -5.097 0.782 -6.520 0.000

VAXAUT$2 -1.963 0.160 -12.303 0.000

VAXAUT$3 0.457 0.124 3.667 0.000

VAXAUT$4 1.944 0.148 13.118 0.000

PRNTREFU$1 -2.694 0.235 -11.443 0.000

PRNTREFU$2 -0.630 0.112 -5.645 0.000

PRNTREFU$3 0.275 0.123 2.242 0.025

PRNTREFU$4 2.755 0.218 12.622 0.000

Latent Class 3

Thresholds

PROTCHLD$1 -5.106 0.322 -15.862 0.000

PROTCHLD$2 -4.831 0.283 -17.049 0.000

PROTCHLD$3 -4.651 0.252 -18.458 0.000

PROTCHLD$4 -3.122 0.203 -15.359 0.000

DRRECVAX$1 -5.705 0.435 -13.108 0.000

DRRECVAX$2 -5.136 0.384 -13.369 0.000

DRRECVAX$3 -3.156 0.190 -16.583 0.000

DRRECVAX$4 -0.983 0.085 -11.586 0.000

NEWVAXSF$1 -4.924 0.371 -13.272 0.000

NEWVAXSF$2 -3.821 0.258 -14.784 0.000

NEWVAXSF$3 -2.417 0.138 -17.514 0.000

NEWVAXSF$4 -0.254 0.072 -3.516 0.000

VAXACHV$1 -5.041 0.411 -12.280 0.000

VAXACHV$2 -4.774 0.390 -12.247 0.000

VAXACHV$3 -4.057 0.262 -15.483 0.000

VAXACHV$4 -2.183 0.129 -16.891 0.000

CNCSDEFF$1 -3.636 0.287 -12.653 0.000

CNCSDEFF$2 -1.684 0.117 -14.372 0.000

CNCSDEFF$3 -0.492 0.088 -5.560 0.000

CNCSDEFF$4 0.927 0.087 10.660 0.000

VAXAUT$1 -7.028 1.166 -6.026 0.000

VAXAUT$2 -6.276 0.933 -6.725 0.000

VAXAUT$3 -2.195 0.183 -12.005 0.000

VAXAUT$4 -0.953 0.112 -8.542 0.000

PRNTREFU$1 -4.305 0.330 -13.030 0.000

PRNTREFU$2 -3.157 0.213 -14.813 0.000

PRNTREFU$3 -2.065 0.158 -13.083 0.000

PRNTREFU$4 -0.265 0.083 -3.202 0.001

Latent Class 4

Thresholds

PROTCHLD$1 0.522 0.517 1.008 0.313

PROTCHLD$2 1.386 0.399 3.474 0.001

PROTCHLD$3 2.600 0.718 3.620 0.000

PROTCHLD$4 3.131 0.565 5.538 0.000

DRRECVAX$1 0.441 0.374 1.179 0.239

DRRECVAX$2 1.282 0.282 4.541 0.000

DRRECVAX$3 2.190 0.381 5.744 0.000

DRRECVAX$4 2.947 0.506 5.827 0.000

NEWVAXSF$1 0.409 0.298 1.369 0.171

NEWVAXSF$2 0.804 0.239 3.365 0.001

NEWVAXSF$3 1.600 0.329 4.867 0.000

NEWVAXSF$4 1.714 0.292 5.868 0.000

VAXACHV$1 0.893 0.588 1.518 0.129

VAXACHV$2 1.631 0.503 3.242 0.001

VAXACHV$3 2.544 0.645 3.948 0.000

VAXACHV$4 3.270 0.512 6.389 0.000

CNCSDEFF$1 1.131 0.311 3.639 0.000

CNCSDEFF$2 1.416 0.357 3.968 0.000

CNCSDEFF$3 1.706 0.385 4.431 0.000

CNCSDEFF$4 2.010 0.429 4.686 0.000

VAXAUT$1 0.136 0.197 0.694 0.488

VAXAUT$2 0.562 0.236 2.378 0.017

VAXAUT$3 1.525 0.351 4.347 0.000

VAXAUT$4 1.632 0.392 4.165 0.000

PRNTREFU$1 1.471 0.270 5.450 0.000

PRNTREFU$2 1.724 0.328 5.256 0.000

PRNTREFU$3 1.879 0.362 5.185 0.000

PRNTREFU$4 2.009 0.401 5.008 0.000

Latent Class 5

Thresholds

PROTCHLD$1 -5.069 0.596 -8.511 0.000

PROTCHLD$2 -2.027 0.288 -7.031 0.000

PROTCHLD$3 0.730 0.184 3.965 0.000

PROTCHLD$4 4.024 0.655 6.142 0.000

DRRECVAX$1 -3.446 0.425 -8.112 0.000

DRRECVAX$2 -1.052 0.195 -5.385 0.000

DRRECVAX$3 1.039 0.176 5.915 0.000

DRRECVAX$4 3.637 0.486 7.482 0.000

NEWVAXSF$1 -2.619 0.286 -9.161 0.000

NEWVAXSF$2 -0.900 0.154 -5.837 0.000

NEWVAXSF$3 0.849 0.131 6.481 0.000

NEWVAXSF$4 2.074 0.196 10.606 0.000

VAXACHV$1 -4.252 0.459 -9.259 0.000

VAXACHV$2 -1.414 0.205 -6.905 0.000

VAXACHV$3 1.321 0.205 6.453 0.000

VAXACHV$4 4.967 0.715 6.950 0.000

CNCSDEFF$1 -0.351 0.183 -1.917 0.055

CNCSDEFF$2 1.470 0.168 8.745 0.000

CNCSDEFF$3 4.760 0.709 6.710 0.000

CNCSDEFF$4 15.000 0.000 999.000 999.000

VAXAUT$1 -2.195 0.278 -7.904 0.000

VAXAUT$2 -0.583 0.155 -3.768 0.000

VAXAUT$3 3.100 0.268 11.549 0.000

VAXAUT$4 4.536 0.417 10.889 0.000

PRNTREFU$1 -0.719 0.193 -3.733 0.000

PRNTREFU$2 0.690 0.149 4.623 0.000

PRNTREFU$3 3.114 0.257 12.112 0.000

PRNTREFU$4 4.763 0.591 8.060 0.000

Categorical Latent Variables

Means

C#1 -0.730 0.157 -4.648 0.000

C#2 0.280 0.102 2.760 0.006

C#3 0.502 0.093 5.424 0.000

C#4 -1.335 0.203 -6.587 0.000

RESULTS IN PROBABILITY SCALE

Latent Class 1

PROTCHLD

Category 1 0.003 0.004 0.661 0.508

Category 2 0.001 0.002 0.415 0.678

Category 3 0.040 0.025 1.637 0.102

Category 4 0.127 0.045 2.811 0.005

Category 5 0.829 0.059 13.970 0.000

DRRECVAX

Category 1 0.013 0.011 1.259 0.208

Category 2 0.045 0.019 2.352 0.019

Category 3 0.099 0.028 3.526 0.000

Category 4 0.209 0.046 4.586 0.000

Category 5 0.633 0.054 11.816 0.000

NEWVAXSF

Category 1 0.033 0.019 1.782 0.075

Category 2 0.039 0.022 1.775 0.076

Category 3 0.151 0.037 4.123 0.000

Category 4 0.119 0.037 3.226 0.001

Category 5 0.658 0.057 11.502 0.000

VAXACHV

Category 1 0.000 0.000 0.000 1.000

Category 2 0.007 0.008 0.868 0.385

Category 3 0.067 0.028 2.370 0.018

Category 4 0.157 0.058 2.724 0.006

Category 5 0.769 0.071 10.799 0.000

CNCSDEFF

Category 1 0.581 0.082 7.090 0.000

Category 2 0.239 0.043 5.595 0.000

Category 3 0.119 0.044 2.697 0.007

Category 4 0.061 0.028 2.171 0.030

Category 5 0.000 0.000 0.000 1.000

VAXAUT

Category 1 0.193 0.050 3.852 0.000

Category 2 0.117 0.028 4.253 0.000

Category 3 0.511 0.051 9.990 0.000

Category 4 0.080 0.036 2.251 0.024

Category 5 0.098 0.035 2.813 0.005

PRNTREFU

Category 1 0.462 0.077 5.985 0.000

Category 2 0.147 0.035 4.147 0.000

Category 3 0.196 0.037 5.375 0.000

Category 4 0.130 0.038 3.416 0.001

Category 5 0.064 0.022 2.917 0.004

Latent Class 2

PROTCHLD

Category 1 0.000 0.001 0.257 0.797

Category 2 0.009 0.005 1.674 0.094

Category 3 0.042 0.013 3.255 0.001

Category 4 0.722 0.030 23.821 0.000

Category 5 0.227 0.032 7.186 0.000

DRRECVAX

Category 1 0.002 0.002 0.961 0.336

Category 2 0.032 0.009 3.611 0.000

Category 3 0.141 0.018 8.032 0.000

Category 4 0.754 0.021 35.377 0.000

Category 5 0.070 0.016 4.378 0.000

NEWVAXSF

Category 1 0.004 0.003 1.445 0.148

Category 2 0.077 0.012 6.595 0.000

Category 3 0.211 0.021 9.995 0.000

Category 4 0.598 0.027 22.525 0.000

Category 5 0.111 0.016 6.928 0.000

VAXACHV

Category 1 0.000 0.000 0.000 1.000

Category 2 0.014 0.007 2.115 0.034

Category 3 0.102 0.021 4.781 0.000

Category 4 0.742 0.023 31.729 0.000

Category 5 0.142 0.024 5.855 0.000

CNCSDEFF

Category 1 0.094 0.017 5.361 0.000

Category 2 0.438 0.023 18.841 0.000

Category 3 0.288 0.020 14.360 0.000

Category 4 0.167 0.018 9.411 0.000

Category 5 0.013 0.006 2.350 0.019

VAXAUT

Category 1 0.006 0.005 1.287 0.198

Category 2 0.117 0.016 7.231 0.000

Category 3 0.489 0.025 19.261 0.000

Category 4 0.263 0.022 11.990 0.000

Category 5 0.125 0.016 7.713 0.000

PRNTREFU

Category 1 0.063 0.014 4.535 0.000

Category 2 0.284 0.021 13.550 0.000

Category 3 0.221 0.020 11.298 0.000

Category 4 0.372 0.025 14.746 0.000

Category 5 0.060 0.012 4.873 0.000

Latent Class 3

PROTCHLD

Category 1 0.006 0.002 3.125 0.002

Category 2 0.002 0.001 1.672 0.095

Category 3 0.002 0.001 1.958 0.050

Category 4 0.033 0.008 4.161 0.000

Category 5 0.958 0.008 116.549 0.000

DRRECVAX

Category 1 0.003 0.001 2.305 0.021

Category 2 0.003 0.002 1.470 0.142

Category 3 0.035 0.007 4.977 0.000

Category 4 0.231 0.015 15.240 0.000

Category 5 0.728 0.017 43.284 0.000

NEWVAXSF

Category 1 0.007 0.003 2.715 0.007

Category 2 0.014 0.005 2.999 0.003

Category 3 0.060 0.009 7.048 0.000

Category 4 0.355 0.017 21.017 0.000

Category 5 0.563 0.018 31.682 0.000

VAXACHV

Category 1 0.006 0.003 2.452 0.014

Category 2 0.002 0.002 1.002 0.316

Category 3 0.009 0.003 2.918 0.004

Category 4 0.084 0.011 7.815 0.000

Category 5 0.899 0.012 76.393 0.000

CNCSDEFF

Category 1 0.026 0.007 3.572 0.000

Category 2 0.131 0.013 10.014 0.000

Category 3 0.223 0.015 14.948 0.000

Category 4 0.337 0.015 22.485 0.000

Category 5 0.284 0.018 16.050 0.000

VAXAUT

Category 1 0.001 0.001 0.858 0.391

Category 2 0.001 0.001 0.790 0.429

Category 3 0.098 0.016 6.209 0.000

Category 4 0.178 0.014 12.767 0.000

Category 5 0.722 0.022 32.207 0.000

PRNTREFU

Category 1 0.013 0.004 3.067 0.002

Category 2 0.027 0.006 4.364 0.000

Category 3 0.072 0.011 6.450 0.000

Category 4 0.322 0.016 20.654 0.000

Category 5 0.566 0.020 27.846 0.000

Latent Class 4

PROTCHLD

Category 1 0.628 0.121 5.189 0.000

Category 2 0.172 0.073 2.362 0.018

Category 3 0.131 0.035 3.704 0.000

Category 4 0.027 0.041 0.670 0.503

Category 5 0.042 0.023 1.846 0.065

DRRECVAX

Category 1 0.608 0.089 6.831 0.000

Category 2 0.174 0.066 2.633 0.008

Category 3 0.117 0.035 3.369 0.001

Category 4 0.051 0.025 2.006 0.045

Category 5 0.050 0.024 2.081 0.037

NEWVAXSF

Category 1 0.601 0.072 8.395 0.000

Category 2 0.090 0.046 1.955 0.051

Category 3 0.141 0.034 4.096 0.000

Category 4 0.015 0.021 0.732 0.464

Category 5 0.153 0.038 4.041 0.000

VAXACHV

Category 1 0.710 0.121 5.851 0.000

Category 2 0.127 0.064 1.982 0.047

Category 3 0.091 0.038 2.388 0.017

Category 4 0.036 0.043 0.838 0.402

Category 5 0.037 0.018 2.028 0.043

CNCSDEFF

Category 1 0.756 0.057 13.189 0.000

Category 2 0.049 0.023 2.104 0.035

Category 3 0.042 0.021 1.942 0.052

Category 4 0.036 0.016 2.267 0.023

Category 5 0.118 0.045 2.644 0.008

VAXAUT

Category 1 0.534 0.049 10.921 0.000

Category 2 0.103 0.041 2.477 0.013

Category 3 0.184 0.040 4.637 0.000

Category 4 0.015 0.009 1.672 0.094

Category 5 0.164 0.054 3.050 0.002

PRNTREFU

Category 1 0.813 0.041 19.838 0.000

Category 2 0.035 0.022 1.597 0.110

Category 3 0.019 0.009 2.082 0.037

Category 4 0.014 0.006 2.544 0.011

Category 5 0.118 0.042 2.828 0.005

Latent Class 5

PROTCHLD

Category 1 0.006 0.004 1.690 0.091

Category 2 0.110 0.028 3.953 0.000

Category 3 0.558 0.033 16.720 0.000

Category 4 0.308 0.040 7.613 0.000

Category 5 0.018 0.011 1.554 0.120

DRRECVAX

Category 1 0.031 0.013 2.429 0.015

Category 2 0.228 0.030 7.491 0.000

Category 3 0.480 0.030 16.102 0.000

Category 4 0.236 0.032 7.329 0.000

Category 5 0.026 0.012 2.111 0.035

NEWVAXSF

Category 1 0.068 0.018 3.753 0.000

Category 2 0.221 0.023 9.650 0.000

Category 3 0.411 0.032 12.843 0.000

Category 4 0.188 0.024 7.862 0.000

Category 5 0.112 0.019 5.758 0.000

VAXACHV

Category 1 0.014 0.006 2.208 0.027

Category 2 0.182 0.029 6.193 0.000

Category 3 0.594 0.032 18.287 0.000

Category 4 0.204 0.033 6.209 0.000

Category 5 0.007 0.005 1.409 0.159

CNCSDEFF

Category 1 0.413 0.044 9.312 0.000

Category 2 0.400 0.034 11.728 0.000

Category 3 0.178 0.025 7.203 0.000

Category 4 0.008 0.006 1.422 0.155

Category 5 0.000 0.000 0.000 1.000

VAXAUT

Category 1 0.100 0.025 4.003 0.000

Category 2 0.258 0.023 11.310 0.000

Category 3 0.599 0.035 17.346 0.000

Category 4 0.032 0.010 3.204 0.001

Category 5 0.011 0.004 2.427 0.015

PRNTREFU

Category 1 0.328 0.042 7.727 0.000

Category 2 0.338 0.028 11.938 0.000

Category 3 0.291 0.032 9.165 0.000

Category 4 0.034 0.010 3.535 0.000

Category 5 0.008 0.005 1.707 0.088

LATENT CLASS ODDS RATIO RESULTS

95% C.I.

Estimate S.E. Lower 2.5% Upper 2.5%

Latent Class 1 Compared to Latent Class 2

PROTCHLD

Category > 1 0.093 0.400 0.000 434.961

Category > 2 2.439 3.522 0.144 41.340

Category > 3 1.173 0.801 0.308 4.473

Category > 4 16.468 7.224 6.970 38.909

DRRECVAX

Category > 1 0.144 0.209 0.008 2.479

Category > 2 0.569 0.289 0.210 1.540

Category > 3 1.132 0.367 0.599 2.138

Category > 4 22.759 6.808 12.663 40.906

NEWVAXSF

Category > 1 0.106 0.109 0.014 0.791

Category > 2 1.121 0.537 0.438 2.867

Category > 3 1.433 0.402 0.827 2.484

Category > 4 15.489 4.082 9.240 25.964

VAXACHV

Category > 1 1.000 0.000 1.000 1.000

Category > 2 1.967 2.581 0.150 25.734

Category > 3 1.637 0.866 0.581 4.618

Category > 4 20.039 8.579 8.659 46.374

CNCSDEFF

Category > 1 0.075 0.028 0.036 0.156

Category > 2 0.249 0.104 0.109 0.565

Category > 3 0.294 0.149 0.109 0.793

Category > 4 0.000 0.000 0.000 0.000

VAXAUT

Category > 1 0.026 0.022 0.005 0.133

Category > 2 0.311 0.103 0.162 0.597

Category > 3 0.341 0.133 0.159 0.732

Category > 4 0.756 0.325 0.325 1.756

PRNTREFU

Category > 1 0.079 0.030 0.037 0.166

Category > 2 0.342 0.100 0.193 0.606

Category > 3 0.318 0.103 0.169 0.601

Category > 4 1.084 0.462 0.470 2.500

Latent Class 1 Compared to Latent Class 3

PROTCHLD

Category > 1 2.142 3.331 0.102 45.145

Category > 2 2.086 2.797 0.151 28.871

Category > 3 0.207 0.134 0.058 0.736

Category > 4 0.213 0.098 0.086 0.526

DRRECVAX

Category > 1 0.247 0.227 0.041 1.502

Category > 2 0.094 0.054 0.030 0.292

Category > 3 0.227 0.078 0.116 0.445

Category > 4 0.645 0.158 0.398 1.043

NEWVAXSF

Category > 1 0.211 0.151 0.052 0.861

Category > 2 0.281 0.149 0.099 0.797

Category > 3 0.311 0.090 0.176 0.550

Category > 4 1.495 0.395 0.891 2.508

VAXACHV

Category > 1 21136.676 0.000 21136.678 21136.678

Category > 2 1.163 1.434 0.104 13.034

Category > 3 0.216 0.118 0.074 0.633

Category > 4 0.374 0.157 0.164 0.853

CNCSDEFF

Category > 1 0.019 0.007 0.009 0.040

Category > 2 0.041 0.016 0.019 0.086

Category > 3 0.040 0.019 0.016 0.100

Category > 4 0.000 0.000 0.000 0.000

VAXAUT

Category > 1 0.004 0.004 0.000 0.036

Category > 2 0.004 0.004 0.001 0.025

Category > 3 0.024 0.008 0.012 0.047

Category > 4 0.042 0.016 0.019 0.090

PRNTREFU

Category > 1 0.016 0.006 0.008 0.033

Category > 2 0.027 0.007 0.016 0.046

Category > 3 0.031 0.009 0.018 0.053

Category > 4 0.053 0.019 0.026 0.107

Latent Class 1 Compared to Latent Class 4

PROTCHLD

Category > 1 595.247 920.909 28.693 12348.603

Category > 2 1045.148 1378.837 78.739 13872.905

Category > 3 291.633 267.220 48.404 1757.101

Category > 4 110.764 77.491 28.111 436.432

DRRECVAX

Category > 1 115.125 101.109 20.587 643.804

Category > 2 57.759 26.909 23.177 143.943

Category > 3 47.585 21.902 19.306 117.289

Category > 4 32.798 17.775 11.338 94.880

NEWVAXSF

Category > 1 43.648 26.602 13.219 144.126

Category > 2 28.681 13.247 11.600 70.917

Category > 3 17.273 6.810 7.975 37.408

Category > 4 10.697 4.112 5.036 22.723

VAXACHV

Category > 1 ********* 0.000 ********* *********

Category > 2 703.752 896.692 57.921 8550.771

Category > 3 158.922 125.856 33.657 750.397

Category > 4 87.334 56.212 24.735 308.365

CNCSDEFF

Category > 1 2.236 1.062 0.882 5.670

Category > 2 0.905 0.507 0.302 2.712

Category > 3 0.357 0.227 0.103 1.240

Category > 4 0.000 0.000 0.000 0.000

VAXAUT

Category > 1 4.778 1.814 2.270 10.055

Category > 2 3.886 1.477 1.845 8.187

Category > 3 0.993 0.506 0.366 2.695

Category > 4 0.553 0.307 0.187 1.640

PRNTREFU

Category > 1 5.065 2.123 2.228 11.518

Category > 2 3.601 1.580 1.523 8.512

Category > 3 1.582 0.770 0.610 4.105

Category > 4 0.514 0.284 0.174 1.519

Latent Class 1 Compared to Latent Class 5

PROTCHLD

Category > 1 2.223 3.669 0.087 56.493

Category > 2 34.440 44.808 2.689 441.110

Category > 3 44.953 27.220 13.719 147.295

Category > 4 270.488 180.945 72.899 1003.639

DRRECVAX

Category > 1 2.362 2.161 0.393 14.187

Category > 2 5.593 2.407 2.406 13.001

Category > 3 15.049 4.880 7.970 28.416

Category > 4 65.444 32.867 24.456 175.130

NEWVAXSF

Category > 1 2.114 1.304 0.631 7.082

Category > 2 5.219 2.289 2.209 12.329

Category > 3 8.152 2.220 4.781 13.902

Category > 4 15.331 4.827 8.271 28.417

VAXACHV

Category > 1 46517.832 0.000 46517.828 46517.828

Category > 2 33.483 39.731 3.272 342.680

Category > 3 46.777 22.579 18.161 120.479

Category > 4 476.676 338.808 118.359 1919.746

CNCSDEFF

Category > 1 0.508 0.202 0.233 1.106

Category > 2 0.955 0.427 0.397 2.295

Category > 3 7.572 6.568 1.383 41.452

Category > 4 1.000 0.000 1.000 1.000

VAXAUT

Category > 1 0.464 0.197 0.202 1.068

Category > 2 1.238 0.403 0.654 2.341

Category > 3 4.801 2.205 1.951 11.811

Category > 4 10.091 5.855 3.236 31.467

PRNTREFU

Category > 1 0.567 0.211 0.273 1.177

Category > 2 1.281 0.394 0.702 2.339

Category > 3 5.442 2.142 2.516 11.773

Category > 4 8.070 5.834 1.956 33.287

Latent Class 2 Compared to Latent Class 3

PROTCHLD

Category > 1 23.084 91.895 0.009 56472.559

Category > 2 0.855 0.604 0.214 3.414

Category > 3 0.177 0.069 0.082 0.378

Category > 4 0.013 0.003 0.008 0.020

DRRECVAX

Category > 1 1.718 1.947 0.186 15.840

Category > 2 0.166 0.078 0.066 0.416

Category > 3 0.200 0.047 0.126 0.318

Category > 4 0.028 0.007 0.018 0.045

NEWVAXSF

Category > 1 1.986 1.604 0.408 9.670

Category > 2 0.251 0.075 0.139 0.452

Category > 3 0.217 0.038 0.154 0.306

Category > 4 0.097 0.017 0.069 0.136

VAXACHV

Category > 1 21136.676 0.000 21136.678 21136.678

Category > 2 0.591 0.397 0.158 2.207

Category > 3 0.132 0.045 0.068 0.256

Category > 4 0.019 0.004 0.013 0.028

CNCSDEFF

Category > 1 0.255 0.090 0.128 0.510

Category > 2 0.164 0.025 0.121 0.222

Category > 3 0.135 0.020 0.101 0.179

Category > 4 0.034 0.015 0.014 0.079

VAXAUT

Category > 1 0.145 0.210 0.008 2.479

Category > 2 0.013 0.013 0.002 0.085

Category > 3 0.071 0.014 0.047 0.105

Category > 4 0.055 0.009 0.040 0.076

PRNTREFU

Category > 1 0.200 0.082 0.089 0.447

Category > 2 0.080 0.018 0.051 0.126

Category > 3 0.096 0.018 0.067 0.139

Category > 4 0.049 0.011 0.032 0.075

Latent Class 2 Compared to Latent Class 4

PROTCHLD

Category > 1 6416.140 25689.314 2.507 *********

Category > 2 428.483 321.488 98.462 1864.647

Category > 3 248.579 178.912 60.647 1018.868

Category > 4 6.726 3.979 2.110 21.442

DRRECVAX

Category > 1 801.404 890.712 90.735 7078.279

Category > 2 101.505 39.864 47.010 219.173

Category > 3 42.037 16.770 19.233 91.879

Category > 4 1.441 0.814 0.476 4.359

NEWVAXSF

Category > 1 411.030 318.078 90.189 1873.236

Category > 2 25.587 7.271 14.660 44.659

Category > 3 12.051 4.000 6.288 23.097

Category > 4 0.691 0.227 0.362 1.317

VAXACHV

Category > 1 ********* 0.000 ********* *********

Category > 2 357.710 254.264 88.813 1440.741

Category > 3 97.057 60.239 28.755 327.598

Category > 4 4.358 2.449 1.448 13.113

CNCSDEFF

Category > 1 30.006 11.215 14.423 62.424

Category > 2 3.637 1.404 1.706 7.753

Category > 3 1.214 0.520 0.524 2.810

Category > 4 0.100 0.063 0.029 0.342

VAXAUT

Category > 1 187.328 150.366 38.845 903.374

Category > 2 12.487 3.614 7.081 22.020

Category > 3 2.910 1.148 1.343 6.304

Category > 4 0.732 0.323 0.308 1.737

PRNTREFU

Category > 1 64.429 22.942 32.061 129.474

Category > 2 10.527 3.715 5.272 21.023

Category > 3 4.971 1.997 2.261 10.926

Category > 4 0.474 0.227 0.185 1.211

Latent Class 2 Compared to Latent Class 5

PROTCHLD

Category > 1 23.957 97.818 0.008 71622.180

Category > 2 14.119 10.048 3.500 56.958

Category > 3 38.317 10.936 21.900 67.039

Category > 4 16.425 11.406 4.211 64.061

DRRECVAX

Category > 1 16.443 19.036 1.700 159.007

Category > 2 9.829 3.269 5.122 18.862

Category > 3 13.294 2.441 9.276 19.052

Category > 4 2.875 1.560 0.993 8.325

NEWVAXSF

Category > 1 19.910 15.441 4.355 91.036

Category > 2 4.656 1.020 3.030 7.154

Category > 3 5.688 0.941 4.113 7.867

Category > 4 0.990 0.264 0.587 1.669

VAXACHV

Category > 1 46517.832 0.000 46517.828 46517.828

Category > 2 17.019 9.225 5.882 49.243

Category > 3 28.568 6.771 17.953 45.459

Category > 4 23.788 17.174 5.778 97.927

CNCSDEFF

Category > 1 6.819 1.809 4.054 11.470

Category > 2 3.839 0.760 2.604 5.659

Category > 3 25.748 18.462 6.316 104.977

Category > 4 43744.574 0.000 43744.582 43744.582

VAXAUT

Category > 1 18.208 15.228 3.534 93.796

Category > 2 3.976 0.862 2.600 6.082

Category > 3 14.066 3.924 8.142 24.303

Category > 4 13.347 5.889 5.621 31.694

PRNTREFU

Category > 1 7.211 2.149 4.020 12.933

Category > 2 3.745 0.682 2.621 5.351

Category > 3 17.101 4.597 10.098 28.962

Category > 4 7.444 4.912 2.042 27.131

Latent Class 3 Compared to Latent Class 4

PROTCHLD

Category > 1 277.941 169.467 84.130 918.244

Category > 2 501.004 242.581 193.950 1294.172

Category > 3 1408.377 1057.984 323.056 6139.883

Category > 4 519.640 311.573 160.442 1683.011

DRRECVAX

Category > 1 466.523 270.437 149.773 1453.153

Category > 2 612.953 289.529 242.859 1547.039

Category > 3 209.898 88.752 91.639 480.764

Category > 4 50.889 26.125 18.605 139.194

NEWVAXSF

Category > 1 207.012 100.499 79.938 536.091

Category > 2 101.925 35.682 51.320 202.429

Category > 3 55.539 19.537 27.872 110.671

Category > 4 7.155 2.148 3.972 12.888

VAXACHV

Category > 1 377.786 275.748 90.352 1579.623

Category > 2 605.076 383.173 174.890 2093.414

Category > 3 736.266 511.239 188.790 2871.387

Category > 4 233.360 124.418 82.071 663.530

CNCSDEFF

Category > 1 117.506 50.555 50.564 273.076

Category > 2 22.204 8.498 10.487 47.011

Category > 3 9.004 3.621 4.094 19.805

Category > 4 2.953 1.313 1.235 7.061

VAXAUT

Category > 1 1292.057 1529.061 127.035 13141.319

Category > 2 932.071 908.831 137.866 6301.475

Category > 3 41.269 16.824 18.561 91.757

Category > 4 13.270 5.557 5.840 30.153

PRNTREFU

Category > 1 322.703 138.554 139.100 748.648

Category > 2 131.756 52.912 59.970 289.473

Category > 3 51.600 20.834 23.387 113.849

Category > 4 9.713 4.040 4.299 21.948

Latent Class 3 Compared to Latent Class 5

PROTCHLD

Category > 1 1.038 0.690 0.282 3.820

Category > 2 16.509 6.540 7.594 35.888

Category > 3 217.091 67.196 118.349 398.215

Category > 4 1268.972 874.912 328.525 4901.583

DRRECVAX

Category > 1 9.572 5.886 2.868 31.948

Category > 2 59.357 25.212 25.817 136.468

Category > 3 66.380 16.379 40.927 107.664

Category > 4 101.542 49.764 38.857 265.348

NEWVAXSF

Category > 1 10.028 4.779 3.941 25.517

Category > 2 18.545 5.382 10.500 32.755

Category > 3 26.213 4.717 18.423 37.297

Category > 4 10.255 2.166 6.779 15.513

VAXACHV

Category > 1 2.201 1.373 0.648 7.475

Category > 2 28.788 12.434 12.347 67.124

Category > 3 216.711 70.528 114.512 410.118

Category > 4 1273.689 915.030 311.560 5206.976

CNCSDEFF

Category > 1 26.704 9.244 13.549 52.632

Category > 2 23.435 4.789 15.701 34.978

Category > 3 190.981 136.071 47.262 771.736

Category > 4 ********* 0.000 ********* *********

VAXAUT

Category > 1 125.583 150.626 11.967 1317.921

Category > 2 296.816 276.899 47.686 1847.502

Category > 3 199.492 63.415 106.989 371.974

Category > 4 241.944 102.732 105.264 556.097

PRNTREFU

Category > 1 36.115 14.016 16.878 77.277

Category > 2 46.874 11.910 28.488 77.127

Category > 3 177.518 50.918 101.177 311.459

Category > 4 152.543 90.853 47.469 490.197

Latent Class 4 Compared to Latent Class 5

PROTCHLD

Category > 1 0.004 0.002 0.001 0.011

Category > 2 0.033 0.011 0.017 0.065

Category > 3 0.154 0.107 0.040 0.600

Category > 4 2.442 2.186 0.422 14.117

DRRECVAX

Category > 1 0.021 0.007 0.010 0.042

Category > 2 0.097 0.028 0.055 0.172

Category > 3 0.316 0.129 0.142 0.703

Category > 4 1.995 1.373 0.518 7.688

NEWVAXSF

Category > 1 0.048 0.014 0.028 0.084

Category > 2 0.182 0.046 0.110 0.300

Category > 3 0.472 0.168 0.235 0.949

Category > 4 1.433 0.533 0.691 2.972

VAXACHV

Category > 1 0.006 0.003 0.002 0.016

Category > 2 0.048 0.020 0.021 0.109

Category > 3 0.294 0.195 0.080 1.082

Category > 4 5.458 4.890 0.943 31.601

CNCSDEFF

Category > 1 0.227 0.098 0.098 0.530

Category > 2 1.055 0.472 0.439 2.537

Category > 3 21.210 17.716 4.126 109.029

Category > 4 ********* 0.000 ********* *********

VAXAUT

Category > 1 0.097 0.033 0.050 0.189

Category > 2 0.318 0.104 0.168 0.602

Category > 3 4.834 2.251 1.941 12.040

Category > 4 18.233 10.613 5.826 57.059

PRNTREFU

Category > 1 0.112 0.042 0.054 0.232

Category > 2 0.356 0.144 0.161 0.788

Category > 3 3.440 1.598 1.384 8.551

Category > 4 15.705 10.596 4.185 58.929

QUALITY OF NUMERICAL RESULTS

Condition Number for the Information Matrix 0.152E-04

(ratio of smallest to largest eigenvalue)

TECHNICAL 11 OUTPUT

Random Starts Specifications for the k-1 Class Analysis Model

Number of initial stage random starts 250

Number of final stage optimizations 250

VUONG-LO-MENDELL-RUBIN LIKELIHOOD RATIO TEST FOR 4 (H0) VERSUS 5 CLASSES

H0 Loglikelihood Value -37581.778

2 Times the Loglikelihood Difference 1030.292

Difference in the Number of Parameters 29

Mean 137.330

Standard Deviation 627.170

P-Value 0.0773

LO-MENDELL-RUBIN ADJUSTED LRT TEST

Value 1026.098

P-Value 0.0782

DIAGRAM INFORMATION

Mplus diagrams are currently not available for Mixture analysis.

No diagram output was produced.

Beginning Time: 19:02:32

Ending Time: 19:04:28

Elapsed Time: 00:01:56

MUTHEN & MUTHEN

3463 Stoner Ave.

Los Angeles, CA 90066

Tel: (310) 391-9971

Fax: (310) 391-8971

Web: www.StatModel.com

Support: Support@StatModel.com

Copyright (c) 1998-2020 Muthen & Muthen

# United Kingdom

Mplus VERSION 8.5

MUTHEN & MUTHEN

06/15/2021 7:09 PM

INPUT INSTRUCTIONS

Title:

Stata2Mplus conversion for C:\Users\...\

List of variables converted shown below

RespondentID :

weight :

ProtChld : Getting vaccines is a good way to protect children from disease.

1: Strongly agree

2: Agree

3: Neither agree nor disagree

4: Disagree

5: Strongly disagree

DrRecVax : Generally, I do what my doctor recommends about vaccines.

1: Strongly agree

2: Agree

3: Neither agree nor disagree

4: Disagree

5: Strongly disagree

NewVaxSf : New vaccines are recommended only if they are safe.

1: Strongly agree

2: Agree

3: Neither agree nor disagree

4: Disagree

5: Strongly disagree

UnvxChld : Enough children are vaccinated that even unvaccinated children are safe from

1: Strongly agree

2: Agree

3: Neither agree nor disagree

4: Disagree

5: Strongly disagree

CncSdEff : I am concerned about serious side effects of vaccines.

1: Strongly agree

2: Agree

3: Neither agree nor disagree

4: Disagree

5: Strongly disagree

VaxAut : Some vaccines cause autism in healthy children.

1: Strongly agree

2: Agree

3: Neither agree nor disagree

4: Disagree

5: Strongly disagree

PrntRefu : Parents should have the right to refuse vaccines required for schools for any

1: Strongly agree

2: Agree

3: Neither agree nor disagree

4: Disagree

5: Strongly disagree

VaxAchv : Vaccinations are one of the most significant achievements in improving public

1: Strongly agree

2: Agree

3: Neither agree nor disagree

4: Disagree

5: Strongly disagree

group :

id :

Data:

File is C:\...\combinedformplus.dat ;

Variable:

Names are

RespondentID weight ProtChld DrRecVax NewVaxSf UnvxChld CncSdEff VaxAut

PrntRefu VaxAchv group id;

Missing are all (-9999) ;

Usevariables are ProtChld DrRecVax NewVaxSf VaxAchv

CncSdEff VaxAut PrntRefu ;

Categorical are ProtChld DrRecVax NewVaxSf VaxAchv

CncSdEff VaxAut PrntRefu;

Weight=weight;

Idvariable =id;

classes= c(5);

Useobservations are group==2;

Missing are all (-9999) ;

Analysis:

Stseed=1234;

Type = mixture ;

Starts= 250 250;

Model:

! %Overall%

! c on canada uk;

Output:

Tech11;

Savedata:

!File is probability.csv;

!Save is CPROB;

*** WARNING

Input line exceeded 90 characters. Some input may be truncated.

Stata2Mplus conversion for C:\Users\...\a

*** WARNING

Input line exceeded 90 characters. Some input may be truncated.

UnvxChld : Enough children are vaccinated that even unvaccinated children are safe from d

*** WARNING

Input line exceeded 90 characters. Some input may be truncated.

VaxAchv : Vaccinations are one of the most significant achievements in improving public h

*** WARNING

Data set contains cases with missing on all variables.

These cases were not included in the analysis.

Number of cases with missing on all variables: 2

4 WARNING(S) FOUND IN THE INPUT INSTRUCTIONS

Stata2Mplus conversion for C:\Users\...\

List of variables converted shown below

RespondentID :

weight :

ProtChld : Getting vaccines is a good way to protect children from disease.

1: Strongly agree

2: Agree

3: Neither agree nor disagree

4: Disagree

5: Strongly disagree

DrRecVax : Generally, I do what my doctor recommends about vaccines.

1: Strongly agree

2: Agree

3: Neither agree nor disagree

4: Disagree

5: Strongly disagree

NewVaxSf : New vaccines are recommended only if they are safe.

1: Strongly agree

2: Agree

3: Neither agree nor disagree

4: Disagree

5: Strongly disagree

UnvxChld : Enough children are vaccinated that even unvaccinated children are safe from

1: Strongly agree

2: Agree

3: Neither agree nor disagree

4: Disagree

5: Strongly disagree

CncSdEff : I am concerned about serious side effects of vaccines.

1: Strongly agree

2: Agree

3: Neither agree nor disagree

4: Disagree

5: Strongly disagree

VaxAut : Some vaccines cause autism in healthy children.

1: Strongly agree

2: Agree

3: Neither agree nor disagree

4: Disagree

5: Strongly disagree

PrntRefu : Parents should have the right to refuse vaccines required for schools for any

1: Strongly agree

2: Agree

3: Neither agree nor disagree

4: Disagree

5: Strongly disagree

VaxAchv : Vaccinations are one of the most significant achievements in improving public

1: Strongly agree

2: Agree

3: Neither agree nor disagree

4: Disagree

5: Strongly disagree

group :

id :

SUMMARY OF ANALYSIS

Number of groups 1

Number of observations 4550

Number of dependent variables 7

Number of independent variables 0

Number of continuous latent variables 0

Number of categorical latent variables 1

Observed dependent variables

Binary and ordered categorical (ordinal)

PROTCHLD DRRECVAX NEWVAXSF VAXACHV CNCSDEFF VAXAUT

PRNTREFU

Categorical latent variables

C

Variables with special functions

Weight variable WEIGHT

ID variable ID

Estimator MLR

Information matrix OBSERVED

Optimization Specifications for the Quasi-Newton Algorithm for

Continuous Outcomes

Maximum number of iterations 100

Convergence criterion 0.100D-05

Optimization Specifications for the EM Algorithm

Maximum number of iterations 500

Convergence criteria

Loglikelihood change 0.100D-06

Relative loglikelihood change 0.100D-06

Derivative 0.100D-05

Optimization Specifications for the M step of the EM Algorithm for

Categorical Latent variables

Number of M step iterations 1

M step convergence criterion 0.100D-05

Basis for M step termination ITERATION

Optimization Specifications for the M step of the EM Algorithm for

Censored, Binary or Ordered Categorical (Ordinal), Unordered

Categorical (Nominal) and Count Outcomes

Number of M step iterations 1

M step convergence criterion 0.100D-05

Basis for M step termination ITERATION

Maximum value for logit thresholds 15

Minimum value for logit thresholds -15

Minimum expected cell size for chi-square 0.100D-01

Maximum number of iterations for H1 2000

Convergence criterion for H1 0.100D-03

Optimization algorithm EMA

Random Starts Specifications

Number of initial stage random starts 250

Number of final stage optimizations 250

Number of initial stage iterations 10

Initial stage convergence criterion 0.100D+01

Random starts scale 0.500D+01

Random seed for generating random starts 1234

Link LOGIT

Input data file(s)

C:\...\

Input data format FREE

SUMMARY OF DATA

Number of missing data patterns 21

Number of y missing data patterns 0

Number of u missing data patterns 21

COVARIANCE COVERAGE OF DATA

Minimum covariance coverage value 0.100

PROPORTION OF DATA PRESENT FOR U

Covariance Coverage

PROTCHLD DRRECVAX NEWVAXSF VAXACHV CNCSDEFF

________ ________ ________ ________ ________

PROTCHLD 0.997

DRRECVAX 0.995 0.997

NEWVAXSF 0.994 0.994 0.996

VAXACHV 0.995 0.995 0.995 0.997

CNCSDEFF 0.994 0.994 0.993 0.995 0.997

VAXAUT 0.993 0.993 0.993 0.995 0.993

PRNTREFU 0.994 0.994 0.993 0.995 0.994

Covariance Coverage

VAXAUT PRNTREFU

________ ________

VAXAUT 0.996

PRNTREFU 0.994 0.997

UNIVARIATE PROPORTIONS AND COUNTS FOR CATEGORICAL VARIABLES

PROTCHLD

Category 1 0.022 100.964

Category 2 0.015 67.132

Category 3 0.077 350.618

Category 4 0.286 1293.727

Category 5 0.600 2718.108

DRRECVAX

Category 1 0.023 106.243

Category 2 0.048 217.245

Category 3 0.126 572.080

Category 4 0.351 1586.108

Category 5 0.451 2041.824

NEWVAXSF

Category 1 0.022 99.748

Category 2 0.035 159.815

Category 3 0.107 486.050

Category 4 0.334 1511.076

Category 5 0.501 2267.862

VAXACHV

Category 1 0.020 92.810

Category 2 0.022 98.596

Category 3 0.099 448.754

Category 4 0.284 1286.997

Category 5 0.575 2602.432

CNCSDEFF

Category 1 0.106 478.529

Category 2 0.195 883.823

Category 3 0.240 1087.107

Category 4 0.268 1215.609

Category 5 0.190 862.364

VAXAUT

Category 1 0.041 185.918

Category 2 0.070 314.871

Category 3 0.364 1649.390

Category 4 0.189 855.829

Category 5 0.336 1520.289

PRNTREFU

Category 1 0.119 536.597

Category 2 0.206 932.444

Category 3 0.206 930.682

Category 4 0.287 1299.359

Category 5 0.183 826.316

RANDOM STARTS RESULTS RANKED FROM THE BEST TO THE WORST LOGLIKELIHOOD VALUES

Final stage loglikelihood values at local maxima, seeds, and initial stage start numbers:

-34246.739 774560 2

-34246.739 872812 49

-34246.739 36496 173

-34246.739 98826 88

-34246.739 717812 190

-34246.739 134946 209

-34246.739 559172 204

-34246.739 389320 106

-34246.739 644025 162

-34246.739 286672 75

-34246.739 30702 168

-34246.739 45321 176

-34246.739 528306 23

-34246.739 107463 196

-34246.739 147827 139

-34246.739 908193 203

-34246.739 879884 37

-34246.739 199792 146

-34246.739 138304 65

-34246.739 385052 76

-34246.739 63044 105

-34246.739 418410 101

-34246.739 723320 84

-34246.739 679380 92

-34246.739 569230 44

-34246.739 410009 68

-34246.739 697240 164

-34246.739 107886 9

-34246.967 718545 218

-34246.967 581706 226

-34246.967 392026 110

-34254.863 923515 198

-34285.401 571880 41

-34285.401 326222 136

Unperturbed starting value run did not converge or was rejected in the third stage.

215 perturbed starting value run(s) did not converge or were rejected in the third stage.

THE BEST LOGLIKELIHOOD VALUE HAS BEEN REPLICATED. RERUN WITH AT LEAST TWICE THE

RANDOM STARTS TO CHECK THAT THE BEST LOGLIKELIHOOD IS STILL OBTAINED AND REPLICATED.

IN THE OPTIMIZATION, ONE OR MORE LOGIT THRESHOLDS APPROACHED EXTREME VALUES

OF -15.000 AND 15.000 AND WERE FIXED TO STABILIZE MODEL ESTIMATION. THESE

VALUES IMPLY PROBABILITIES OF 0 AND 1. IN THE MODEL RESULTS SECTION, THESE

PARAMETERS HAVE 0 STANDARD ERRORS AND 999 IN THE Z-SCORE AND P-VALUE COLUMNS.

THE MODEL ESTIMATION TERMINATED NORMALLY

MODEL FIT INFORMATION

Number of Free Parameters 144

Loglikelihood

H0 Value -34246.739

H0 Scaling Correction Factor 1.8192

for MLR

Information Criteria

Akaike (AIC) 68781.478

Bayesian (BIC) 69706.373

Sample-Size Adjusted BIC 69248.797

(n* = (n + 2) / 24)

Chi-Square Test of Model Fit for the Binary and Ordered Categorical

(Ordinal) Outcomes**

Pearson Chi-Square

Value 28453.546

Degrees of Freedom 77853

P-Value 1.0000

Likelihood Ratio Chi-Square

Value 19855.812

Degrees of Freedom 77853

P-Value 1.0000

** Of the 204645 cells in the latent class indicator table, 127

were deleted in the calculation of chi-square due to extreme values.

Chi-Square Test for MCAR under the Unrestricted Latent Class Indicator Model

Pearson Chi-Square

Value 24142.188

Degrees of Freedom 126500

P-Value 1.0000

Likelihood Ratio Chi-Square

Value 23418.241

Degrees of Freedom 126500

P-Value 1.0000

FINAL CLASS COUNTS AND PROPORTIONS FOR THE LATENT CLASSES

BASED ON THE ESTIMATED MODEL

Latent

Classes

1 1358.01444 0.29846

2 623.44038 0.13702

3 623.46540 0.13703

4 165.26164 0.03632

5 1779.81815 0.39117

FINAL CLASS COUNTS AND PROPORTIONS FOR THE LATENT CLASSES

BASED ON ESTIMATED POSTERIOR PROBABILITIES

Latent

Classes

1 1358.01444 0.29846

2 623.44038 0.13702

3 623.46540 0.13703

4 165.26164 0.03632

5 1779.81815 0.39117

FINAL CLASS COUNTS AND PROPORTIONS FOR THE LATENT CLASSES

BASED ON THEIR MOST LIKELY LATENT CLASS MEMBERSHIP

Class Counts and Proportions

Latent

Classes

1 1381 0.30349

2 603 0.13252

3 574 0.12612

4 154 0.03382

5 1838 0.40404

CLASSIFICATION QUALITY

Entropy 0.809

Average Latent Class Probabilities for Most Likely Latent Class Membership (Row)

by Latent Class (Column)

1 2 3 4 5

1 0.869 0.055 0.038 0.000 0.037

2 0.074 0.880 0.018 0.027 0.000

3 0.084 0.018 0.788 0.001 0.109

4 0.001 0.039 0.005 0.954 0.001

5 0.035 0.000 0.058 0.000 0.906

Classification Probabilities for the Most Likely Latent Class Membership (Column)

by Latent Class (Row)

1 2 3 4 5

1 0.884 0.033 0.036 0.000 0.048

2 0.122 0.851 0.017 0.010 0.000

3 0.084 0.017 0.725 0.001 0.172

4 0.004 0.100 0.005 0.888 0.004

5 0.029 0.000 0.035 0.000 0.936

Logits for the Classification Probabilities for the Most Likely Latent Class Membership (Column)

by Latent Class (Row)

1 2 3 4 5

1 2.921 -0.367 -0.293 -6.467 0.000

2 6.508 8.452 4.532 3.977 0.000

3 -0.714 -2.290 1.437 -4.929 0.000

4 -0.026 3.230 0.146 5.417 0.000

5 -3.477 -10.194 -3.284 -8.911 0.000

MODEL RESULTS

Two-Tailed

Estimate S.E. Est./S.E. P-Value

Latent Class 1

Thresholds

PROTCHLD$1 -6.839 1.231 -5.557 0.000

PROTCHLD$2 -5.654 0.631 -8.965 0.000

PROTCHLD$3 -4.110 0.415 -9.899 0.000

PROTCHLD$4 0.625 0.212 2.954 0.003

DRRECVAX$1 -7.721 2.291 -3.370 0.001

DRRECVAX$2 -3.798 0.316 -12.000 0.000

DRRECVAX$3 -1.865 0.156 -11.949 0.000

DRRECVAX$4 1.901 0.235 8.102 0.000

NEWVAXSF$1 -5.997 0.986 -6.085 0.000

NEWVAXSF$2 -3.824 0.322 -11.858 0.000

NEWVAXSF$3 -1.826 0.184 -9.935 0.000

NEWVAXSF$4 1.461 0.159 9.179 0.000

VAXACHV$1 -6.926 0.741 -9.344 0.000

VAXACHV$2 -5.137 0.534 -9.623 0.000

VAXACHV$3 -2.906 0.300 -9.692 0.000

VAXACHV$4 0.819 0.225 3.631 0.000

CNCSDEFF$1 -3.809 0.496 -7.683 0.000

CNCSDEFF$2 -0.762 0.170 -4.482 0.000

CNCSDEFF$3 0.668 0.154 4.345 0.000

CNCSDEFF$4 3.821 0.324 11.783 0.000

VAXAUT$1 -6.809 1.326 -5.135 0.000

VAXAUT$2 -2.579 0.284 -9.078 0.000

VAXAUT$3 0.209 0.155 1.348 0.178

VAXAUT$4 1.795 0.185 9.685 0.000

PRNTREFU$1 -3.882 0.401 -9.679 0.000

PRNTREFU$2 -0.679 0.139 -4.883 0.000

PRNTREFU$3 0.364 0.148 2.466 0.014

PRNTREFU$4 3.102 0.260 11.915 0.000

Latent Class 2

Thresholds

PROTCHLD$1 -5.271 1.292 -4.081 0.000

PROTCHLD$2 -3.171 0.479 -6.623 0.000

PROTCHLD$3 -0.152 0.315 -0.481 0.630

PROTCHLD$4 2.891 0.408 7.081 0.000

DRRECVAX$1 -4.353 0.580 -7.500 0.000

DRRECVAX$2 -1.394 0.265 -5.268 0.000

DRRECVAX$3 0.556 0.317 1.752 0.080

DRRECVAX$4 3.320 0.454 7.318 0.000

NEWVAXSF$1 -4.119 0.925 -4.453 0.000

NEWVAXSF$2 -1.615 0.291 -5.544 0.000

NEWVAXSF$3 0.354 0.215 1.642 0.101

NEWVAXSF$4 1.442 0.207 6.954 0.000

VAXACHV$1 -5.036 0.719 -7.000 0.000

VAXACHV$2 -2.178 0.342 -6.368 0.000

VAXACHV$3 0.357 0.308 1.161 0.246

VAXACHV$4 2.726 0.339 8.032 0.000

CNCSDEFF$1 -0.962 0.261 -3.692 0.000

CNCSDEFF$2 0.701 0.164 4.262 0.000

CNCSDEFF$3 3.290 0.372 8.854 0.000

CNCSDEFF$4 5.563 1.003 5.546 0.000

VAXAUT$1 -3.177 0.478 -6.648 0.000

VAXAUT$2 -1.047 0.171 -6.107 0.000

VAXAUT$3 2.125 0.222 9.590 0.000

VAXAUT$4 3.989 0.552 7.223 0.000

PRNTREFU$1 -0.973 0.327 -2.977 0.003

PRNTREFU$2 0.635 0.178 3.568 0.000

PRNTREFU$3 2.829 0.395 7.155 0.000

PRNTREFU$4 4.669 0.709 6.588 0.000

Latent Class 3

Thresholds

PROTCHLD$1 -4.840 0.740 -6.544 0.000

PROTCHLD$2 -3.935 0.465 -8.468 0.000

PROTCHLD$3 -3.004 0.338 -8.900 0.000

PROTCHLD$4 -1.740 0.238 -7.303 0.000

DRRECVAX$1 -3.687 0.717 -5.142 0.000

DRRECVAX$2 -3.155 0.420 -7.518 0.000

DRRECVAX$3 -1.760 0.244 -7.222 0.000

DRRECVAX$4 -0.653 0.189 -3.457 0.001

NEWVAXSF$1 -4.993 1.072 -4.657 0.000

NEWVAXSF$2 -4.069 0.618 -6.584 0.000

NEWVAXSF$3 -3.373 0.727 -4.638 0.000

NEWVAXSF$4 -1.306 0.386 -3.379 0.001

VAXACHV$1 -15.000 0.000 999.000 999.000

VAXACHV$2 -6.060 2.204 -2.750 0.006

VAXACHV$3 -2.691 0.476 -5.653 0.000

VAXACHV$4 -1.809 0.344 -5.255 0.000

CNCSDEFF$1 -1.115 0.599 -1.862 0.063

CNCSDEFF$2 -0.211 0.454 -0.464 0.643

CNCSDEFF$3 1.616 0.832 1.943 0.052

CNCSDEFF$4 3.219 0.554 5.806 0.000

VAXAUT$1 -2.058 0.527 -3.904 0.000

VAXAUT$2 -1.480 0.480 -3.087 0.002

VAXAUT$3 1.248 0.558 2.237 0.025

VAXAUT$4 2.098 0.319 6.569 0.000

PRNTREFU$1 -0.899 0.567 -1.586 0.113

PRNTREFU$2 -0.143 0.384 -0.373 0.709

PRNTREFU$3 1.092 0.521 2.094 0.036

PRNTREFU$4 2.336 0.414 5.645 0.000

Latent Class 4

Thresholds

PROTCHLD$1 -0.125 0.396 -0.315 0.753

PROTCHLD$2 0.628 0.461 1.362 0.173

PROTCHLD$3 2.209 0.540 4.089 0.000

PROTCHLD$4 3.370 0.731 4.610 0.000

DRRECVAX$1 -0.150 0.386 -0.389 0.697

DRRECVAX$2 1.245 0.426 2.921 0.003

DRRECVAX$3 2.854 0.822 3.470 0.001

DRRECVAX$4 4.523 0.695 6.508 0.000

NEWVAXSF$1 -0.133 0.295 -0.450 0.652

NEWVAXSF$2 0.628 0.266 2.363 0.018

NEWVAXSF$3 1.170 0.321 3.643 0.000

NEWVAXSF$4 1.435 0.375 3.829 0.000

VAXACHV$1 -0.094 0.425 -0.222 0.824

VAXACHV$2 0.632 0.427 1.479 0.139

VAXACHV$3 1.970 0.590 3.340 0.001

VAXACHV$4 5.930 2.690 2.204 0.028

CNCSDEFF$1 1.198 0.268 4.477 0.000

CNCSDEFF$2 1.513 0.344 4.401 0.000

CNCSDEFF$3 1.602 0.351 4.568 0.000

CNCSDEFF$4 2.554 0.487 5.248 0.000

VAXAUT$1 0.112 0.339 0.331 0.741

VAXAUT$2 0.849 0.288 2.950 0.003

VAXAUT$3 1.929 0.331 5.822 0.000

VAXAUT$4 2.260 0.385 5.862 0.000

PRNTREFU$1 1.341 0.308 4.354 0.000

PRNTREFU$2 1.819 0.386 4.716 0.000

PRNTREFU$3 2.090 0.408 5.127 0.000

PRNTREFU$4 3.004 0.541 5.554 0.000

Latent Class 5

Thresholds

PROTCHLD$1 -4.799 0.352 -13.647 0.000

PROTCHLD$2 -4.502 0.373 -12.058 0.000

PROTCHLD$3 -3.966 0.302 -13.143 0.000

PROTCHLD$4 -2.896 0.195 -14.839 0.000

DRRECVAX$1 -5.543 0.399 -13.895 0.000

DRRECVAX$2 -4.546 0.429 -10.602 0.000

DRRECVAX$3 -3.105 0.209 -14.846 0.000

DRRECVAX$4 -1.449 0.114 -12.744 0.000

NEWVAXSF$1 -5.631 0.497 -11.327 0.000

NEWVAXSF$2 -5.029 0.359 -14.019 0.000

NEWVAXSF$3 -3.532 0.234 -15.119 0.000

NEWVAXSF$4 -1.243 0.105 -11.815 0.000

VAXACHV$1 -5.219 0.471 -11.086 0.000

VAXACHV$2 -4.972 0.421 -11.799 0.000

VAXACHV$3 -4.353 0.317 -13.728 0.000

VAXACHV$4 -2.331 0.129 -18.044 0.000

CNCSDEFF$1 -15.000 0.000 999.000 999.000

CNCSDEFF$2 -2.738 0.254 -10.762 0.000

CNCSDEFF$3 -1.564 0.271 -5.766 0.000

CNCSDEFF$4 0.208 0.181 1.145 0.252

VAXAUT$1 -6.455 1.502 -4.297 0.000

VAXAUT$2 -4.825 0.669 -7.211 0.000

VAXAUT$3 -1.938 0.398 -4.874 0.000

VAXAUT$4 -0.838 0.277 -3.027 0.002

PRNTREFU$1 -4.024 0.300 -13.430 0.000

PRNTREFU$2 -2.171 0.195 -11.158 0.000

PRNTREFU$3 -1.204 0.173 -6.961 0.000

PRNTREFU$4 0.427 0.122 3.509 0.000

Categorical Latent Variables

Means

C#1 -0.270 0.092 -2.933 0.003

C#2 -1.049 0.186 -5.626 0.000

C#3 -1.049 0.493 -2.128 0.033

C#4 -2.377 0.236 -10.061 0.000

RESULTS IN PROBABILITY SCALE

Latent Class 1

PROTCHLD

Category 1 0.001 0.001 0.813 0.416

Category 2 0.002 0.002 1.375 0.169

Category 3 0.013 0.006 1.968 0.049

Category 4 0.635 0.045 14.071 0.000

Category 5 0.349 0.048 7.255 0.000

DRRECVAX

Category 1 0.000 0.001 0.437 0.662

Category 2 0.021 0.007 3.248 0.001

Category 3 0.112 0.016 6.947 0.000

Category 4 0.736 0.026 28.504 0.000

Category 5 0.130 0.027 4.900 0.000

NEWVAXSF

Category 1 0.002 0.002 1.017 0.309

Category 2 0.019 0.006 2.923 0.003

Category 3 0.117 0.020 5.906 0.000

Category 4 0.673 0.025 26.766 0.000

Category 5 0.188 0.024 7.739 0.000

VAXACHV

Category 1 0.001 0.001 1.350 0.177

Category 2 0.005 0.003 1.627 0.104

Category 3 0.046 0.014 3.175 0.001

Category 4 0.642 0.042 15.434 0.000

Category 5 0.306 0.048 6.391 0.000

CNCSDEFF

Category 1 0.022 0.011 2.061 0.039

Category 2 0.297 0.030 9.768 0.000

Category 3 0.343 0.020 17.447 0.000

Category 4 0.317 0.031 10.209 0.000

Category 5 0.021 0.007 3.152 0.002

VAXAUT

Category 1 0.001 0.001 0.755 0.450

Category 2 0.069 0.018 3.794 0.000

Category 3 0.481 0.028 17.267 0.000

Category 4 0.306 0.023 13.162 0.000

Category 5 0.143 0.023 6.293 0.000

PRNTREFU

Category 1 0.020 0.008 2.545 0.011

Category 2 0.316 0.030 10.697 0.000

Category 3 0.254 0.019 13.581 0.000

Category 4 0.367 0.030 12.072 0.000

Category 5 0.043 0.011 4.014 0.000

Latent Class 2

PROTCHLD

Category 1 0.005 0.007 0.778 0.436

Category 2 0.035 0.015 2.372 0.018

Category 3 0.422 0.067 6.289 0.000

Category 4 0.485 0.079 6.127 0.000

Category 5 0.053 0.020 2.586 0.010

DRRECVAX

Category 1 0.013 0.007 1.745 0.081

Category 2 0.186 0.040 4.679 0.000

Category 3 0.437 0.047 9.381 0.000

Category 4 0.330 0.074 4.460 0.000

Category 5 0.035 0.015 2.284 0.022

NEWVAXSF

Category 1 0.016 0.015 1.099 0.272

Category 2 0.150 0.031 4.826 0.000

Category 3 0.422 0.035 11.903 0.000

Category 4 0.221 0.057 3.898 0.000

Category 5 0.191 0.032 5.964 0.000

VAXACHV

Category 1 0.006 0.005 1.399 0.162

Category 2 0.095 0.030 3.212 0.001

Category 3 0.487 0.056 8.628 0.000

Category 4 0.350 0.075 4.694 0.000

Category 5 0.061 0.020 3.139 0.002

CNCSDEFF

Category 1 0.276 0.052 5.303 0.000

Category 2 0.392 0.044 8.869 0.000

Category 3 0.296 0.036 8.312 0.000

Category 4 0.032 0.012 2.691 0.007

Category 5 0.004 0.004 1.001 0.317

VAXAUT

Category 1 0.040 0.018 2.180 0.029

Category 2 0.220 0.027 8.073 0.000

Category 3 0.634 0.033 19.152 0.000

Category 4 0.088 0.019 4.678 0.000

Category 5 0.018 0.010 1.844 0.065

PRNTREFU

Category 1 0.274 0.065 4.216 0.000

Category 2 0.379 0.049 7.806 0.000

Category 3 0.290 0.034 8.461 0.000

Category 4 0.047 0.020 2.322 0.020

Category 5 0.009 0.007 1.424 0.154

Latent Class 3

PROTCHLD

Category 1 0.008 0.006 1.363 0.173

Category 2 0.011 0.007 1.533 0.125

Category 3 0.028 0.013 2.178 0.029

Category 4 0.102 0.027 3.846 0.000

Category 5 0.851 0.030 28.111 0.000

DRRECVAX

Category 1 0.024 0.017 1.430 0.153

Category 2 0.016 0.014 1.193 0.233

Category 3 0.106 0.023 4.594 0.000

Category 4 0.195 0.038 5.208 0.000

Category 5 0.658 0.043 15.460 0.000

NEWVAXSF

Category 1 0.007 0.007 0.939 0.348

Category 2 0.010 0.007 1.470 0.141

Category 3 0.016 0.021 0.782 0.434

Category 4 0.180 0.055 3.263 0.001

Category 5 0.787 0.065 12.138 0.000

VAXACHV

Category 1 0.000 0.000 0.000 1.000

Category 2 0.002 0.005 0.455 0.649

Category 3 0.061 0.028 2.146 0.032

Category 4 0.077 0.026 3.009 0.003

Category 5 0.859 0.042 20.638 0.000

CNCSDEFF

Category 1 0.247 0.111 2.217 0.027

Category 2 0.201 0.034 5.850 0.000

Category 3 0.387 0.038 10.074 0.000

Category 4 0.127 0.107 1.187 0.235

Category 5 0.038 0.021 1.876 0.061

VAXAUT

Category 1 0.113 0.053 2.139 0.032

Category 2 0.072 0.026 2.753 0.006

Category 3 0.592 0.044 13.425 0.000

Category 4 0.114 0.079 1.440 0.150

Category 5 0.109 0.031 3.515 0.000

PRNTREFU

Category 1 0.289 0.117 2.480 0.013

Category 2 0.175 0.037 4.687 0.000

Category 3 0.284 0.032 8.951 0.000

Category 4 0.163 0.073 2.228 0.026

Category 5 0.088 0.033 2.650 0.008

Latent Class 4

PROTCHLD

Category 1 0.469 0.099 4.748 0.000

Category 2 0.183 0.044 4.191 0.000

Category 3 0.249 0.083 3.000 0.003

Category 4 0.066 0.039 1.680 0.093

Category 5 0.033 0.023 1.415 0.157

DRRECVAX

Category 1 0.462 0.096 4.817 0.000

Category 2 0.314 0.062 5.041 0.000

Category 3 0.169 0.063 2.676 0.007

Category 4 0.044 0.041 1.057 0.290

Category 5 0.011 0.007 1.455 0.146

NEWVAXSF

Category 1 0.467 0.073 6.351 0.000

Category 2 0.185 0.051 3.636 0.000

Category 3 0.111 0.042 2.672 0.008

Category 4 0.045 0.019 2.397 0.017

Category 5 0.192 0.058 3.303 0.001

VAXACHV

Category 1 0.476 0.106 4.498 0.000

Category 2 0.176 0.050 3.545 0.000

Category 3 0.225 0.075 2.989 0.003

Category 4 0.120 0.063 1.893 0.058

Category 5 0.003 0.007 0.373 0.709

CNCSDEFF

Category 1 0.768 0.048 16.120 0.000

Category 2 0.051 0.030 1.728 0.084

Category 3 0.013 0.011 1.219 0.223

Category 4 0.096 0.033 2.927 0.003

Category 5 0.072 0.033 2.215 0.027

VAXAUT

Category 1 0.528 0.085 6.244 0.000

Category 2 0.172 0.055 3.135 0.002

Category 3 0.173 0.062 2.797 0.005

Category 4 0.032 0.014 2.265 0.024

Category 5 0.095 0.033 2.865 0.004

PRNTREFU

Category 1 0.793 0.051 15.658 0.000

Category 2 0.068 0.037 1.845 0.065

Category 3 0.030 0.017 1.788 0.074

Category 4 0.063 0.028 2.220 0.026

Category 5 0.047 0.024 1.941 0.052

Latent Class 5

PROTCHLD

Category 1 0.008 0.003 2.867 0.004

Category 2 0.003 0.003 0.877 0.380

Category 3 0.008 0.003 2.424 0.015

Category 4 0.034 0.007 4.600 0.000

Category 5 0.948 0.010 97.851 0.000

DRRECVAX

Category 1 0.004 0.002 2.517 0.012

Category 2 0.007 0.004 1.488 0.137

Category 3 0.032 0.006 5.043 0.000

Category 4 0.147 0.014 10.423 0.000

Category 5 0.810 0.018 46.254 0.000

NEWVAXSF

Category 1 0.004 0.002 2.019 0.044

Category 2 0.003 0.001 1.969 0.049

Category 3 0.022 0.006 3.729 0.000

Category 4 0.196 0.016 12.125 0.000

Category 5 0.776 0.018 42.451 0.000

VAXACHV

Category 1 0.005 0.003 2.136 0.033

Category 2 0.001 0.001 1.003 0.316

Category 3 0.006 0.003 2.144 0.032

Category 4 0.076 0.010 7.675 0.000

Category 5 0.911 0.010 87.383 0.000

CNCSDEFF

Category 1 0.000 0.000 0.000 1.000

Category 2 0.061 0.015 4.185 0.000

Category 3 0.112 0.028 4.032 0.000

Category 4 0.379 0.017 22.417 0.000

Category 5 0.448 0.045 10.001 0.000

VAXAUT

Category 1 0.002 0.002 0.667 0.505

Category 2 0.006 0.004 1.515 0.130

Category 3 0.118 0.041 2.880 0.004

Category 4 0.176 0.020 8.869 0.000

Category 5 0.698 0.058 11.964 0.000

PRNTREFU

Category 1 0.018 0.005 3.397 0.001

Category 2 0.085 0.016 5.464 0.000

Category 3 0.128 0.017 7.540 0.000

Category 4 0.374 0.016 23.870 0.000

Category 5 0.395 0.029 13.592 0.000

LATENT CLASS ODDS RATIO RESULTS

95% C.I.

Estimate S.E. Lower 2.5% Upper 2.5%

Latent Class 1 Compared to Latent Class 2

PROTCHLD

Category > 1 4.795 9.296 0.107 214.217

Category > 2 11.976 10.356 2.199 65.224

Category > 3 52.387 18.371 26.346 104.165

Category > 4 9.635 4.571 3.802 24.414

DRRECVAX

Category > 1 29.030 68.969 0.276 3055.916

Category > 2 11.061 4.332 5.134 23.831

Category > 3 11.254 3.137 6.517 19.434

Category > 4 4.136 2.200 1.458 11.734

NEWVAXSF

Category > 1 6.535 9.408 0.389 109.818

Category > 2 9.106 3.735 4.076 20.347

Category > 3 8.848 1.772 5.975 13.102

Category > 4 0.981 0.290 0.549 1.751

VAXACHV

Category > 1 6.619 7.186 0.788 55.580

Category > 2 19.274 12.968 5.155 72.062

Category > 3 26.145 7.019 15.447 44.251

Category > 4 6.736 2.827 2.959 15.335

CNCSDEFF

Category > 1 17.243 7.675 7.206 41.258

Category > 2 4.319 0.975 2.774 6.722

Category > 3 13.755 5.793 6.025 31.404

Category > 4 5.711 6.433 0.628 51.945

VAXAUT

Category > 1 37.782 53.663 2.335 611.362

Category > 2 4.630 1.428 2.530 8.473

Category > 3 6.800 1.702 4.163 11.107

Category > 4 8.978 5.417 2.752 29.294

PRNTREFU

Category > 1 18.335 8.987 7.016 47.917

Category > 2 3.723 0.739 2.522 5.495

Category > 3 11.753 4.373 5.668 24.370

Category > 4 4.792 3.730 1.042 22.032

Latent Class 1 Compared to Latent Class 3

PROTCHLD

Category > 1 7.382 10.970 0.401 135.862

Category > 2 5.578 4.537 1.133 27.466

Category > 3 3.021 1.755 0.968 9.434

Category > 4 0.094 0.031 0.049 0.181

DRRECVAX

Category > 1 56.518 137.402 0.482 6631.449

Category > 2 1.902 1.045 0.648 5.581

Category > 3 1.111 0.330 0.621 1.987

Category > 4 0.078 0.022 0.044 0.137

NEWVAXSF

Category > 1 2.729 4.139 0.140 53.328

Category > 2 0.782 0.587 0.180 3.401

Category > 3 0.213 0.168 0.045 0.999

Category > 4 0.063 0.021 0.033 0.121

VAXACHV

Category > 1 0.000 0.000 0.000 0.000

Category > 2 0.397 0.931 0.004 39.245

Category > 3 1.240 0.737 0.387 3.977

Category > 4 0.072 0.033 0.029 0.179

CNCSDEFF

Category > 1 14.792 11.177 3.364 65.043

Category > 2 1.736 0.846 0.668 4.513

Category > 3 2.579 2.142 0.506 13.137

Category > 4 0.548 0.346 0.159 1.886

VAXAUT

Category > 1 115.642 141.427 10.522 1270.965

Category > 2 3.001 1.703 0.987 9.128

Category > 3 2.829 1.584 0.944 8.479

Category > 4 1.355 0.488 0.669 2.744

PRNTREFU

Category > 1 19.733 11.831 6.093 63.908

Category > 2 1.709 0.702 0.764 3.823

Category > 3 2.069 1.100 0.730 5.866

Category > 4 0.465 0.228 0.178 1.216

Latent Class 1 Compared to Latent Class 4

PROTCHLD

Category > 1 823.840 1068.001 64.916 10455.255

Category > 2 534.793 427.614 111.574 2563.356

Category > 3 555.373 333.561 171.134 1802.319

Category > 4 15.565 12.055 3.411 71.027

DRRECVAX

Category > 1 1941.085 4330.907 24.481 *********

Category > 2 154.975 77.132 58.426 411.069

Category > 3 112.029 93.029 22.003 570.399

Category > 4 13.763 9.835 3.392 55.846

NEWVAXSF

Category > 1 351.975 366.019 45.849 2702.058

Category > 2 85.747 34.438 39.026 188.401

Category > 3 20.005 6.979 10.097 39.637

Category > 4 0.975 0.390 0.445 2.135

VAXACHV

Category > 1 926.906 815.416 165.274 5198.368

Category > 2 319.973 217.502 84.430 1212.631

Category > 3 131.092 83.812 37.442 458.976

Category > 4 165.931 456.353 0.757 36390.770

CNCSDEFF

Category > 1 149.559 83.898 49.809 449.078

Category > 2 9.724 4.122 4.237 22.320

Category > 3 2.544 1.084 1.104 5.862

Category > 4 0.282 0.177 0.082 0.967

VAXAUT

Category > 1 1013.647 1335.513 76.627 13408.895

Category > 2 30.835 10.698 15.621 60.866

Category > 3 5.588 2.234 2.552 12.236

Category > 4 1.592 0.743 0.637 3.976

PRNTREFU

Category > 1 185.526 94.804 68.144 505.103

Category > 2 12.153 5.432 5.061 29.185

Category > 3 5.617 2.684 2.202 14.329

Category > 4 0.906 0.578 0.260 3.166

Latent Class 1 Compared to Latent Class 5

PROTCHLD

Category > 1 7.692 9.926 0.613 96.479

Category > 2 3.164 2.481 0.681 14.712

Category > 3 1.155 0.607 0.413 3.236

Category > 4 0.030 0.008 0.017 0.050

DRRECVAX

Category > 1 8.832 20.760 0.088 884.670

Category > 2 0.473 0.261 0.160 1.397

Category > 3 0.289 0.072 0.178 0.470

Category > 4 0.035 0.008 0.022 0.055

NEWVAXSF

Category > 1 1.441 1.708 0.141 14.713

Category > 2 0.300 0.151 0.112 0.802

Category > 3 0.182 0.051 0.105 0.314

Category > 4 0.067 0.012 0.047 0.096

VAXACHV

Category > 1 5.514 5.087 0.904 33.638

Category > 2 1.179 0.837 0.293 4.737

Category > 3 0.235 0.103 0.100 0.556

Category > 4 0.043 0.009 0.029 0.064

CNCSDEFF

Category > 1 0.000 0.000 0.000 0.000

Category > 2 0.139 0.039 0.080 0.241

Category > 3 0.107 0.030 0.062 0.185

Category > 4 0.027 0.009 0.014 0.051

VAXAUT

Category > 1 1.425 2.585 0.041 49.873

Category > 2 0.106 0.077 0.025 0.441

Category > 3 0.117 0.046 0.054 0.254

Category > 4 0.072 0.021 0.041 0.127

PRNTREFU

Category > 1 0.867 0.407 0.346 2.174

Category > 2 0.225 0.050 0.145 0.348

Category > 3 0.208 0.043 0.139 0.313

Category > 4 0.069 0.019 0.040 0.118

Latent Class 2 Compared to Latent Class 3

PROTCHLD

Category > 1 1.539 2.379 0.074 31.814

Category > 2 0.466 0.324 0.119 1.818

Category > 3 0.058 0.028 0.022 0.151

Category > 4 0.010 0.004 0.004 0.024

DRRECVAX

Category > 1 1.947 1.928 0.280 13.555

Category > 2 0.172 0.089 0.062 0.473

Category > 3 0.099 0.041 0.044 0.222

Category > 4 0.019 0.009 0.007 0.049

NEWVAXSF

Category > 1 0.418 0.615 0.023 7.487

Category > 2 0.086 0.060 0.022 0.340

Category > 3 0.024 0.019 0.005 0.115

Category > 4 0.064 0.030 0.026 0.160

VAXACHV

Category > 1 0.000 0.000 0.000 0.000

Category > 2 0.021 0.047 0.000 1.829

Category > 3 0.047 0.028 0.015 0.149

Category > 4 0.011 0.005 0.004 0.026

CNCSDEFF

Category > 1 0.858 0.581 0.228 3.232

Category > 2 0.402 0.200 0.152 1.064

Category > 3 0.187 0.175 0.030 1.168

Category > 4 0.096 0.112 0.010 0.943

VAXAUT

Category > 1 3.061 2.284 0.709 13.211

Category > 2 0.648 0.334 0.236 1.777

Category > 3 0.416 0.256 0.124 1.390

Category > 4 0.151 0.097 0.043 0.532

PRNTREFU

Category > 1 1.076 0.735 0.282 4.104

Category > 2 0.459 0.203 0.193 1.092

Category > 3 0.176 0.121 0.046 0.673

Category > 4 0.097 0.085 0.017 0.545

Latent Class 2 Compared to Latent Class 4

PROTCHLD

Category > 1 171.797 208.760 15.873 1859.416

Category > 2 44.654 23.178 16.144 123.507

Category > 3 10.601 5.906 3.557 31.595

Category > 4 1.616 1.393 0.298 8.754

DRRECVAX

Category > 1 66.864 40.387 20.467 218.443

Category > 2 14.011 5.536 6.459 30.393

Category > 3 9.955 8.811 1.757 56.418

Category > 4 3.328 3.054 0.551 20.105

NEWVAXSF

Category > 1 53.862 46.255 10.007 289.921

Category > 2 9.416 3.353 4.686 18.922

Category > 3 2.261 0.870 1.064 4.805

Category > 4 0.994 0.482 0.384 2.573

VAXACHV

Category > 1 140.028 104.393 32.480 603.687

Category > 2 16.601 7.357 6.965 39.568

Category > 3 5.014 3.343 1.357 18.526

Category > 4 24.634 66.778 0.121 5000.585

CNCSDEFF

Category > 1 8.674 3.436 3.990 18.856

Category > 2 2.252 0.951 0.984 5.154

Category > 3 0.185 0.099 0.065 0.528

Category > 4 0.049 0.055 0.006 0.436

VAXAUT

Category > 1 26.829 13.044 10.345 69.575

Category > 2 6.660 2.081 3.609 12.288

Category > 3 0.822 0.359 0.349 1.936

Category > 4 0.177 0.122 0.046 0.684

PRNTREFU

Category > 1 10.119 5.098 3.770 27.162

Category > 2 3.265 1.581 1.264 8.433

Category > 3 0.478 0.304 0.137 1.666

Category > 4 0.189 0.170 0.033 1.098

Latent Class 2 Compared to Latent Class 5

PROTCHLD

Category > 1 1.604 2.163 0.114 22.539

Category > 2 0.264 0.159 0.081 0.862

Category > 3 0.022 0.010 0.009 0.052

Category > 4 0.003 0.001 0.001 0.007

DRRECVAX

Category > 1 0.304 0.216 0.076 1.226

Category > 2 0.043 0.021 0.017 0.110

Category > 3 0.026 0.009 0.013 0.052

Category > 4 0.008 0.004 0.003 0.021

NEWVAXSF

Category > 1 0.221 0.230 0.028 1.710

Category > 2 0.033 0.015 0.014 0.078

Category > 3 0.021 0.006 0.012 0.036

Category > 4 0.068 0.016 0.043 0.109

VAXACHV

Category > 1 0.833 0.718 0.154 4.513

Category > 2 0.061 0.033 0.021 0.177

Category > 3 0.009 0.004 0.004 0.021

Category > 4 0.006 0.002 0.003 0.013

CNCSDEFF

Category > 1 0.000 0.000 0.000 0.000

Category > 2 0.032 0.010 0.018 0.058

Category > 3 0.008 0.004 0.003 0.020

Category > 4 0.005 0.005 0.001 0.036

VAXAUT

Category > 1 0.038 0.060 0.002 0.865

Category > 2 0.023 0.016 0.006 0.089

Category > 3 0.017 0.008 0.007 0.043

Category > 4 0.008 0.005 0.002 0.027

PRNTREFU

Category > 1 0.047 0.021 0.020 0.114

Category > 2 0.060 0.016 0.036 0.101

Category > 3 0.018 0.008 0.008 0.042

Category > 4 0.014 0.010 0.003 0.060

Latent Class 3 Compared to Latent Class 4

PROTCHLD

Category > 1 111.598 94.014 21.407 581.773

Category > 2 95.878 63.517 26.170 351.264

Category > 3 183.808 119.202 51.563 655.222

Category > 4 165.738 124.180 38.163 719.784

DRRECVAX

Category > 1 34.345 28.044 6.931 170.186

Category > 2 81.499 49.946 24.518 270.904

Category > 3 100.841 86.075 18.926 537.293

Category > 4 177.000 127.367 43.196 725.271

NEWVAXSF

Category > 1 128.970 143.521 14.562 1142.211

Category > 2 109.623 73.171 29.630 405.575

Category > 3 93.895 76.208 19.132 460.804

Category > 4 15.504 8.555 5.257 45.721

VAXACHV

Category > 1 ********* 0.000 ********* *********

Category > 2 805.760 1832.478 9.340 69512.672

Category > 3 105.729 79.722 24.119 463.479

Category > 4 2296.472 6145.674 12.108 *********

CNCSDEFF

Category > 1 10.111 6.659 2.781 36.760

Category > 2 5.602 3.133 1.871 16.766

Category > 3 0.986 0.887 0.169 5.743

Category > 4 0.514 0.380 0.121 2.186

VAXAUT

Category > 1 8.765 5.502 2.562 29.994

Category > 2 10.275 5.665 3.487 30.278

Category > 3 1.976 1.229 0.584 6.684

Category > 4 1.175 0.585 0.443 3.116

PRNTREFU

Category > 1 9.402 6.012 2.685 32.924

Category > 2 7.113 3.768 2.519 20.087

Category > 3 2.714 1.767 0.758 9.725

Category > 4 1.950 1.328 0.513 7.407

Latent Class 3 Compared to Latent Class 5

PROTCHLD

Category > 1 1.042 0.916 0.186 5.840

Category > 2 0.567 0.365 0.161 2.005

Category > 3 0.382 0.181 0.151 0.967

Category > 4 0.315 0.093 0.177 0.562

DRRECVAX

Category > 1 0.156 0.142 0.026 0.925

Category > 2 0.249 0.149 0.077 0.808

Category > 3 0.260 0.075 0.148 0.459

Category > 4 0.451 0.099 0.293 0.694

NEWVAXSF

Category > 1 0.528 0.649 0.047 5.871

Category > 2 0.383 0.284 0.089 1.641

Category > 3 0.852 0.706 0.168 4.319

Category > 4 1.065 0.447 0.467 2.426

VAXACHV

Category > 1 17695.943 0.000 17695.943 17695.943

Category > 2 2.969 6.814 0.033 266.811

Category > 3 0.190 0.114 0.059 0.614

Category > 4 0.593 0.235 0.273 1.289

CNCSDEFF

Category > 1 0.000 0.000 0.000 0.000

Category > 2 0.080 0.028 0.040 0.160

Category > 3 0.042 0.026 0.012 0.140

Category > 4 0.049 0.026 0.017 0.140

VAXAUT

Category > 1 0.012 0.017 0.001 0.189

Category > 2 0.035 0.023 0.010 0.128

Category > 3 0.041 0.013 0.022 0.076

Category > 4 0.053 0.017 0.029 0.098

PRNTREFU

Category > 1 0.044 0.023 0.016 0.123

Category > 2 0.132 0.038 0.075 0.231

Category > 3 0.101 0.040 0.046 0.219

Category > 4 0.148 0.053 0.073 0.300

Latent Class 4 Compared to Latent Class 5

PROTCHLD

Category > 1 0.009 0.005 0.003 0.027

Category > 2 0.006 0.004 0.002 0.019

Category > 3 0.002 0.001 0.001 0.007

Category > 4 0.002 0.001 0.000 0.008

DRRECVAX

Category > 1 0.005 0.003 0.001 0.015

Category > 2 0.003 0.002 0.001 0.010

Category > 3 0.003 0.002 0.000 0.013

Category > 4 0.003 0.002 0.001 0.010

NEWVAXSF

Category > 1 0.004 0.002 0.001 0.013

Category > 2 0.003 0.002 0.001 0.008

Category > 3 0.009 0.004 0.004 0.020

Category > 4 0.069 0.026 0.033 0.144

VAXACHV

Category > 1 0.006 0.004 0.002 0.021

Category > 2 0.004 0.002 0.001 0.012

Category > 3 0.002 0.001 0.000 0.007

Category > 4 0.000 0.001 0.000 0.052

CNCSDEFF

Category > 1 0.000 0.000 0.000 0.000

Category > 2 0.014 0.006 0.006 0.034

Category > 3 0.042 0.019 0.017 0.103

Category > 4 0.096 0.051 0.034 0.270

VAXAUT

Category > 1 0.001 0.002 0.000 0.029

Category > 2 0.003 0.002 0.001 0.014

Category > 3 0.021 0.010 0.008 0.055

Category > 4 0.045 0.021 0.018 0.111

PRNTREFU

Category > 1 0.005 0.002 0.002 0.011

Category > 2 0.019 0.008 0.008 0.044

Category > 3 0.037 0.016 0.016 0.089

Category > 4 0.076 0.042 0.025 0.227

QUALITY OF NUMERICAL RESULTS

Condition Number for the Information Matrix 0.463E-04

(ratio of smallest to largest eigenvalue)

TECHNICAL 11 OUTPUT

Random Starts Specifications for the k-1 Class Analysis Model

Number of initial stage random starts 250

Number of final stage optimizations 250

VUONG-LO-MENDELL-RUBIN LIKELIHOOD RATIO TEST FOR 4 (H0) VERSUS 5 CLASSES

H0 Loglikelihood Value -34652.973

2 Times the Loglikelihood Difference 812.469

Difference in the Number of Parameters 29

Mean 290.627

Standard Deviation 500.831

P-Value 0.1487

LO-MENDELL-RUBIN ADJUSTED LRT TEST

Value 809.156

P-Value 0.1503

DIAGRAM INFORMATION

Mplus diagrams are currently not available for Mixture analysis.

No diagram output was produced.

Beginning Time: 19:09:19

Ending Time: 19:11:21

Elapsed Time: 00:02:02

MUTHEN & MUTHEN

3463 Stoner Ave.

Los Angeles, CA 90066

Tel: (310) 391-9971

Fax: (310) 391-8971

Web: www.StatModel.com

Support: Support@StatModel.com

Copyright (c) 1998-2020 Muthen & Muthen

# Canada

Mplus VERSION 8.5

MUTHEN & MUTHEN

06/15/2021 7:06 PM

INPUT INSTRUCTIONS

Title:

Stata2Mplus conversion for C:\Users\...\

List of variables converted shown below

RespondentID :

weight :

ProtChld : Getting vaccines is a good way to protect children from disease.

1: Strongly agree

2: Agree

3: Neither agree nor disagree

4: Disagree

5: Strongly disagree

DrRecVax : Generally, I do what my doctor recommends about vaccines.

1: Strongly agree

2: Agree

3: Neither agree nor disagree

4: Disagree

5: Strongly disagree

NewVaxSf : New vaccines are recommended only if they are safe.

1: Strongly agree

2: Agree

3: Neither agree nor disagree

4: Disagree

5: Strongly disagree

UnvxChld : Enough children are vaccinated that even unvaccinated children are safe from

1: Strongly agree

2: Agree

3: Neither agree nor disagree

4: Disagree

5: Strongly disagree

CncSdEff : I am concerned about serious side effects of vaccines.

1: Strongly agree

2: Agree

3: Neither agree nor disagree

4: Disagree

5: Strongly disagree

VaxAut : Some vaccines cause autism in healthy children.

1: Strongly agree

2: Agree

3: Neither agree nor disagree

4: Disagree

5: Strongly disagree

PrntRefu : Parents should have the right to refuse vaccines required for schools for any

1: Strongly agree

2: Agree

3: Neither agree nor disagree

4: Disagree

5: Strongly disagree

VaxAchv : Vaccinations are one of the most significant achievements in improving public

1: Strongly agree

2: Agree

3: Neither agree nor disagree

4: Disagree

5: Strongly disagree

group :

id :

Data:

File is C:\...\combinedformplus.dat ;

Variable:

Names are

RespondentID weight ProtChld DrRecVax NewVaxSf UnvxChld CncSdEff VaxAut

PrntRefu VaxAchv group id;

Missing are all (-9999) ;

Usevariables are ProtChld DrRecVax NewVaxSf VaxAchv

CncSdEff VaxAut PrntRefu ;

Categorical are ProtChld DrRecVax NewVaxSf VaxAchv

CncSdEff VaxAut PrntRefu;

Weight=weight;

Idvariable =id;

classes= c(5);

Useobservations are group==1;

Missing are all (-9999) ;

Analysis:

Stseed=1234;

Type = mixture ;

Starts= 250 250;

Model:

! %Overall%

! c on canada uk;

Output:

Tech11;

Savedata:

!File is probability.csv;

!Save is CPROB;

*** WARNING

Input line exceeded 90 characters. Some input may be truncated.

Stata2Mplus conversion for C:\Users\...\a

*** WARNING

Input line exceeded 90 characters. Some input may be truncated.

UnvxChld : Enough children are vaccinated that even unvaccinated children are safe from d

*** WARNING

Input line exceeded 90 characters. Some input may be truncated.

VaxAchv : Vaccinations are one of the most significant achievements in improving public h

*** WARNING

Data set contains cases with missing on all variables.

These cases were not included in the analysis.

Number of cases with missing on all variables: 1

4 WARNING(S) FOUND IN THE INPUT INSTRUCTIONS

Stata2Mplus conversion for C:\Users\...\

List of variables converted shown below

RespondentID :

weight :

ProtChld : Getting vaccines is a good way to protect children from disease.

1: Strongly agree

2: Agree

3: Neither agree nor disagree

4: Disagree

5: Strongly disagree

DrRecVax : Generally, I do what my doctor recommends about vaccines.

1: Strongly agree

2: Agree

3: Neither agree nor disagree

4: Disagree

5: Strongly disagree

NewVaxSf : New vaccines are recommended only if they are safe.

1: Strongly agree

2: Agree

3: Neither agree nor disagree

4: Disagree

5: Strongly disagree

UnvxChld : Enough children are vaccinated that even unvaccinated children are safe from

1: Strongly agree

2: Agree

3: Neither agree nor disagree

4: Disagree

5: Strongly disagree

CncSdEff : I am concerned about serious side effects of vaccines.

1: Strongly agree

2: Agree

3: Neither agree nor disagree

4: Disagree

5: Strongly disagree

VaxAut : Some vaccines cause autism in healthy children.

1: Strongly agree

2: Agree

3: Neither agree nor disagree

4: Disagree

5: Strongly disagree

PrntRefu : Parents should have the right to refuse vaccines required for schools for any

1: Strongly agree

2: Agree

3: Neither agree nor disagree

4: Disagree

5: Strongly disagree

VaxAchv : Vaccinations are one of the most significant achievements in improving public

1: Strongly agree

2: Agree

3: Neither agree nor disagree

4: Disagree

5: Strongly disagree

group :

id :

SUMMARY OF ANALYSIS

Number of groups 1

Number of observations 4089

Number of dependent variables 7

Number of independent variables 0

Number of continuous latent variables 0

Number of categorical latent variables 1

Observed dependent variables

Binary and ordered categorical (ordinal)

PROTCHLD DRRECVAX NEWVAXSF VAXACHV CNCSDEFF VAXAUT

PRNTREFU

Categorical latent variables

C

Variables with special functions

Weight variable WEIGHT

ID variable ID

Estimator MLR

Information matrix OBSERVED

Optimization Specifications for the Quasi-Newton Algorithm for

Continuous Outcomes

Maximum number of iterations 100

Convergence criterion 0.100D-05

Optimization Specifications for the EM Algorithm

Maximum number of iterations 500

Convergence criteria

Loglikelihood change 0.100D-06

Relative loglikelihood change 0.100D-06

Derivative 0.100D-05

Optimization Specifications for the M step of the EM Algorithm for

Categorical Latent variables

Number of M step iterations 1

M step convergence criterion 0.100D-05

Basis for M step termination ITERATION

Optimization Specifications for the M step of the EM Algorithm for

Censored, Binary or Ordered Categorical (Ordinal), Unordered

Categorical (Nominal) and Count Outcomes

Number of M step iterations 1

M step convergence criterion 0.100D-05

Basis for M step termination ITERATION

Maximum value for logit thresholds 15

Minimum value for logit thresholds -15

Minimum expected cell size for chi-square 0.100D-01

Maximum number of iterations for H1 2000

Convergence criterion for H1 0.100D-03

Optimization algorithm EMA

Random Starts Specifications

Number of initial stage random starts 250

Number of final stage optimizations 250

Number of initial stage iterations 10

Initial stage convergence criterion 0.100D+01

Random starts scale 0.500D+01

Random seed for generating random starts 1234

Link LOGIT

Input data file(s)

C:\...\

Input data format FREE

SUMMARY OF DATA

Number of missing data patterns 19

Number of y missing data patterns 0

Number of u missing data patterns 19

COVARIANCE COVERAGE OF DATA

Minimum covariance coverage value 0.100

PROPORTION OF DATA PRESENT FOR U

Covariance Coverage

PROTCHLD DRRECVAX NEWVAXSF VAXACHV CNCSDEFF

________ ________ ________ ________ ________

PROTCHLD 0.995

DRRECVAX 0.992 0.997

NEWVAXSF 0.991 0.992 0.995

VAXACHV 0.993 0.994 0.993 0.997

CNCSDEFF 0.992 0.993 0.991 0.994 0.996

VAXAUT 0.991 0.992 0.990 0.993 0.991

PRNTREFU 0.993 0.994 0.993 0.995 0.994

Covariance Coverage

VAXAUT PRNTREFU

________ ________

VAXAUT 0.994

PRNTREFU 0.993 0.997

UNIVARIATE PROPORTIONS AND COUNTS FOR CATEGORICAL VARIABLES

PROTCHLD

Category 1 0.033 132.656

Category 2 0.031 125.229

Category 3 0.099 403.301

Category 4 0.299 1216.175

Category 5 0.538 2188.621

DRRECVAX

Category 1 0.032 129.981

Category 2 0.061 248.172

Category 3 0.146 596.210

Category 4 0.356 1450.479

Category 5 0.405 1652.639

NEWVAXSF

Category 1 0.053 214.362

Category 2 0.076 309.929

Category 3 0.152 617.901

Category 4 0.338 1375.364

Category 5 0.381 1550.957

VAXACHV

Category 1 0.038 154.302

Category 2 0.044 181.287

Category 3 0.130 528.154

Category 4 0.313 1277.740

Category 5 0.475 1935.448

CNCSDEFF

Category 1 0.202 821.710

Category 2 0.268 1089.567

Category 3 0.206 840.172

Category 4 0.196 798.556

Category 5 0.128 519.232

VAXAUT

Category 1 0.063 253.985

Category 2 0.087 352.390

Category 3 0.317 1286.590

Category 4 0.161 655.108

Category 5 0.373 1515.294

PRNTREFU

Category 1 0.156 637.022

Category 2 0.161 656.915

Category 3 0.172 700.317

Category 4 0.249 1014.226

Category 5 0.262 1068.328

RANDOM STARTS RESULTS RANKED FROM THE BEST TO THE WORST LOGLIKELIHOOD VALUES

Final stage loglikelihood values at local maxima, seeds, and initial stage start numbers:

-32457.836 749092 177

-32457.836 286672 75

-32457.836 138304 65

-32457.836 155285 235

-32457.836 789192 172

-32457.836 217388 11

-32457.836 559172 204

-32457.836 418410 101

-32457.836 283686 186

-32457.836 285532 120

-32457.836 723320 84

-32457.836 36496 173

-32457.836 528306 23

-32457.836 147827 139

-32457.836 699910 182

-32457.836 95220 111

-32457.836 515684 129

-32457.836 923515 198

-32457.836 717812 190

-32457.836 134946 209

-32457.836 98826 88

-32616.123 163636 238

-32616.123 232894 1

-32636.328 569230 44

-32636.328 779890 150

-32636.328 3016 222

Unperturbed starting value run did not converge or was rejected in the third stage.

223 perturbed starting value run(s) did not converge or were rejected in the third stage.

THE BEST LOGLIKELIHOOD VALUE HAS BEEN REPLICATED. RERUN WITH AT LEAST TWICE THE

RANDOM STARTS TO CHECK THAT THE BEST LOGLIKELIHOOD IS STILL OBTAINED AND REPLICATED.

IN THE OPTIMIZATION, ONE OR MORE LOGIT THRESHOLDS APPROACHED EXTREME VALUES

OF -15.000 AND 15.000 AND WERE FIXED TO STABILIZE MODEL ESTIMATION. THESE

VALUES IMPLY PROBABILITIES OF 0 AND 1. IN THE MODEL RESULTS SECTION, THESE

PARAMETERS HAVE 0 STANDARD ERRORS AND 999 IN THE Z-SCORE AND P-VALUE COLUMNS.

THE MODEL ESTIMATION TERMINATED NORMALLY

MODEL FIT INFORMATION

Number of Free Parameters 144

Loglikelihood

H0 Value -32457.836

H0 Scaling Correction Factor 1.6474

for MLR

Information Criteria

Akaike (AIC) 65203.673

Bayesian (BIC) 66113.185

Sample-Size Adjusted BIC 65655.616

(n* = (n + 2) / 24)

Chi-Square Test of Model Fit for the Binary and Ordered Categorical

(Ordinal) Outcomes**

Pearson Chi-Square

Value 29043.072

Degrees of Freedom 77833

P-Value 1.0000

Likelihood Ratio Chi-Square

Value 18411.543

Degrees of Freedom 77833

P-Value 1.0000

** Of the 209755 cells in the latent class indicator table, 147

were deleted in the calculation of chi-square due to extreme values.

Chi-Square Test for MCAR under the Unrestricted Latent Class Indicator Model

Pearson Chi-Square

Value 21568.343

Degrees of Freedom 131612

P-Value 1.0000

Likelihood Ratio Chi-Square

Value 20791.105

Degrees of Freedom 131612

P-Value 1.0000

FINAL CLASS COUNTS AND PROPORTIONS FOR THE LATENT CLASSES

BASED ON THE ESTIMATED MODEL

Latent

Classes

1 1596.67186 0.39048

2 603.33222 0.14755

3 239.08024 0.05847

4 1124.79237 0.27508

5 525.12331 0.12842

FINAL CLASS COUNTS AND PROPORTIONS FOR THE LATENT CLASSES

BASED ON ESTIMATED POSTERIOR PROBABILITIES

Latent

Classes

1 1596.67186 0.39048

2 603.33222 0.14755

3 239.08024 0.05847

4 1124.79237 0.27508

5 525.12331 0.12842

FINAL CLASS COUNTS AND PROPORTIONS FOR THE LATENT CLASSES

BASED ON THEIR MOST LIKELY LATENT CLASS MEMBERSHIP

Class Counts and Proportions

Latent

Classes

1 1648 0.40305

2 586 0.14330

3 236 0.05779

4 1139 0.27856

5 480 0.11730

CLASSIFICATION QUALITY

Entropy 0.836

Average Latent Class Probabilities for Most Likely Latent Class Membership (Row)

by Latent Class (Column)

1 2 3 4 5

1 0.920 0.001 0.001 0.029 0.050

2 0.000 0.890 0.038 0.064 0.008

3 0.000 0.083 0.909 0.001 0.007

4 0.026 0.049 0.000 0.888 0.037

5 0.106 0.011 0.002 0.059 0.821

Classification Probabilities for the Most Likely Latent Class Membership (Column)

by Latent Class (Row)

1 2 3 4 5

1 0.950 0.000 0.000 0.018 0.032

2 0.001 0.865 0.032 0.093 0.009

3 0.004 0.094 0.899 0.000 0.003

4 0.042 0.033 0.000 0.899 0.025

5 0.158 0.009 0.003 0.080 0.750

Logits for the Classification Probabilities for the Most Likely Latent Class Membership (Column)

by Latent Class (Row)

1 2 3 4 5

1 3.394 -6.969 -6.193 -0.545 0.000

2 -1.884 4.561 1.275 2.326 0.000

3 0.050 3.320 5.579 -1.927 0.000

4 0.510 0.268 -4.886 3.570 0.000

5 -1.559 -4.465 -5.505 -2.236 0.000

MODEL RESULTS

Two-Tailed

Estimate S.E. Est./S.E. P-Value

Latent Class 1

Thresholds

PROTCHLD$1 -5.789 0.501 -11.555 0.000

PROTCHLD$2 -5.379 0.415 -12.965 0.000

PROTCHLD$3 -4.688 0.321 -14.583 0.000

PROTCHLD$4 -2.838 0.168 -16.848 0.000

DRRECVAX$1 -5.430 0.418 -12.986 0.000

DRRECVAX$2 -4.581 0.273 -16.764 0.000

DRRECVAX$3 -2.933 0.140 -20.936 0.000

DRRECVAX$4 -0.955 0.080 -11.979 0.000

NEWVAXSF$1 -4.192 0.316 -13.274 0.000

NEWVAXSF$2 -3.606 0.251 -14.362 0.000

NEWVAXSF$3 -2.594 0.159 -16.309 0.000

NEWVAXSF$4 -0.421 0.072 -5.815 0.000

VAXACHV$1 -5.185 0.526 -9.857 0.000

VAXACHV$2 -4.899 0.426 -11.493 0.000

VAXACHV$3 -3.960 0.283 -13.973 0.000

VAXACHV$4 -1.923 0.132 -14.569 0.000

CNCSDEFF$1 -3.873 0.362 -10.708 0.000

CNCSDEFF$2 -1.756 0.179 -9.791 0.000

CNCSDEFF$3 -0.702 0.149 -4.723 0.000

CNCSDEFF$4 0.844 0.116 7.271 0.000

VAXAUT$1 -6.016 0.766 -7.855 0.000

VAXAUT$2 -6.016 0.766 -7.855 0.000

VAXAUT$3 -2.382 0.279 -8.525 0.000

VAXAUT$4 -1.245 0.198 -6.277 0.000

PRNTREFU$1 -4.305 0.307 -14.042 0.000

PRNTREFU$2 -2.839 0.161 -17.655 0.000

PRNTREFU$3 -1.935 0.159 -12.187 0.000

PRNTREFU$4 -0.285 0.113 -2.530 0.011

Latent Class 2

Thresholds

PROTCHLD$1 -3.621 0.426 -8.496 0.000

PROTCHLD$2 -1.852 0.226 -8.200 0.000

PROTCHLD$3 0.607 0.251 2.417 0.016

PROTCHLD$4 4.178 1.028 4.066 0.000

DRRECVAX$1 -3.920 0.724 -5.413 0.000

DRRECVAX$2 -1.185 0.204 -5.813 0.000

DRRECVAX$3 0.863 0.222 3.887 0.000

DRRECVAX$4 2.977 0.366 8.137 0.000

NEWVAXSF$1 -2.385 0.402 -5.928 0.000

NEWVAXSF$2 -0.608 0.179 -3.392 0.001

NEWVAXSF$3 0.872 0.163 5.340 0.000

NEWVAXSF$4 1.787 0.187 9.567 0.000

VAXACHV$1 -3.689 0.681 -5.420 0.000

VAXACHV$2 -1.647 0.248 -6.634 0.000

VAXACHV$3 1.031 0.260 3.962 0.000

VAXACHV$4 3.985 0.654 6.095 0.000

CNCSDEFF$1 -0.069 0.190 -0.362 0.718

CNCSDEFF$2 1.426 0.175 8.163 0.000

CNCSDEFF$3 3.839 0.464 8.283 0.000

CNCSDEFF$4 4.244 0.588 7.213 0.000

VAXAUT$1 -2.091 0.261 -8.009 0.000

VAXAUT$2 -0.575 0.159 -3.609 0.000

VAXAUT$3 1.991 0.229 8.682 0.000

VAXAUT$4 3.015 0.396 7.613 0.000

PRNTREFU$1 -0.427 0.213 -2.003 0.045

PRNTREFU$2 0.963 0.178 5.422 0.000

PRNTREFU$3 2.936 0.354 8.284 0.000

PRNTREFU$4 4.644 0.808 5.750 0.000

Latent Class 3

Thresholds

PROTCHLD$1 -0.141 0.373 -0.377 0.706

PROTCHLD$2 0.734 0.452 1.623 0.105

PROTCHLD$3 1.971 0.421 4.679 0.000

PROTCHLD$4 2.394 0.477 5.018 0.000

DRRECVAX$1 -0.279 0.294 -0.949 0.343

DRRECVAX$2 1.037 0.366 2.831 0.005

DRRECVAX$3 2.810 0.793 3.543 0.000

DRRECVAX$4 3.144 0.691 4.551 0.000

NEWVAXSF$1 0.144 0.214 0.672 0.502

NEWVAXSF$2 0.754 0.224 3.368 0.001

NEWVAXSF$3 1.561 0.288 5.422 0.000

NEWVAXSF$4 1.848 0.324 5.697 0.000

VAXACHV$1 0.119 0.364 0.327 0.743

VAXACHV$2 1.504 0.583 2.580 0.010

VAXACHV$3 3.646 1.235 2.951 0.003

VAXACHV$4 5.399 3.818 1.414 0.157

CNCSDEFF$1 1.452 0.260 5.586 0.000

CNCSDEFF$2 1.867 0.274 6.809 0.000

CNCSDEFF$3 2.484 0.324 7.655 0.000

CNCSDEFF$4 3.234 0.400 8.080 0.000

VAXAUT$1 -0.147 0.257 -0.573 0.567

VAXAUT$2 0.467 0.233 2.006 0.045

VAXAUT$3 1.788 0.310 5.772 0.000

VAXAUT$4 2.423 0.371 6.525 0.000

PRNTREFU$1 1.579 0.264 5.982 0.000

PRNTREFU$2 1.969 0.307 6.410 0.000

PRNTREFU$3 2.281 0.351 6.504 0.000

PRNTREFU$4 2.752 0.435 6.321 0.000

Latent Class 4

Thresholds

PROTCHLD$1 -15.000 0.000 999.000 999.000

PROTCHLD$2 -5.401 0.718 -7.524 0.000

PROTCHLD$3 -3.360 0.310 -10.823 0.000

PROTCHLD$4 1.431 0.194 7.395 0.000

DRRECVAX$1 -15.000 0.000 999.000 999.000

DRRECVAX$2 -3.396 0.279 -12.178 0.000

DRRECVAX$3 -1.534 0.135 -11.348 0.000

DRRECVAX$4 2.410 0.276 8.730 0.000

NEWVAXSF$1 -5.477 0.974 -5.621 0.000

NEWVAXSF$2 -2.539 0.188 -13.474 0.000

NEWVAXSF$3 -0.887 0.109 -8.135 0.000

NEWVAXSF$4 2.000 0.153 13.075 0.000

VAXACHV$1 -7.186 1.174 -6.120 0.000

VAXACHV$2 -3.665 0.291 -12.613 0.000

VAXACHV$3 -1.994 0.168 -11.837 0.000

VAXACHV$4 2.145 0.194 11.048 0.000

CNCSDEFF$1 -2.166 0.211 -10.247 0.000

CNCSDEFF$2 0.201 0.112 1.793 0.073

CNCSDEFF$3 1.571 0.141 11.170 0.000

CNCSDEFF$4 3.846 0.352 10.910 0.000

VAXAUT$1 -5.275 0.823 -6.411 0.000

VAXAUT$2 -2.174 0.176 -12.336 0.000

VAXAUT$3 0.277 0.112 2.467 0.014

VAXAUT$4 1.699 0.142 11.986 0.000

PRNTREFU$1 -3.004 0.314 -9.579 0.000

PRNTREFU$2 -0.809 0.128 -6.309 0.000

PRNTREFU$3 0.348 0.113 3.072 0.002

PRNTREFU$4 2.599 0.187 13.924 0.000

Latent Class 5

Thresholds

PROTCHLD$1 -6.145 1.366 -4.498 0.000

PROTCHLD$2 -5.240 1.141 -4.591 0.000

PROTCHLD$3 -3.850 0.654 -5.890 0.000

PROTCHLD$4 -1.767 0.336 -5.257 0.000

DRRECVAX$1 -4.114 0.621 -6.625 0.000

DRRECVAX$2 -4.114 0.621 -6.625 0.000

DRRECVAX$3 -2.326 0.300 -7.761 0.000

DRRECVAX$4 -0.897 0.182 -4.934 0.000

NEWVAXSF$1 -4.142 0.909 -4.557 0.000

NEWVAXSF$2 -2.892 0.389 -7.431 0.000

NEWVAXSF$3 -1.623 0.233 -6.963 0.000

NEWVAXSF$4 -0.617 0.243 -2.543 0.011

VAXACHV$1 -5.049 0.780 -6.470 0.000

VAXACHV$2 -5.049 0.780 -6.470 0.000

VAXACHV$3 -3.022 0.547 -5.529 0.000

VAXACHV$4 -1.350 0.251 -5.376 0.000

CNCSDEFF$1 -0.558 0.373 -1.496 0.135

CNCSDEFF$2 0.882 0.346 2.551 0.011

CNCSDEFF$3 2.687 0.648 4.146 0.000

CNCSDEFF$4 6.109 3.815 1.602 0.109

VAXAUT$1 -1.894 0.379 -5.001 0.000

VAXAUT$2 -1.144 0.359 -3.192 0.001

VAXAUT$3 1.102 0.448 2.460 0.014

VAXAUT$4 1.981 0.441 4.491 0.000

PRNTREFU$1 -1.140 0.370 -3.082 0.002

PRNTREFU$2 -0.357 0.344 -1.040 0.298

PRNTREFU$3 0.687 0.385 1.786 0.074

PRNTREFU$4 2.014 0.346 5.820 0.000

Categorical Latent Variables

Means

C#1 1.112 0.284 3.911 0.000

C#2 0.139 0.235 0.592 0.554

C#3 -0.787 0.295 -2.664 0.008

C#4 0.762 0.252 3.024 0.002

RESULTS IN PROBABILITY SCALE

Latent Class 1

PROTCHLD

Category 1 0.003 0.002 2.002 0.045

Category 2 0.002 0.001 1.367 0.172

Category 3 0.005 0.002 2.061 0.039

Category 4 0.046 0.008 5.521 0.000

Category 5 0.945 0.009 107.360 0.000

DRRECVAX

Category 1 0.004 0.002 2.402 0.016

Category 2 0.006 0.002 2.828 0.005

Category 3 0.040 0.006 6.599 0.000

Category 4 0.227 0.015 15.308 0.000

Category 5 0.722 0.016 45.141 0.000

NEWVAXSF

Category 1 0.015 0.005 3.214 0.001

Category 2 0.012 0.004 2.615 0.009

Category 3 0.043 0.007 5.954 0.000

Category 4 0.327 0.016 20.878 0.000

Category 5 0.604 0.017 34.861 0.000

VAXACHV

Category 1 0.006 0.003 1.912 0.056

Category 2 0.002 0.001 1.527 0.127

Category 3 0.011 0.004 2.706 0.007

Category 4 0.109 0.014 8.059 0.000

Category 5 0.872 0.015 59.410 0.000

CNCSDEFF

Category 1 0.020 0.007 2.822 0.005

Category 2 0.127 0.019 6.618 0.000

Category 3 0.184 0.017 10.846 0.000

Category 4 0.368 0.018 20.994 0.000

Category 5 0.301 0.024 12.320 0.000

VAXAUT

Category 1 0.002 0.002 1.309 0.191

Category 2 0.000 0.000 0.000 1.000

Category 3 0.082 0.021 3.905 0.000

Category 4 0.139 0.017 8.166 0.000

Category 5 0.776 0.034 22.552 0.000

PRNTREFU

Category 1 0.013 0.004 3.305 0.001

Category 2 0.042 0.007 5.990 0.000

Category 3 0.071 0.013 5.263 0.000

Category 4 0.303 0.018 16.752 0.000

Category 5 0.571 0.028 20.670 0.000

Latent Class 2

PROTCHLD

Category 1 0.026 0.011 2.409 0.016

Category 2 0.110 0.023 4.764 0.000

Category 3 0.512 0.047 10.896 0.000

Category 4 0.338 0.059 5.701 0.000

Category 5 0.015 0.015 0.988 0.323

DRRECVAX

Category 1 0.019 0.014 1.408 0.159

Category 2 0.215 0.031 7.039 0.000

Category 3 0.469 0.035 13.510 0.000

Category 4 0.248 0.046 5.451 0.000

Category 5 0.048 0.017 2.872 0.004

NEWVAXSF

Category 1 0.084 0.031 2.714 0.007

Category 2 0.268 0.028 9.538 0.000

Category 3 0.353 0.034 10.283 0.000

Category 4 0.151 0.029 5.225 0.000

Category 5 0.143 0.023 6.250 0.000

VAXACHV

Category 1 0.024 0.016 1.506 0.132

Category 2 0.137 0.024 5.642 0.000

Category 3 0.576 0.040 14.309 0.000

Category 4 0.245 0.048 5.068 0.000

Category 5 0.018 0.012 1.558 0.119

CNCSDEFF

Category 1 0.483 0.048 10.152 0.000

Category 2 0.323 0.039 8.321 0.000

Category 3 0.173 0.027 6.288 0.000

Category 4 0.007 0.005 1.313 0.189

Category 5 0.014 0.008 1.724 0.085

VAXAUT

Category 1 0.110 0.026 4.304 0.000

Category 2 0.250 0.027 9.119 0.000

Category 3 0.520 0.036 14.628 0.000

Category 4 0.073 0.017 4.288 0.000

Category 5 0.047 0.018 2.649 0.008

PRNTREFU

Category 1 0.395 0.051 7.751 0.000

Category 2 0.329 0.033 9.867 0.000

Category 3 0.226 0.031 7.299 0.000

Category 4 0.041 0.014 2.825 0.005

Category 5 0.010 0.008 1.250 0.211

Latent Class 3

PROTCHLD

Category 1 0.465 0.093 5.009 0.000

Category 2 0.211 0.042 4.965 0.000

Category 3 0.202 0.079 2.546 0.011

Category 4 0.039 0.023 1.656 0.098

Category 5 0.084 0.037 2.288 0.022

DRRECVAX

Category 1 0.431 0.072 5.963 0.000

Category 2 0.308 0.049 6.276 0.000

Category 3 0.205 0.054 3.822 0.000

Category 4 0.015 0.024 0.651 0.515

Category 5 0.041 0.027 1.510 0.131

NEWVAXSF

Category 1 0.536 0.053 10.058 0.000

Category 2 0.144 0.037 3.912 0.000

Category 3 0.146 0.037 3.998 0.000

Category 4 0.037 0.019 2.008 0.045

Category 5 0.136 0.038 3.568 0.000

VAXACHV

Category 1 0.530 0.091 5.846 0.000

Category 2 0.288 0.045 6.419 0.000

Category 3 0.156 0.068 2.299 0.022

Category 4 0.021 0.024 0.890 0.373

Category 5 0.005 0.017 0.263 0.792

CNCSDEFF

Category 1 0.810 0.040 20.281 0.000

Category 2 0.056 0.029 1.905 0.057

Category 3 0.057 0.022 2.594 0.009

Category 4 0.039 0.018 2.233 0.026

Category 5 0.038 0.015 2.597 0.009

VAXAUT

Category 1 0.463 0.064 7.250 0.000

Category 2 0.151 0.043 3.547 0.000

Category 3 0.242 0.048 5.063 0.000

Category 4 0.062 0.028 2.221 0.026

Category 5 0.081 0.028 2.932 0.003

PRNTREFU

Category 1 0.829 0.037 22.161 0.000

Category 2 0.048 0.027 1.808 0.071

Category 3 0.030 0.016 1.820 0.069

Category 4 0.033 0.014 2.262 0.024

Category 5 0.060 0.025 2.444 0.015

Latent Class 4

PROTCHLD

Category 1 0.000 0.000 0.000 1.000

Category 2 0.004 0.003 1.399 0.162

Category 3 0.029 0.009 3.171 0.002

Category 4 0.773 0.028 27.639 0.000

Category 5 0.193 0.030 6.403 0.000

DRRECVAX

Category 1 0.000 0.000 0.000 1.000

Category 2 0.032 0.009 3.706 0.000

Category 3 0.145 0.017 8.748 0.000

Category 4 0.740 0.023 32.613 0.000

Category 5 0.082 0.021 3.948 0.000

NEWVAXSF

Category 1 0.004 0.004 1.031 0.303

Category 2 0.069 0.012 5.980 0.000

Category 3 0.219 0.018 11.867 0.000

Category 4 0.589 0.025 23.730 0.000

Category 5 0.119 0.016 7.423 0.000

VAXACHV

Category 1 0.001 0.001 0.852 0.394

Category 2 0.024 0.007 3.465 0.001

Category 3 0.095 0.016 6.096 0.000

Category 4 0.775 0.020 38.430 0.000

Category 5 0.105 0.018 5.753 0.000

CNCSDEFF

Category 1 0.103 0.020 5.272 0.000

Category 2 0.447 0.022 20.752 0.000

Category 3 0.278 0.019 14.312 0.000

Category 4 0.151 0.017 8.723 0.000

Category 5 0.021 0.007 2.898 0.004

VAXAUT

Category 1 0.005 0.004 1.222 0.222

Category 2 0.097 0.015 6.427 0.000

Category 3 0.467 0.023 20.051 0.000

Category 4 0.277 0.020 13.867 0.000

Category 5 0.155 0.019 8.343 0.000

PRNTREFU

Category 1 0.047 0.014 3.347 0.001

Category 2 0.261 0.022 11.922 0.000

Category 3 0.278 0.019 14.644 0.000

Category 4 0.345 0.023 14.782 0.000

Category 5 0.069 0.012 5.755 0.000

Latent Class 5

PROTCHLD

Category 1 0.002 0.003 0.733 0.463

Category 2 0.003 0.004 0.776 0.438

Category 3 0.016 0.011 1.468 0.142

Category 4 0.125 0.036 3.466 0.001

Category 5 0.854 0.042 20.385 0.000

DRRECVAX

Category 1 0.016 0.010 1.637 0.102

Category 2 0.000 0.000 1.664 0.096

Category 3 0.073 0.020 3.564 0.000

Category 4 0.201 0.037 5.477 0.000

Category 5 0.710 0.037 18.987 0.000

NEWVAXSF

Category 1 0.016 0.014 1.118 0.264

Category 2 0.037 0.014 2.718 0.007

Category 3 0.112 0.024 4.579 0.000

Category 4 0.186 0.050 3.704 0.000

Category 5 0.649 0.055 11.764 0.000

VAXACHV

Category 1 0.006 0.005 1.290 0.197

Category 2 0.000 0.000 1.298 0.194

Category 3 0.040 0.023 1.706 0.088

Category 4 0.159 0.037 4.351 0.000

Category 5 0.794 0.041 19.345 0.000

CNCSDEFF

Category 1 0.364 0.086 4.221 0.000

Category 2 0.343 0.039 8.722 0.000

Category 3 0.229 0.046 4.986 0.000

Category 4 0.062 0.039 1.582 0.114

Category 5 0.002 0.008 0.263 0.793

VAXAUT

Category 1 0.131 0.043 3.037 0.002

Category 2 0.111 0.031 3.520 0.000

Category 3 0.509 0.041 12.287 0.000

Category 4 0.128 0.055 2.339 0.019

Category 5 0.121 0.047 2.579 0.010

PRNTREFU

Category 1 0.242 0.068 3.567 0.000

Category 2 0.169 0.031 5.424 0.000

Category 3 0.254 0.029 8.712 0.000

Category 4 0.217 0.065 3.336 0.001

Category 5 0.118 0.036 3.275 0.001

LATENT CLASS ODDS RATIO RESULTS

95% C.I.

Estimate S.E. Lower 2.5% Upper 2.5%

Latent Class 1 Compared to Latent Class 2

PROTCHLD

Category > 1 8.742 5.922 2.318 32.976

Category > 2 34.014 16.202 13.372 86.520

Category > 3 199.498 82.284 88.889 447.744

Category > 4 1114.378 1172.184 141.795 8757.997

DRRECVAX

Category > 1 4.527 3.794 0.876 23.396

Category > 2 29.832 10.170 15.293 58.194

Category > 3 44.492 11.523 26.780 73.916

Category > 4 51.024 19.284 24.326 107.025

NEWVAXSF

Category > 1 6.090 3.157 2.205 16.820

Category > 2 20.042 6.148 10.985 36.565

Category > 3 32.009 6.923 20.949 48.908

Category > 4 9.096 1.786 6.190 13.366

VAXACHV

Category > 1 4.467 3.885 0.812 24.563

Category > 2 25.860 12.800 9.802 68.226

Category > 3 147.110 55.082 70.619 306.452

Category > 4 367.999 241.835 101.499 1334.240

CNCSDEFF

Category > 1 44.880 18.844 19.708 102.201

Category > 2 24.076 6.197 14.537 39.874

Category > 3 93.823 45.639 36.161 243.430

Category > 4 29.974 17.886 9.307 96.536

VAXAUT

Category > 1 50.663 41.384 10.218 251.189

Category > 2 230.817 181.414 49.458 1077.198

Category > 3 79.294 29.120 38.604 162.871

Category > 4 70.767 31.513 29.565 169.388

PRNTREFU

Category > 1 48.341 17.919 23.376 99.966

Category > 2 44.785 10.699 28.040 71.529

Category > 3 130.383 50.558 60.974 278.801

Category > 4 138.323 112.970 27.906 685.638

Latent Class 1 Compared to Latent Class 3

PROTCHLD

Category > 1 283.827 180.870 81.398 989.684

Category > 2 451.385 282.521 132.365 1539.295

Category > 3 779.817 410.426 277.964 2187.749

Category > 4 187.202 94.259 69.777 502.240

DRRECVAX

Category > 1 172.473 88.930 62.780 473.830

Category > 2 275.192 126.313 111.925 676.618

Category > 3 311.872 251.586 64.166 1515.827

Category > 4 60.277 41.816 15.475 234.783

NEWVAXSF

Category > 1 76.406 29.066 36.250 161.044

Category > 2 78.237 26.230 40.553 150.936

Category > 3 63.715 20.865 33.535 121.057

Category > 4 9.670 3.202 5.053 18.504

VAXACHV

Category > 1 201.237 130.325 56.551 716.101

Category > 2 603.723 437.946 145.665 2502.178

Category > 3 2009.094 2560.360 165.281 24421.873

Category > 4 1512.809 5765.629 0.862 *********

CNCSDEFF

Category > 1 205.410 90.659 86.483 487.881

Category > 2 37.452 12.202 19.777 70.925

Category > 3 24.181 8.547 12.095 48.342

Category > 4 10.910 4.497 4.863 24.475

VAXAUT

Category > 1 353.903 287.195 72.130 1736.416

Category > 2 654.051 524.102 135.993 3145.614

Category > 3 64.701 26.182 29.272 143.010

Category > 4 39.162 15.681 17.865 85.845

PRNTREFU

Category > 1 359.229 147.222 160.886 802.092

Category > 2 122.438 42.579 61.930 242.066

Category > 3 67.760 25.966 31.974 143.601

Category > 4 20.848 9.281 8.712 49.888

Latent Class 1 Compared to Latent Class 4

PROTCHLD

Category > 1 0.000 0.000 0.000 0.000

Category > 2 0.978 0.850 0.178 5.369

Category > 3 3.777 1.729 1.539 9.266

Category > 4 71.472 15.645 46.538 109.766

DRRECVAX

Category > 1 0.000 0.000 0.000 0.000

Category > 2 3.269 1.311 1.489 7.175

Category > 3 4.049 0.785 2.769 5.920

Category > 4 28.927 7.887 16.951 49.362

NEWVAXSF

Category > 1 0.277 0.281 0.038 2.027

Category > 2 2.906 0.910 1.573 5.369

Category > 3 5.511 1.043 3.803 7.985

Category > 4 11.254 1.918 8.059 15.717

VAXACHV

Category > 1 0.135 0.178 0.010 1.788

Category > 2 3.437 1.819 1.218 9.696

Category > 3 7.138 2.335 3.759 13.554

Category > 4 58.460 12.587 38.333 89.154

CNCSDEFF

Category > 1 5.511 2.325 2.410 12.598

Category > 2 7.078 1.487 4.690 10.684

Category > 3 9.710 1.867 6.661 14.155

Category > 4 20.120 7.133 10.042 40.310

VAXAUT

Category > 1 2.098 2.392 0.225 19.593

Category > 2 46.629 36.873 9.898 219.669

Category > 3 14.283 4.215 8.010 25.469

Category > 4 18.994 4.316 12.167 29.652

PRNTREFU

Category > 1 3.675 1.580 1.582 8.535

Category > 2 7.611 1.548 5.109 11.340

Category > 3 9.805 1.852 6.772 14.199

Category > 4 17.891 3.705 11.923 26.848

Latent Class 1 Compared to Latent Class 5

PROTCHLD

Category > 1 0.700 1.023 0.040 12.283

Category > 2 1.149 1.421 0.102 12.985

Category > 3 2.313 1.735 0.532 10.062

Category > 4 2.921 1.131 1.367 6.240

DRRECVAX

Category > 1 3.727 2.974 0.780 17.807

Category > 2 1.595 1.126 0.400 6.362

Category > 3 1.835 0.643 0.924 3.645

Category > 4 1.059 0.218 0.707 1.586

NEWVAXSF

Category > 1 1.052 1.077 0.141 7.823

Category > 2 2.043 1.063 0.737 5.664

Category > 3 2.639 0.821 1.434 4.857

Category > 4 0.822 0.220 0.487 1.388

VAXACHV

Category > 1 1.146 1.153 0.159 8.234

Category > 2 0.861 0.810 0.136 5.447

Category > 3 2.554 1.679 0.704 9.263

Category > 4 1.774 0.522 0.997 3.157

CNCSDEFF

Category > 1 27.531 11.891 11.808 64.190

Category > 2 13.981 3.746 8.268 23.639

Category > 3 29.633 16.752 9.785 89.740

Category > 4 193.537 742.479 0.105 *********

VAXAUT

Category > 1 61.667 48.152 13.347 284.911

Category > 2 130.546 98.263 29.857 570.800

Category > 3 32.598 9.920 17.954 59.188

Category > 4 25.184 9.996 11.568 54.828

PRNTREFU

Category > 1 23.690 10.551 9.896 56.714

Category > 2 11.959 4.011 6.198 23.076

Category > 3 13.765 4.424 7.331 25.844

Category > 4 9.970 3.170 5.345 18.594

Latent Class 2 Compared to Latent Class 3

PROTCHLD

Category > 1 32.466 15.483 12.749 82.677

Category > 2 13.271 5.792 5.641 31.220

Category > 3 3.909 1.956 1.466 10.423

Category > 4 0.168 0.221 0.013 2.225

DRRECVAX

Category > 1 38.096 24.237 10.948 132.562

Category > 2 9.225 3.230 4.644 18.325

Category > 3 7.010 5.780 1.392 35.289

Category > 4 1.181 0.994 0.227 6.142

NEWVAXSF

Category > 1 12.545 5.169 5.594 28.134

Category > 2 3.904 1.103 2.243 6.792

Category > 3 1.991 0.675 1.025 3.867

Category > 4 1.063 0.431 0.481 2.351

VAXACHV

Category > 1 45.049 27.988 13.330 152.243

Category > 2 23.346 11.898 8.598 63.389

Category > 3 13.657 16.944 1.200 155.396

Category > 4 4.111 16.403 0.002 10241.734

CNCSDEFF

Category > 1 4.577 1.595 2.311 9.063

Category > 2 1.556 0.555 0.773 3.130

Category > 3 0.258 0.153 0.081 0.825

Category > 4 0.364 0.284 0.079 1.680

VAXAUT

Category > 1 6.985 2.235 3.731 13.078

Category > 2 2.834 0.806 1.623 4.948

Category > 3 0.816 0.351 0.351 1.898

Category > 4 0.553 0.334 0.169 1.808

PRNTREFU

Category > 1 7.431 2.480 3.863 14.295

Category > 2 2.734 1.093 1.249 5.984

Category > 3 0.520 0.296 0.170 1.585

Category > 4 0.151 0.148 0.022 1.031

Latent Class 2 Compared to Latent Class 4

PROTCHLD

Category > 1 0.000 0.000 0.000 0.000

Category > 2 0.029 0.022 0.007 0.125

Category > 3 0.019 0.006 0.010 0.035

Category > 4 0.064 0.069 0.008 0.530

DRRECVAX

Category > 1 0.000 0.000 0.000 0.000

Category > 2 0.110 0.037 0.057 0.211

Category > 3 0.091 0.020 0.059 0.141

Category > 4 0.567 0.279 0.216 1.485

NEWVAXSF

Category > 1 0.045 0.050 0.005 0.395

Category > 2 0.145 0.035 0.090 0.234

Category > 3 0.172 0.031 0.122 0.244

Category > 4 1.237 0.325 0.739 2.071

VAXACHV

Category > 1 0.030 0.043 0.002 0.498

Category > 2 0.133 0.051 0.063 0.282

Category > 3 0.049 0.012 0.030 0.078

Category > 4 0.159 0.109 0.041 0.610

CNCSDEFF

Category > 1 0.123 0.032 0.074 0.205

Category > 2 0.294 0.061 0.195 0.443

Category > 3 0.103 0.051 0.039 0.273

Category > 4 0.671 0.483 0.164 2.747

VAXAUT

Category > 1 0.041 0.035 0.008 0.220

Category > 2 0.202 0.046 0.129 0.317

Category > 3 0.180 0.045 0.111 0.292

Category > 4 0.268 0.115 0.116 0.621

PRNTREFU

Category > 1 0.076 0.026 0.039 0.148

Category > 2 0.170 0.034 0.115 0.252

Category > 3 0.075 0.026 0.038 0.149

Category > 4 0.129 0.109 0.025 0.671

Latent Class 2 Compared to Latent Class 5

PROTCHLD

Category > 1 0.080 0.115 0.005 1.331

Category > 2 0.034 0.039 0.003 0.331

Category > 3 0.012 0.008 0.003 0.046

Category > 4 0.003 0.003 0.000 0.020

DRRECVAX

Category > 1 0.823 0.784 0.127 5.326

Category > 2 0.053 0.035 0.015 0.191

Category > 3 0.041 0.015 0.020 0.085

Category > 4 0.021 0.008 0.009 0.046

NEWVAXSF

Category > 1 0.173 0.163 0.027 1.102

Category > 2 0.102 0.043 0.045 0.233

Category > 3 0.082 0.024 0.047 0.144

Category > 4 0.090 0.029 0.048 0.171

VAXACHV

Category > 1 0.257 0.263 0.034 1.921

Category > 2 0.033 0.027 0.007 0.164

Category > 3 0.017 0.011 0.005 0.058

Category > 4 0.005 0.003 0.001 0.019

CNCSDEFF

Category > 1 0.613 0.265 0.263 1.430

Category > 2 0.581 0.231 0.266 1.266

Category > 3 0.316 0.255 0.065 1.537

Category > 4 6.457 25.800 0.003 16266.998

VAXAUT

Category > 1 1.217 0.568 0.488 3.038

Category > 2 0.566 0.225 0.259 1.235

Category > 3 0.411 0.210 0.151 1.117

Category > 4 0.356 0.210 0.112 1.134

PRNTREFU

Category > 1 0.490 0.215 0.207 1.159

Category > 2 0.267 0.106 0.123 0.581

Category > 3 0.106 0.057 0.037 0.304

Category > 4 0.072 0.063 0.013 0.404

Latent Class 3 Compared to Latent Class 4

PROTCHLD

Category > 1 0.000 0.000 0.000 0.000

Category > 2 0.002 0.002 0.000 0.010

Category > 3 0.005 0.002 0.002 0.013

Category > 4 0.382 0.198 0.138 1.055

DRRECVAX

Category > 1 0.000 0.000 0.000 0.000

Category > 2 0.012 0.005 0.005 0.028

Category > 3 0.013 0.010 0.003 0.062

Category > 4 0.480 0.370 0.106 2.175

NEWVAXSF

Category > 1 0.004 0.004 0.001 0.025

Category > 2 0.037 0.011 0.021 0.065

Category > 3 0.086 0.026 0.048 0.156

Category > 4 1.164 0.414 0.579 2.339

VAXACHV

Category > 1 0.001 0.001 0.000 0.008

Category > 2 0.006 0.004 0.002 0.020

Category > 3 0.004 0.004 0.000 0.040

Category > 4 0.039 0.149 0.000 75.440

CNCSDEFF

Category > 1 0.027 0.009 0.014 0.052

Category > 2 0.189 0.058 0.104 0.344

Category > 3 0.402 0.147 0.196 0.822

Category > 4 1.844 0.968 0.659 5.160

VAXAUT

Category > 1 0.006 0.005 0.001 0.031

Category > 2 0.071 0.020 0.041 0.123

Category > 3 0.221 0.074 0.115 0.425

Category > 4 0.485 0.194 0.222 1.062

PRNTREFU

Category > 1 0.010 0.004 0.005 0.023

Category > 2 0.062 0.022 0.031 0.123

Category > 3 0.145 0.056 0.068 0.309

Category > 4 0.858 0.415 0.333 2.214

Latent Class 3 Compared to Latent Class 5

PROTCHLD

Category > 1 0.002 0.003 0.000 0.036

Category > 2 0.003 0.003 0.000 0.024

Category > 3 0.003 0.002 0.001 0.012

Category > 4 0.016 0.009 0.005 0.048

DRRECVAX

Category > 1 0.022 0.014 0.006 0.080

Category > 2 0.006 0.004 0.002 0.022

Category > 3 0.006 0.005 0.001 0.029

Category > 4 0.018 0.013 0.004 0.072

NEWVAXSF

Category > 1 0.014 0.013 0.002 0.088

Category > 2 0.026 0.012 0.011 0.063

Category > 3 0.041 0.016 0.020 0.086

Category > 4 0.085 0.035 0.038 0.190

VAXACHV

Category > 1 0.006 0.005 0.001 0.029

Category > 2 0.001 0.001 0.000 0.009

Category > 3 0.001 0.002 0.000 0.015

Category > 4 0.001 0.004 0.000 2.145

CNCSDEFF

Category > 1 0.134 0.060 0.056 0.323

Category > 2 0.373 0.164 0.158 0.881

Category > 3 1.225 0.885 0.297 5.050

Category > 4 17.739 68.504 0.009 34361.914

VAXAUT

Category > 1 0.174 0.079 0.072 0.424

Category > 2 0.200 0.084 0.088 0.454

Category > 3 0.504 0.272 0.175 1.449

Category > 4 0.643 0.390 0.196 2.112

PRNTREFU

Category > 1 0.066 0.029 0.028 0.155

Category > 2 0.098 0.043 0.041 0.231

Category > 3 0.203 0.102 0.076 0.542

Category > 4 0.478 0.268 0.159 1.435

Latent Class 4 Compared to Latent Class 5

PROTCHLD

Category > 1 7005.856 0.000 7005.856 7005.856

Category > 2 1.175 1.623 0.078 17.628

Category > 3 0.612 0.459 0.141 2.659

Category > 4 0.041 0.017 0.018 0.091

DRRECVAX

Category > 1 53423.875 0.000 53423.875 53423.875

Category > 2 0.488 0.342 0.123 1.929

Category > 3 0.453 0.158 0.229 0.896

Category > 4 0.037 0.012 0.019 0.069

NEWVAXSF

Category > 1 3.802 5.684 0.203 71.222

Category > 2 0.703 0.320 0.288 1.717

Category > 3 0.479 0.131 0.281 0.817

Category > 4 0.073 0.020 0.043 0.124

VAXACHV

Category > 1 8.474 12.115 0.514 139.646

Category > 2 0.251 0.210 0.048 1.297

Category > 3 0.358 0.216 0.109 1.170

Category > 4 0.030 0.009 0.017 0.053

CNCSDEFF

Category > 1 4.996 2.159 2.142 11.652

Category > 2 1.975 0.721 0.966 4.040

Category > 3 3.052 1.996 0.847 10.995

Category > 4 9.619 37.301 0.005 19227.312

VAXAUT

Category > 1 29.388 26.910 4.883 176.854

Category > 2 2.800 1.127 1.272 6.164

Category > 3 2.282 1.051 0.926 5.626

Category > 4 1.326 0.616 0.534 3.294

PRNTREFU

Category > 1 6.446 3.223 2.419 17.177

Category > 2 1.571 0.592 0.751 3.287

Category > 3 1.404 0.571 0.633 3.113

Category > 4 0.557 0.226 0.252 1.232

QUALITY OF NUMERICAL RESULTS

Condition Number for the Information Matrix 0.936E-04

(ratio of smallest to largest eigenvalue)

TECHNICAL 11 OUTPUT

Random Starts Specifications for the k-1 Class Analysis Model

Number of initial stage random starts 250

Number of final stage optimizations 250

VUONG-LO-MENDELL-RUBIN LIKELIHOOD RATIO TEST FOR 4 (H0) VERSUS 5 CLASSES

H0 Loglikelihood Value -32841.832

2 Times the Loglikelihood Difference 767.992

Difference in the Number of Parameters 29

Mean 29064.152

Standard Deviation 40734.136

P-Value 0.7564

LO-MENDELL-RUBIN ADJUSTED LRT TEST

Value 764.820

P-Value 0.7564

DIAGRAM INFORMATION

Mplus diagrams are currently not available for Mixture analysis.

No diagram output was produced.

Beginning Time: 19:06:46

Ending Time: 19:08:42

Elapsed Time: 00:01:56

MUTHEN & MUTHEN

3463 Stoner Ave.

Los Angeles, CA 90066

Tel: (310) 391-9971

Fax: (310) 391-8971

Web: www.StatModel.com

Support: Support@StatModel.com

Copyright (c) 1998-2020 Muthen & Muthen
